# 2-Alkylation of 3-Alkyindoles With Unactivated Alkenes

**DOI:** 10.3389/fchem.2022.860764

**Published:** 2022-02-24

**Authors:** Xuling Pan, Qian Liu, Yingling Nong

**Affiliations:** State Key Laboratory Breeding Base of Green Pesticide and Agricultural Bioengineering, Key Laboratory of Green Pesticide and Agricultural Bioengineering Ministry of Education, Guizhou University, Guiyang, China

**Keywords:** metal-free, acid catalysis, atom economy, indole-2-alkylation, alkene

## Abstract

An acid-catalyzed 2-alkylation of indole molecules is developed. Only catalytic amount of the commercially available, inexpensive and traceless HI is used as the sole reaction promoter. 2,3-Disubstituted indole molecules bearing congested tertiary carbon centers are afforded as the final products in moderate to excellent yields.

## Introduction

**FIGURE 1:**
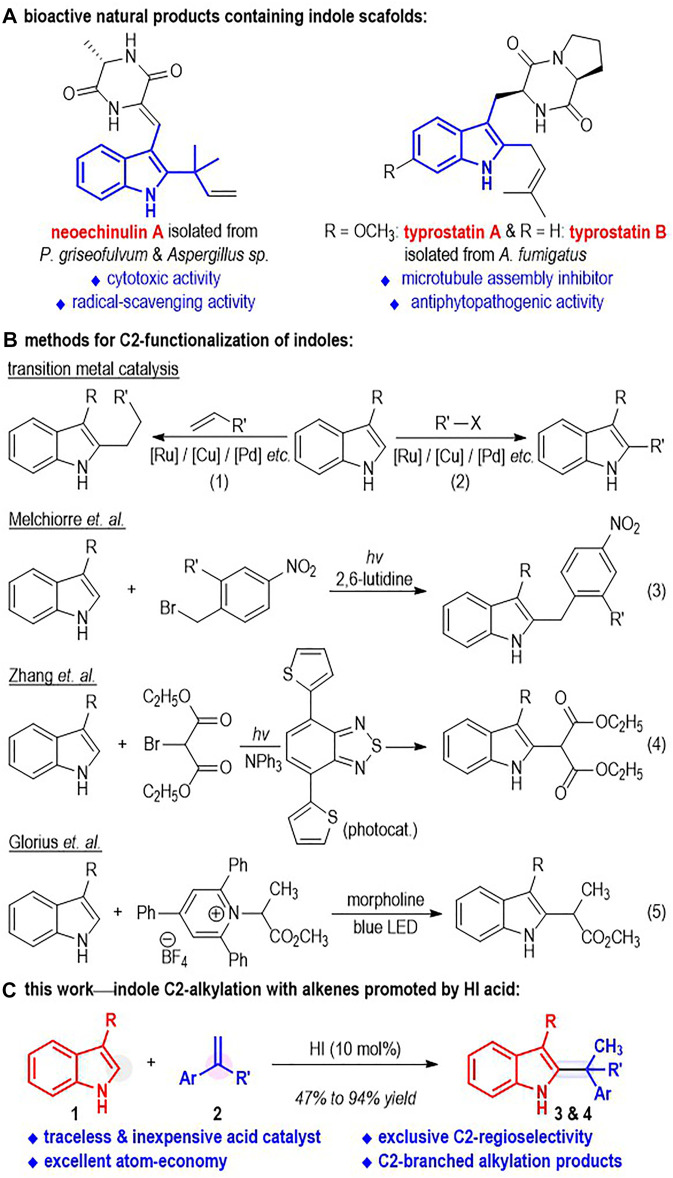
Bioactive Indole Derivatives bearing C2-Substituents and C2-Functionalizations of Unprotected Indoles.

Indole and its derivatives are versatile molecules and have found significant applications in biological and medicinal research (Ma et al., 2016) (Figure 1A). For example, the indole derivative neoechinulin A has been isolated from *P. griseofulvum* and *Aspergillus sp*. and shown significant cytotoxic activity against P388 cells (Ma et al., 2016). Typrostatin A and B can be obtained from *A. fumigatus*, which is present in the sea sediment and the *Ficus* carica in both Japan and China. They have exhibited excellent antiphytopathogenic activity and have been used in the investigation of novel anti-tumor reagents (Ma et al., 2016). Therefore, the development of simple and efficient methods for functionalization of indole molecules is attractive and important to both scientific research and drug manufacturing.

Indole can be functionalized at each position around its aromatic structure through various transformations. Traditionally, the C3-positions of indole molecules have been widely used as nucleophiles to react with a variety of electrophiles in both enantioselective (Austin and MacMillan et al., 2002; Zhou and Tang et al., 2002; Evans et al., 2005; Wang et al., 2006; Terada et al., 2007; Bandini et al., 2009; Joucla and Djakovitch et al., 2009; Bartoli et al., 2010) and non-chiral fashion (Bartoli et al., 2005; Kimura et al., 2005; Moran et al., 2006; Kusurkar et al., 2008; Lerch et al., 2014). Transition metals, amines and Lewis acids are frequently used as effective catalysts for these reactions. The C2-positions of the *N*-protected indoles can undergo transition metal-catalyzed alkylations (Doyle et al., 2010; Johansen and Kerr et al., 2010; Jiao and Bach et al., 2011; Pan et al., 2012; Lin et al., 2013; Yoshino et al., 2013; Su et al., 2014; Soni et al., 2018; Wang and Wang, 2021), arylations (Lane and Sames, 2004; Wang et al., 2005; Deprez et al., 2006; Lebrasseur and Larrosa et al., 2008; Phipps et al., 2008; Yang et al., 2008; Ackermann and Lygin, 2011; Sauermann et al., 2011; Li et al., 2016; Yang and Shi, 2018), alkenylations (Nakao et al., 2006; Maehara et al., 2008; Ding and Yoshikai, 2012; Li et al., 2013a; Li et al., 2013b; Liang et al., 2014; Schramm et al., 2015; Wong et al., 2015; Zhang W et al., 2015; Zhou et al., 2016), alkynylations (Yang et al., 2010; Tolnai et al., 2013; Sauermann et al., 2015; Zhang Z Z et al., 2015; Kong et al., 2016), aminations (Sun et al., 2014; Sun et al., 2015) and thiolation reactions (Gensch et al., 2016). A directing group is generally required to be installed on the *N* atom of the indole molecule for the C2-functional group introductions in these transformations. In contrast, intermolecular reactions for direct functionalization of the C2-positions of the unprotected indoles have been relatively less developed (Figure 1B). Success within this direction includes the transition metal-catalyzed indole C2-alkylations with alkyl halides (Straathof et al., 2014; Shao et al., 2015; Yang et al., 2015) and alkenes (Weng et al., 2016; Zhou et al., 2017; Bai et al., 2020) (Figure 1B, eq. 1 and eq. 2). Melchiorre (Kandukuri et al., 2015) and co-workers have disclosed the formation of the electron donor-acceptor (EDA) complex between indoles and electron-deficient benzylbromides and reported a metal-free photo-catalyzed direct indole C2-alkylation *via* formation of this EDA complex (eq. 3). Zhang (Wang et al., 2016) and co-workers reported in 2016 the organic semiconductor-catalyzed, visible light-promoted indole C2-alkylation with 2-bromomalanates (eq. 4). Recently, Glorius (James et al., 2019) and co-workers used the pyridinium salt as the EDA complex acceptor and realized the indole C2-alkylation reaction under basic conditions with white light irradiation (eq. 5). To the best of our knowledge, the direct and metal-free C2-alkylation of indoles with unactivated alkenes has not been disclosed.

Herein, we report an acid-promoted regioselective C2-alkylation reaction of unprotected indoles **1** (Figure 1C). Unactivated alkenes **2** are used as the alkylation reagent, with no sacrificing atoms or functional groups lost during this transformation. The use of EDA acceptors can be avoided in this protocol. A traceless and inexpensive HI is used in a catalytic amount as the sole reaction catalyst. The C2-branched alkylation products **3** or **4** bearing a tertiary carbon center are afforded in excellent regioselective fashion with good to excellent isolated yields. The reaction features excellent atom-economy and C2-regioselectivity.

The reaction was initially carried out by using 3-methylindole **1a** and 1,1-diphenylethene **2a** as the substrates in dichloromethane under the catalysis of different organic and inorganic acids (Table 1, entries 1–7). To our delight, the indole C2-branched alkylation product **3a** can be obtained in promising yields with a variety of strong acids after stirring at 30°C for 12 h (entries 1–6). The target product of **3a** could be afforded in 21% yield in the presence of 30 mol% of HCl acid (entry 1). The yield of **3a** could be dramatically improved to 87% when switching the HCl into HBr (entry 2). Gratifyingly, the product **3a** was obtained in 92% yield when using HI as the acid catalyst (entry 3). Other organic or inorganic acids gave the desired product **3a** in lower yields (entries 4–6). Notably, the reaction could not happen when using acetic acid as the reaction catalyst, which is probably due to its weak acidity (entry 7). A diversity of organic solvents could be used as the reaction mediate (entries 8–11). For example, the reaction went on smoothly in the solvents of EtOAc, hexane and toluene, with the desired product **3a** afforded in good yields (entries 8–10). However, solvents with high polarities such as H_2_O, DMF, THF, CH_3_OH and MTBE could not be used for this transformation (entry 11). The amount of the HI catalyst could be decreased to 0.1 mol% without obvious erosion of the product yield (entries 12–13). Further decreasing the amount of the HI to 0.05 mol% resulted in significant loss of the yield (entry 14). The reaction temperature could also be decreased to 25°C with the desired product of **3a** afforded in an even higher yield (entry 15).

**TABLE 1:** Optimization of reaction conditions.^a^ 
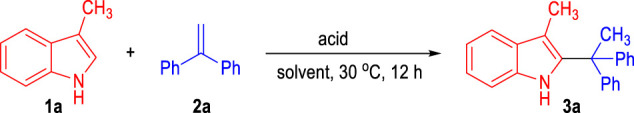

Entry	Acid	Solvent	Equiv	Yield (%)^b^
1	HCl	CH_2_Cl_2_	0.3	21
2	HBr	CH_2_Cl_2_	0.3	87
3	HI	CH_2_Cl_2_	0.3	92
4	H_2_SO_4_	CH_2_Cl_2_	0.3	30
5	TsOH	CH_2_Cl_2_	0.3	74
6	TFA	CH_2_Cl_2_	0.3	27
7	CH_3_CO_2_H	CH_2_Cl_2_	0.3	0
8	HI	EtOAc	0.3	80
9	HI	hexane	0.3	73
10	HI	toluene	0.3	88
11	HI	H_2_O/DMF/THF/CH_3_OH/MTBE	0.3	0
12	HI	CH_2_Cl_2_	0.2	94
13	HI	CH_2_Cl_2_	0.1	93
14	HI	CH_2_Cl_2_	0.05	67
15^c^	HI	CH_2_Cl_2_	0.1	94

a Reaction conditions: unless otherwise stated, the reaction of 3-methylindole **1a** (0.11 mmol), 1,1-diphenylethene **2a** (0.10 mmol) and HI (0.01 mmol) was carried out at 30°C in CH_2_Cl_2_ (1.0 ml) for 12 h.

b Isolated yield of **3a**.

c at 25°C.

**SCHEME 1:**
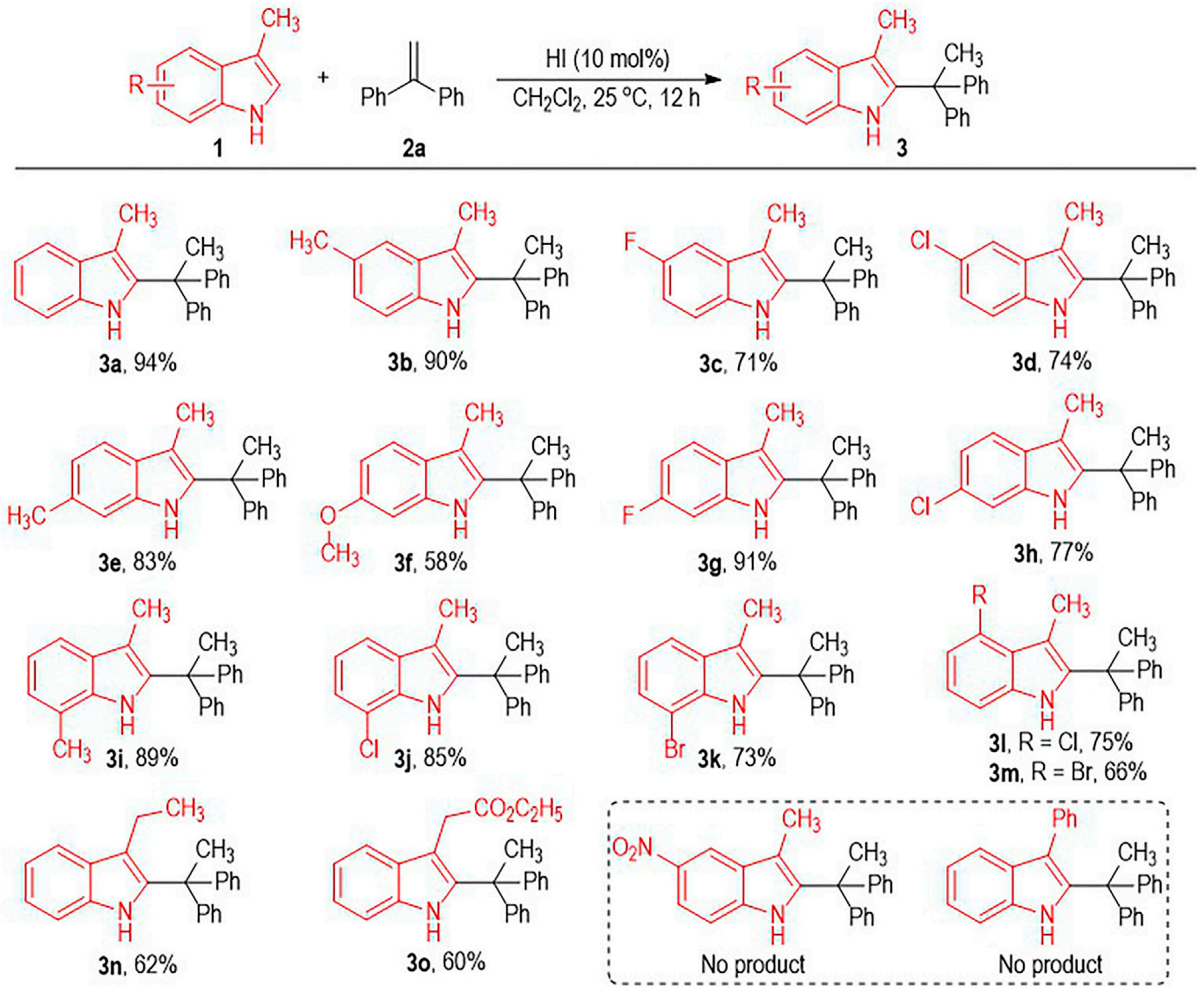
Scope of Indoles **1**.^a^. ^*a*^ Reaction conditions as stated in Table 1, entry 15. Yields are isolated yields after purification by column chromatography.

Having identified the optimal reaction condition for the HI-induced indole C2-alkylation, we next evaluated the scope of this transformation using indole substrates **1** bearing different substituents (Scheme 1). Both electron-donating and electron-withdrawing substituents are well tolerated on the benzene rings of the indole structure, with the target C2-alkylated indole products afforded in moderate to excellent yields (Scheme 1, **3a** to **3m**). We also examined the effect of the substitution position of the C5 with NO_2_ group, but gave no desired product. The C3-methyl group on the indole molecule can be changed into other alkyl groups, with the desired C2-alkylated indole products afforded in a bit lower yields (e.g., **3n** to **3o**). However, switching the C3-methyl group on the reaction substrates into a C3-phenyl group resulted in no formation of the target product.

**SCHEME 2:**
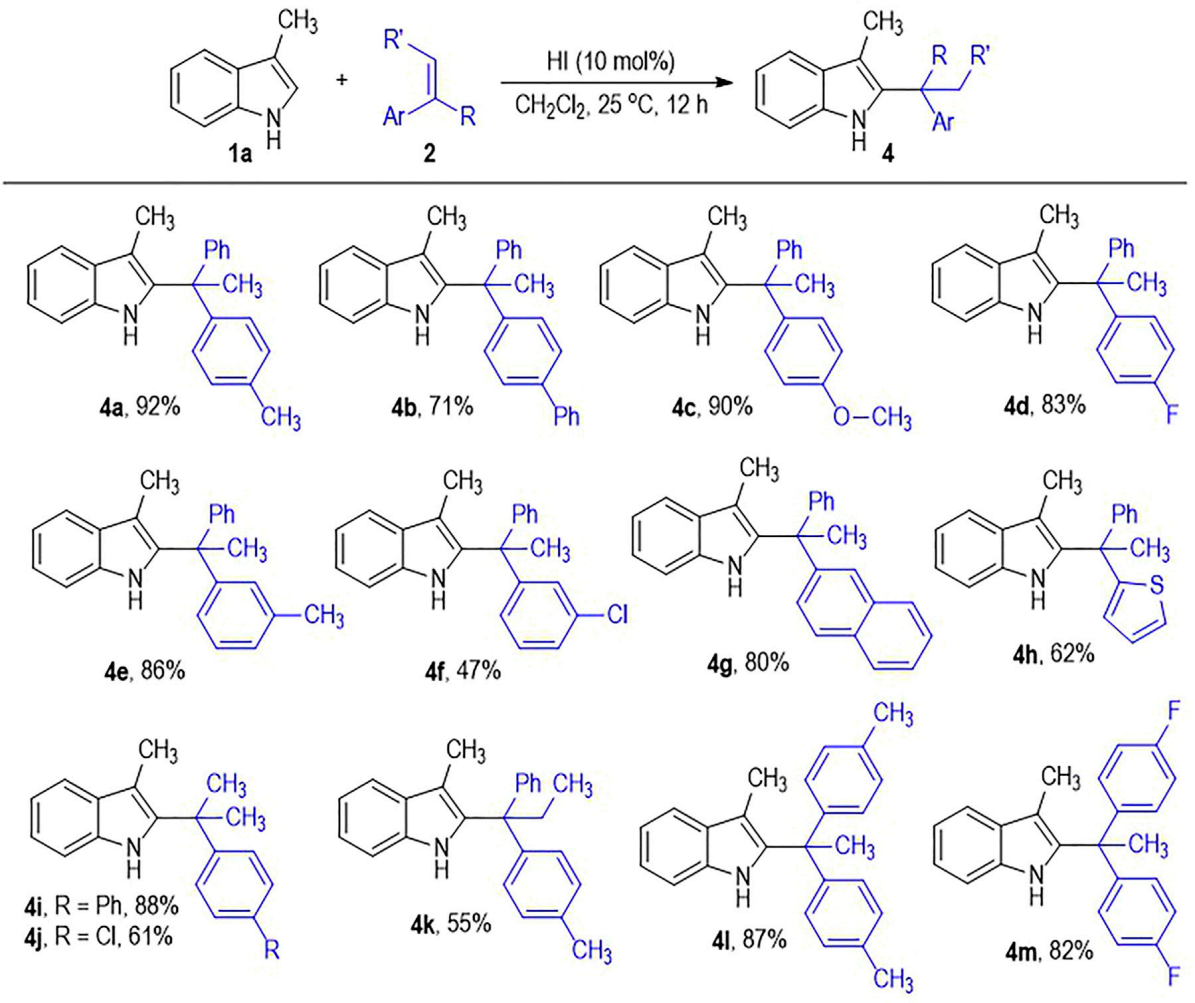
Scope of Alkenes **2**.^a^. ^*a*^ Reaction conditions as stated in Table 1, entry 15. Yields are isolated yields after purification by column chromatography.

The alkene substrates **2** can also tolerate diverse substituents and substitution patterns (Scheme 2). Various substituents can be introduced to the *para*-and *meta*-positions of the phenyl rings on **2a**, with the corresponding products afforded in good to excellent yields (Scheme 2, **4a** to **4f**). However, installing substituents on the *ortho*-position of the phenyl rings on **2a** leads to no formation of the desired products, which is probably due to the substantially increased steric hindrance during the C2-branched product formation process. One of the phenyl rings on **2a** can be switched into a naphthyl or a thiophenyl group with retention of the good to excellent product yields (**4g** to **4h**). To our delight, one of the two aryl groups on the alkene substrates can be replaced by a methyl group without much erosion on the product yield (**4i** to **4j**). It is worth noting that the internal alkene of 1,1-diphenylpropene also works well in this photo-induced indole C2-alkylation process, with the target product **4k** afforded in 55% yield. It is not surprising that the 1,1-diarylethenes bearing two substituted phenyl groups generally give the desired indole C2-branched alkylation products in excellent yields (**4l** to **4m**).

**SCHEME 3:**
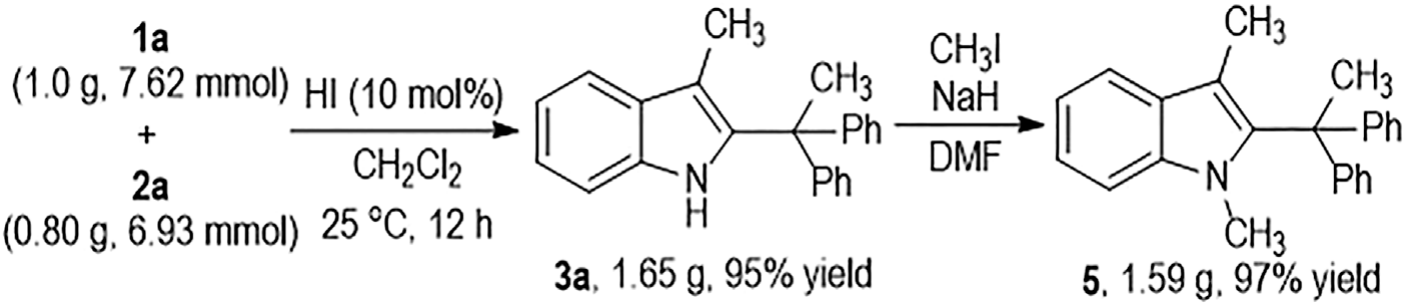
Gram-Scale Synthesis of **3a** and Its Synthetic Transformation.

The HI-induced C2-alkylation reaction of the 3-methylindole **1a** with **2a** can be carried out at Gram scales to give the functionalized indole product **3a** in an excellent yield (Scheme 3). The indole NH group on **3a** can be efficiently protected by CH_3_I and the *N*-methylindole product **5** can be afforded in almost quantitative yield.

## Conclusion

In summary, we have disclosed a metal-free reaction for the synthesis of 2-alkylation of 3-alkylindole derivatives. 1,1-Disubstituted alkenes are used as the alkylation reagent with the C2-branched alkylated indole products afforded in generally good to excellent yields with excellent Markovnikov regioselectivity. A catalytic amount of the commercially available and inexpensive HI is used as the sole reaction catalyst. Further investigations towards the applications of the C2-functionalized indole molecules are in progress in our laboratory.

## Data Availability

The original contributions presented in the study are included in the article/Supplementary Material, further inquiries can be directed to the corresponding author.
