# Supplementary material for: 2-Alkylation of 3-Alkyindoles With Unactivated Alkenes
Source: Front Chem. 2022 Feb 24;10:860764. doi: 10.3389/fchem.2022.860764 (PMC8907451; doi:10.3389/fchem.2022.860764)
Supplement: Supplementary file 1 [file DataSheet2.PDF]

Supporting Information

2-Alkylation of 3-Alkyindoles with Unactivated Alkenes

Xuling Pan,* Qian Liu and Yingling Nong

Key Laboratory Breeding Base of Green Pesticide and Agricultural Bioengineering, key Laboratory of Green Pesticide and Agricultural Bioengineering, Ministry of Education, Guizhou University, Huaxi District, Guiyang 550025, People's Republic of China.

E-mail: PanXL1996@163.com

Table of Contents

I. General information S2

II. Experimental section S3

III. General procedure for catalytic reactions and gram scale synthesis S7

IV. Stereochemistry determination *via* x-ray crystallographic analysis: S9

V. Characterization of substrates and products S10

VI. ^{1}H NMR, ^{13}C NMR and ^{19}F NMR spectra S20

VII. References. S53

I. General information

Commercially available materials purchased from J&K, Energy Chemical and Bide were used as received. The solvents was distilled over sodium and used directly. Unless otherwise specified, all reactions were carried out under an atmosphere of air in 4.0 mL vial. Glassware was dried for 2 hours at 120 °C and cooled down in vacuo. Proton nuclear magnetic resonance (^{1}H NMR) spectra were recorded on a Bruker (AVANCE III HD 400 MHz) spectrometer or on a JEOL-ECX-500 (500 MHz) spectrometer. Chemical shifts were recorded in parts per million (ppm, δ) relative to tetramethylsilane (δ 0.00) or chloroform (δ = 7.26, singlet). ^{1}H NMR splitting patterns are designated as singlet (s), doublet (d), triplet (t), quartet (q), dd (doublet of doublets); m (multiplets), and etc. All first-order splitting patterns were assigned on the basis of the appearance of the multiplet. Splitting patterns that could not be easily interpreted are designated as multiplet (m) or broad (br). Carbon nuclear magnetic resonance (^{13}C NMR) spectra were recorded on a Bruker (AVANCE III HD 101 MHz) spectrometer. Fluorine (^{19}F) nuclear magnetic resonance (^{19}F NMR) spectra were recorded on a Bruker (AVANCE III HD 376 MHz) spectrometer. The melting points (m.p.) of the title compounds were determined when left untouched on an XT-4-MP apparatus from Beijing Tech. Instrument Co. (Beijing, China). High resolution mass spectral analysis (HRMS) was performed on a quadrupole/electrostatic field orbitrap mass spectrometer. Relative configuration of the products was determined by X-ray crystallography. Analytical thin-layer chromatography (TLC) was carried out pre-coated silica gel plate (0.2 mm thickness). Visualization was performed using a UV lamp.

II. Experimental section

Part.1 General procedure for the synthesis substituted indoles.

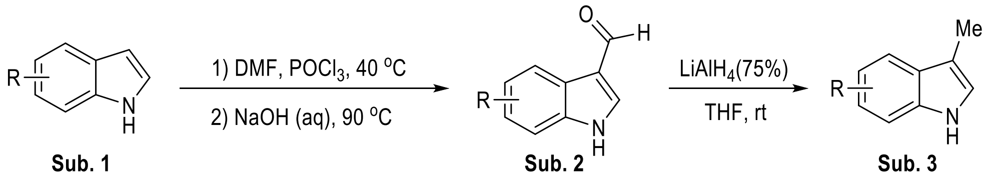

General procedure A for the synthesis of 3-alkylindoles: A three-necked flask was charged with indole (**sub. 1**, 10.00 mmol) and DMF (50.00 mmol). The mixture was stirred and cooled to 0 °C, then the freshly distilled $POCl_3$ (12.00 mmol) was added dropwise in 30 min. The solution was then heated at 40 °C for 2 h. Then 2M NaOH aqueous solution (60.0 mL) was added slowly and the reaction mixture was heated at 90 °C for another 1 h. AcOEt (30.0 mL) was added to dissolve the red solid, and the aqueous layer was extracted with AcOEt (2 × 30.0 mL). The combined organic phase was washed with brine, dried over Na_2SO_4, separated, and evaporated under reduced pressure. The corresponding 1*H*-indole-3-carbaldehyde (**Sub. 2**) was used in the next step without further purification.

In a dry Shlenk tube, $LiAlH_4$ (15.00 mmol) in THF (40.0 mL) was cooled at 0 °C, a solution of the above aldehyde **(Sub. 2)** in THF (40.0 mL) was added n dropwise in 15 min. The mixture was stirred overnight at room temperature. After the reaction was complete, H_2O (1.0 mL) was added carefully followed by NaOH (1.50 g) and H_2O (3.0 mL). The mixture was stirred for 15 min, then the solid was removed by filtration and was washed with diethyl ether (3 × 20.0 mL). The combined organic phase was dried over Na_2SO_4, filtrated, and evaporated under reduced pressure. The residue was purified by flash chromatography with petroleum ether / ethyl acetate (10 : 1) to afford the corresponding 3-methyl-1*H*-indole (**Sub. 3**).[1-4]

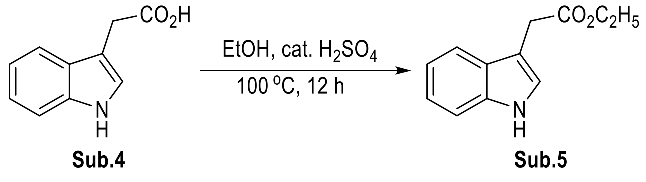

General procedure B for the synthesis of ethyl 2-(1*H*-indol-3-yl)acetate (S15):

In a 100.0 mL tube, a mixture of 2-(1*H*-indol-3-yl)acetic acid (**sub. 4**, 876.0 mg, 5.00 mmol), EtOH (10.0 mL) and catalytic amount of H_2SO_4 was refluxed. After 12 h, the reaction mixture was cooled to room temperature and neutralized with aqueous Na_2CO_3. The whole mixture was then extracted three times with dichloromethane (3 × 25.0 mL) and washed with water. Dichloromethane layer was dried over Na_2SO_4 and evaporated under reduced pressure. The crude mass was then purified by a short pad of silica using ethylacetate as eluent to afford 902.0 mg (89%) of the ethyl 2-(1*H*-indole3-yl)acetate (**Sub. 5**) as oil. Spectral data are in accordance with the reported data.[5]

Compounds **S1**,[1] **S2**,[1] **S3**,[1] **S4**,[1], **S5**,[1], **S6**,[2] **S7**,[1] **S8**,[1] **S9**,[2] **S10**,[3] **S11**,[3] **S12**,[2] **S13**,[3] **S14**,[4] were known in the literature.

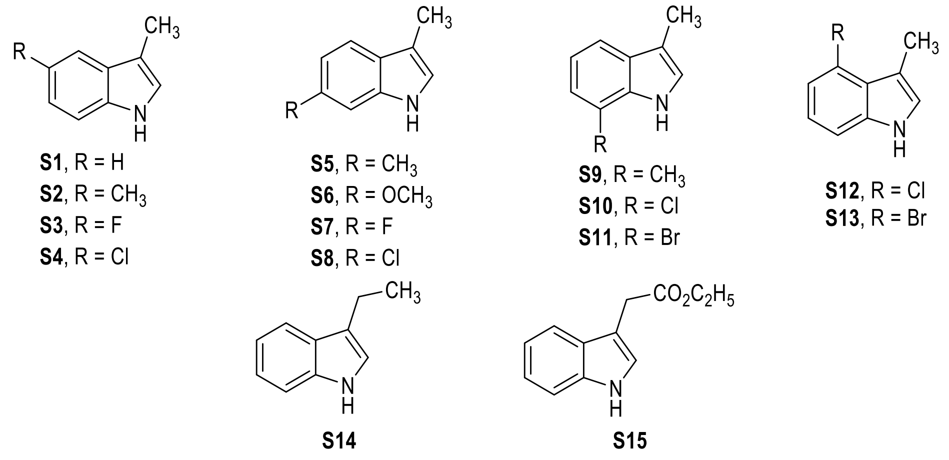

Part.2 General procedure for the synthesis substituted alkenes.

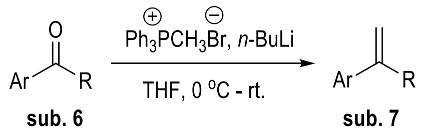

General procedure A for the synthesis of alkenes: To a suspension of methyltriphenylphosphonium bromide (15.00 mmol, 1.5 equiv.) in THF (30.0 mL) at 0 °C was added *n*-BuLi (15.00 mmol, 1.5 equiv.). The reaction was warmed to ambient temperature and stirred for 0.5 h. The ketone (10.00 mmol, 1.0 equiv.) was added. The resulting mixture was stirred at 50 °C for 2 h, cooled to rt. Saturated ammonium chloride (20.0 mL) was added to quench the reaction. The resulting

mixture was extracted with Et_2O (2 × 50.0 mL). The combined organic solution was dried over $MgSO_4$, filtered, and concentrated under reduced pressure. The residue was chromatographed through silica gel eluting with petroleum ether / ethyl acetate (95 : 5) to afford the title alkene (85-99% yield). [6-10]

Compounds **S16**,[6] **S17**,[7] **S18**,[10] **S19**,[6] **S20**,[6] **S21**, [6] **S22**,[6] **S23**,[9] **S24**,[8] **S25**,[8] **S26**,[10] **S27**,[6] **S28**,[6] were known in the literature.

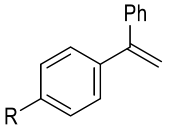

S16, R = Me
S17, R = Ph
S18, R = OCH_3
S19, R = F

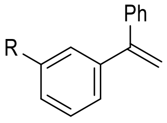

S20, R = CH_3
S21, R = Cl

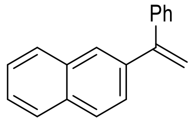

S22

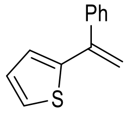

S23

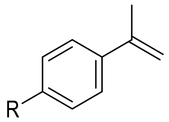

S24, R = Ph
S25, R = Cl

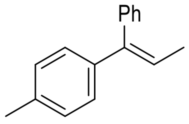

S26

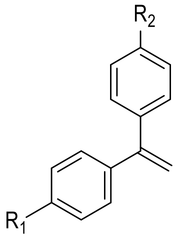

S27, $R_1 = R_2 = CH_3$
S28, $R_1 = R_2$ = F

II-2. Condition optimization for the synthesis of 3a.[a]

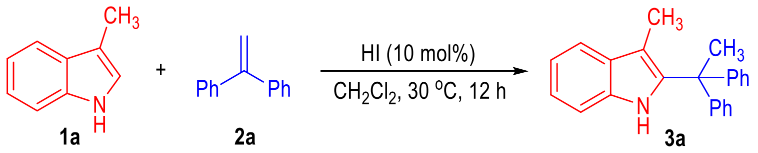

Entry	Solvents	Acid	Eq of acid	Temperature(°C)	Yield (%)[b]
1	1,4-dioxane	HI	0.3	30	Trace
2	EA	HI	0.3	30	80
3	THF	HI	0.3	30	NR
4	DCM	HI	0.3	30	92
5	Hexane	HI	0.3	30	73
6	CH_3CN	HI	0.3	30	85
7	MeOH	HI	0.3	30	NR
8	TBME	HI	0.3	30	NR
9	Toluene	HI	0.3	30	88
10	DMF	HI	0.3	30	NR
11	H_2O	HI	0.3	30	NR
12	DCM:H_2O 10:1	HI	0.3	30	93
13	DCM:H_2O 5:1	HI	0.3	30	NR
14	DCM:H_2O 3:1	HI	0.3	30	NR
15	CH_2Cl_2	HCl	0.3	30	21
16	CH_2Cl_2	HBr	0.3	30	87
17	CH_2Cl_2	CH_3CO_2H	0.3	30	NR
18	CH_2Cl_2	TFA	0.3	30	27
19	CH_2Cl_2	$H_2C_2O_4$	0.3	30	NR
20	CH_2Cl_2	TsOH	0.3	30	74
21	CH_2Cl_2	H_2SO_4	0.3	30	30
22	CH_2Cl_2	H_3PO_4	0.3	30	NR
23	CH_2Cl_2	$B(OH)_3$	0.3	30	NR
24	CH_2Cl_2	PhCOOH	0.3	30	NR
25	CH_2Cl_2	HI	0.2	30	94
26	CH_2Cl_2	HI	0.15	30	93
27	CH_2Cl_2	HI	0.1	30	93
28	CH_2Cl_2	HI	0.05	30	67
29	CH_2Cl_2	HI	0.03	30	58
30	CH_2Cl_2	HI	0.01	30	39
31	CH_2Cl_2	HI	0.1	25	94
32	CH_2Cl_2	HI	0.1	20	92
33	CH_2Cl_2	HI	0.1	10	92
34	CH_2Cl_2	HI	0.1	0	90
35	CH_2Cl_2	HI	0.1	-10	89
36	CH_2Cl_2	HI	0.1	-20	90
37	CH_2Cl_2	HI	0.1	-30	90
38[b]	CH_2Cl_2	HI	0.05	25	73
39[c]	CH_2Cl_2	HI	0.05	25	74

[a] Reaction conditions: unless otherwise stated, the reaction of 3-methyl-1*H*-indole **1a** (0.11 mmol), ethene-1,1-diyldibenzene **2a** (0.10 mmol) and HI (0.01 mmol) was carried out at 30 °C in CH_2Cl_2 (1.0 mL) for 12 h. [b] Reaction for 24 h; [c] Reaction for 48 h.

III. General procedure for catalytic reactions and gram scale synthesis

General procedure for the synthesis of product 3a

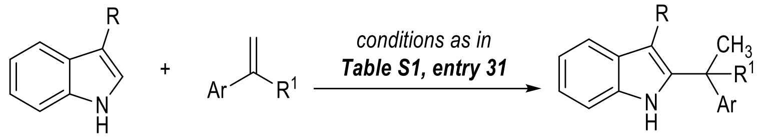

1a, 0.11 mmol **2a,** 0.10 mmol **3a**

To a 4.0 mL vial equipped with a magnetic stir bar was added 3-methylindole **1a** (0.11 mmol, 1.1 equiv, 14.4 mg), then added CH_2Cl_2 (1.0 mL) *via* syringe, and then 1,1-diphenylethene **2a** (0.10 mmol, 1.0 equiv, 18.0 mg) and hydriodic acid which was 55% aqueous solution (0.01 mmol, 1.96 mmL) were added. The reaction mixture was allowed to stirred for 12 hours at 25 ^{o}C. After completion of the reaction, monitored by TLC plate, the mixture was concentrated under reduced pressure. The resulting crude residue was purified *via* column chromatography on silica gel (petroleum ether / ethyl acetate = 50 : 1) to afford the desired products **3a** (94% yield).

Gram scale synthesis of product 3a

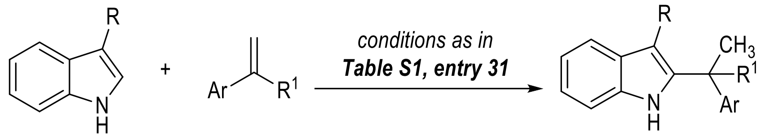

1a, 7.62 mmol **2a,** 6.93 mmol **3a**

To a 25.0 mL tube equipped with a magnetic stir bar was added 3-methylindole **1a** (7.62 mmol, 1.1 equiv, 0.80 g), then added CH_2Cl_2 (10.0 mL) *via* syringe, and then 1,1-diphenylethene **2a** (6.93 mmol, 1.0 equiv, 1.00 g) and hydriodic acid which was 55% aqueous solution (0.1 equiv., 8.2 mL) were added. The reaction mixture was allowed to stirred for 12 hours at 25 ^{o}C. After completion of the reaction, monitored by TLC plate, the mixture was concentrated under reduced pressure. The resulting crude residue was purified *via* column chromatography on silica gel (petroleum ether / ethyl acetate = 50 : 1) to afford the desired products **3a** (1.63 g, 95% yield).

Transformation of product 3a

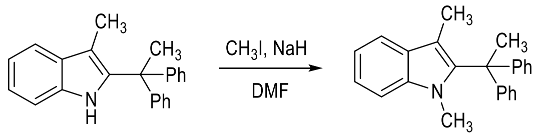

3a, 1.65 g, 95% yield **5**, 1.59 g, 97% yield

Add **3a** (1.65 g, 5.30 mmol, 1.0 equiv.) in DMF (20.0 mL) dropwise at 0 °C to a stirring solution of 60 % NaH (423.8 mg, 10.60 mmol, 2.0 equiv.) in dry DMF (10.0 mL). Allow the mixture to warm to room temperature and stir for 30 min. After cooling to 0 °C, add a solution of MeI (1.50 g, 10.60 mmol, 2.0 equiv.) in DMF (5.0 mL) dropewise. Stir the resulting mixture at room temperature overnight. Quench the reaction with water and extract three times with ethyl acetate. Dry the combine organic phases over $MgSO_4$, filter and concentrate under vacuum. The resulting crude residue was purified *via* column chromatography on silica gel to afford the desired product **5** (1.59 g, 97% yield).

IV. Stereochemistry determination *via* x-ray crystallographic analysis:

A colorless block crystal of **3a** was obtained by vaporization of its MeOH / CH_2Cl_2 / petroleum ether solution.

CCDC **2121520** contain the supplementary X-ray crystallographic data of **3a**. These data can be obtained free of charge from The Cambridge Crystallographic Data Centre *via* www.ccdc.cam.ac.uk/data request/cif.

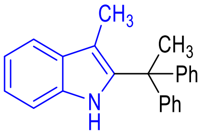

3a

≡

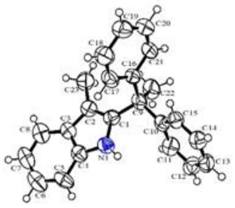

V. Characterization of products

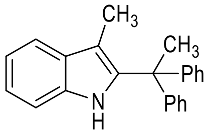

3a

2-(1,1-diphenylethyl)-3-methyl-1*H*-indole (3a):

White solid, 94% yield, 29.3 mg, m.p. 115-117 °C.

^{1}H NMR (400 MHz, CDCl$_3$) δ 7.54 – 7.50 (m, 1H), 7.33 – 7.26 (m, 6H), 7.20 – 7.14 (m, 5H), 7.11 – 7.06 (m, 2H), 2.27 (s, 3H), 1.98 (s, 3H).

^{13}C NMR (101 MHz, CDCl$_3$) δ 146.9, 139.6, 133.9, 130.3, 128.4, 128.3, 126.7, 121.4, 119.2, 118.2, 110.5, 108.0, 49.5, 29.1, 10.3.

HRMS (ESI, m/z): Mass calcd. for $C_{23}H_{21}N$ [M+H], 312.1747; found 312.1737.

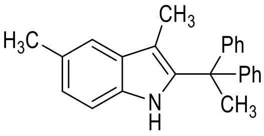

3b

2-(1,1-diphenylethyl)-3,5-dimethyl-1*H*-indole (3b):

White solid, 90% yield, 28.0 mg, m.p. 154-156 °C.

^{1}H NMR (400 MHz, CDCl$_3$) δ 7.32 – 7.28 (m, 5H), 7.25 – 7.22 (m, 2H), 7.19 – 7.16 (m, 4H), 7.06 (d, *J* = 8.21 Hz, 1H), 6.93 (dd, *J* = 8.26, 1.66 Hz, 1H), 2.45 (s, 3H), 2.27 (s, 3H), 1.95 (s, 3H).

^{13}C NMR (101 MHz, CDCl$_3$) δ 147.0, 139.8, 132.2, 130.5, 128.4, 128.3, 128.3, 126.6, 122.9, 117.9, 110.2, 107.5, 49.5, 29.1, 21.6, 10.3.

HRMS (ESI, m/z): Mass calcd. for $C_{24}H_{23}N$ [M-H], 324.1758; found 324.1752.

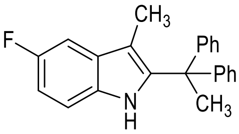

3c

2-(1,1-diphenylethyl)-5-fluoro-3-methyl-1*H*-indole (3c):

White solid, 71% yield, 23.4 mg. m.p. 131-133 °C.

^{1}H NMR (400 MHz, CDCl$_3$) δ 7.34 – 7.27 (m, 7H), 7.19 – 7.16 (m, 4H), 7.08 – 7.05 (m, 1H), 6.87 – 6.79 (m, 1H), 2.27 (s, 3H), 1.94 (s, 3H).

^{13}C NMR (101 MHz, CDCl$_3$) δ 157.8 (d, *J* = 234.1 Hz), 146.6, 141.6, 130.7 (d, *J* = 9.4 Hz), 130.3, 128.4, 128.1, 126.7, 111.0 (d, *J* = 9.6 Hz), 109.4 (d, *J* = 26.4 Hz), 108.2 (d, *J* = 4.7 Hz), 103.0 (d, *J* = 23.2 Hz), 49.5, 28.8,10.3.

^{19}F NMR (377 MHz, CDCl$_3$) δ -125.01.

HRMS (ESI, m/z): Mass calcd. for $C_{23}H_{20}FN$ [M-H], 328.1507; found 328.1513.

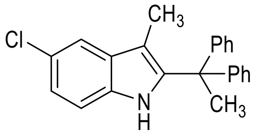
3d

5-chloro-2-(1,1-diphenylethyl)-3-methyl-1*H*-indole (3d): White solid, 74% yield, 25.6 mg. m.p. 138-140 °C.

^{1}H NMR (400 MHz, CDCl$_3$) δ 7.48 – 7.47 (m, 1H), 7.33 – 7.27 (m, 6H), 7.18 – 7.15 (m, 4H), 7.09 – 7.03 (m, 2H), 2.27 (s, 3H), 1.94 (s, 3H).

^{13}C NMR (101 MHz, CDCl$_3$) δ 146.6, 141.2, 132.2, 131.4, 128.4, 128.1, 126.8, 124.9, 121.5, 117.7, 111.5, 107.8, 49.5, 28.9, 10.2.

HRMS (ESI, m/z): Mass calcd. for $C_{23}H_{20}ClN$ [M-H], 344.1212; found 344.1216.

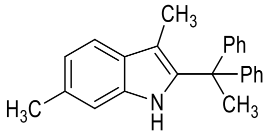
3e

2-(1,1-diphenylethyl)-3,6-dimethyl-1*H*-indole (3e): White solid, 83% yield, 27.1 mg. m.p. 50-51 °C.

^{1}H NMR (400 MHz, CDCl$_3$) δ 7.40 (d, *J* = 8.11 Hz, 1H), 7.35 – 7.25 (m, 5H), 7.25 – 7.23 (m, 1H), 7.22 (s, 1H), 7.18 – 7.16 (m, 4H), 6.96 – 6.91 (m, 2H), 2.41 (s, 3H), 2.26 (s, 3H), 1.95 (s, 3H).

^{13}C NMR (101 MHz, CDCl$_3$) δ 147.1, 138.8, 134.3, 131.1, 128.3, 128.3, 128.3, 126.6, 120.9, 117.9, 110.5, 107.9, 49.5, 29.1, 21.7, 10.3.

HRMS (ESI, m/z): Mass calcd. for $C_{24}H_{23}N$ [M-H], 324.1758; found 324.1755.

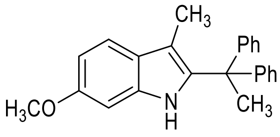
3f

2-(1,1-diphenylethyl)-6-methoxy-3-methyl-1*H*-indole (3f): White solid, 58% yield, 19.8 mg. m.p. 92-94 °C.

^{1}H NMR (400 MHz, CDCl$_3$) δ 7.39 (d, *J* = 8.60 Hz, 1H), 7.33 – 7.27 (m, 5H), 7.22 (s, 1H), 7.20 – 7.16 (m, 4H), 6.77 (dd, *J* = 8.62, 2.32 Hz, 1H), 6.69 (d, *J* = 2.24 Hz, 1H), 3.80 (s, 3H), 2.27 (s, 3H), 1.95 (s, 3H).

^{13}C NMR (101 MHz, CDCl$_3$) δ 156.1, 147.1, 138.1, 134.4, 128.3, 128.2, 126.5, 124.6, 118.8, 109.0, 107.8, 94.1, 55.7, 49.4, 29.1, 10.3.

HRMS (ESI, m/z): Mass calcd. for $C_{24}H_{23}NO$ [M-H], 340.1707; found 340.1708.

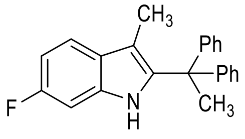

3g

2-(1,1-diphenylethyl)-6-fluoro-3-methyl-1*H*-indole (3g): White solid, 91% yield, 29.9 mg. m.p. 121-123 °C.

^{1}H NMR (400 MHz, $CDCl_3$) δ 7.4 – 7.4 (m, 1H), 7.3 – 7.3 (m, 6H), 7.2 – 7.1 (m, 4H), 6.9 – 6.8 (m, 2H), 2.3 (s, 3H), 2.0 (s, 3H).

^{13}C NMR (101 MHz, $CDCl_3$) δ 159.6 (d, J = 236.5 Hz), 146.7, 139.7, 139.7, 133.6 (d, J = 12.4 Hz), 128.4, 128.1, 126.8, 126.7, 118.8 (d, J = 10.1 Hz), 107.7 (d, J = 24.3 Hz), 96.8 (d, J = 26.1 Hz), 49.5, 29.0, 10.2.

^{19}F NMR (376 MHz, $CDCl_3$) δ -122.10.

HRMS (ESI, m/z): Mass calcd. for $C_{23}H_{20}FN$ [M-H], 328.1507; found 328.1509.

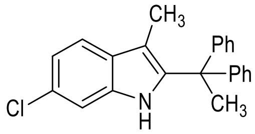

3h

6-chloro-2-(1,1-diphenylethyl)-3-methyl-1*H*-indole (3h): White solid, 77% yield, 26.5 mg. m.p. 71-73 °C.

^{1}H NMR (400 MHz, $CDCl_3$) δ 7.41 (d, J = 8.41 Hz, 1H), 7.33 – 7.28 (m, 6H), 7.17 – 7.14 (m, 5H), 7.05 (dd, J = 8.42, 1.86 Hz, 1H), 2.27 (s, 3H), 1.95 (s, 3H).

^{13}C NMR (101 MHz, $CDCl_3$) δ 146.6, 140.3, 134.1, 128.9, 128.4, 128.1, 127.1, 126.8, 119.8, 119.1, 110.4, 108.2, 49.5, 28.9, 10.2.

HRMS (ESI, m/z): Mass calcd. for $C_{23}H_{20}ClN$ [M-H], 344.1212; found 344.1215.

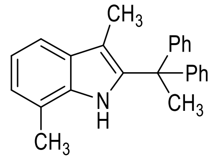

3i

2-(1,1-diphenylethyl)-3,7-dimethyl-1*H*-indole (3i): White solid, 89% yield, 29.0 mg. m.p. 121-123 °C.

^{1}H NMR (400 MHz, $CDCl_3$) δ 7.38 (d, J = 7.89 Hz, 1H), 7.33 – 7.27 (m, 5H), 7.24 – 7.23 (m, 1H), 7.21 – 7.19 (m, 4H), 7.01 (t, J = 7.72 Hz, 1H), 6.93 (d, 1H), 2.29 (s, 3H), 2.28 (s, 3H), 1.94 (s, 3H).

^{13}C NMR (101 MHz, $CDCl_3$) δ 146.9, 139.1, 133.5, 129.8, 128.3, 128.2, 126.6, 122.0, 119.6, 119.4, 116.0, 108.7, 49.6, 29.2, 16.3, 10.4.

HRMS (ESI, m/z): Mass calcd. for $C_{24}H_{23}N$ [M-H], 324.1758; found 324.1768.

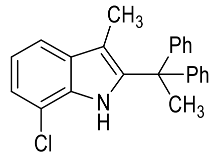

3j

7-chloro-2-(1,1-diphenylethyl)-3-methyl-1*H*-indole (3j):

White solid, 85% yield, 29.3 mg. m.p. 108-110 °C.

^{1}H NMR (400 MHz, $CDCl_3$) δ 7.55 (s, 1H), 7.41 (d, *J* = 7.85 Hz, 1H), 7.34 – 7.27 (m, 6H), 7.20 – 7.17 (m, 4H), 7.11 – 7.09 (m, 1H), 7.03 – 7.00 (m, 1H), 2.29 (s, 3H), 1.92 (s, 3H).

^{13}C NMR (101 MHz, $CDCl_3$) δ 146.4, 140.4, 131.6, 131.1, 128.4, 128.1, 126.8, 120.7, 119.9, 116.8, 115.9, 109.3, 49.5, 29.1, 10.4.

HRMS (ESI, m/z): Mass calcd. for $C_{23}H_{20}ClN$ [M-H], 344.1212; found 344.1213.

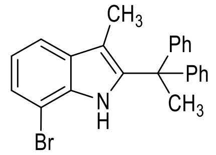

3k

7-bromo-2-(1,1-diphenylethyl)-3-methyl-1*H*-indole (3k):

White solid, 73% yield, 28.6 mg. m.p. 120-122 °C.

^{1}H NMR (600 MHz, $CDCl_3$) δ 7.52 (s, 1H), 7.45 (d, *J* = 7.03 Hz, 1H), 7.34 – 7.27 (m, 6H), 7.24 – 7.23 (m, 1H), 7.20 – 7.16 (m, 4H), 6.95 (t, *J* = 7.75 Hz, 1H), 2.29 (s, 3H), 1.91 (s, 3H).

^{13}C NMR (101 MHz, $CDCl_3$) δ 146.4, 140.4, 132.6, 131.3, 128.4, 128.1, 126.8, 123.6, 120.2, 117.4, 109.5, 104.1, 49.5, 29.0, 10.4.

HRMS (ESI, m/z): Mass calcd. for $C_{23}H_{20}BrN$ [M-H], 388.0706; found 388.0708.

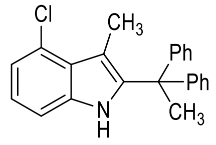

3l

4-chloro-2-(1,1-diphenylethyl)-3-methyl-1*H*-indole (3l):

White solid, 75% yield, 26.1 mg. m.p. 179-181 °C.

^{1}H NMR (400 MHz, $CDCl_3$) δ 7.34 – 7.32 (m, 3H), 7.31 – 7.28 (m, 3H), 7.21 – 7.19 (m, 4H), 7.03 – 7.00 (m, 2H), 6.94 (t, *J* = 7.73 Hz, 1H), 2.27 (s, 3H), 2.25 (s, 3H).

^{13}C NMR (101 MHz, $CDCl_3$) δ 146.4, 141.0, 135.3, 128.5, 128.2, 126.8, 126.5, 126.3, 121.6, 120.6, 109.4, 108.6, 49.4, 28.6, 12.6.

HRMS (ESI, m/z): Mass calcd. for $C_{23}H_{20}ClN$ [M-H], 344.1212; found 344.1221.

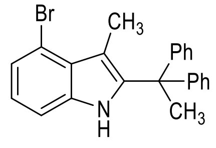
3m

4-bromo-2-(1,1-diphenylethyl)-3-methyl-1*H*-indole (3m):

White solid, 66% yield, 25.8 mg. m.p. 196-198 °C.

^{1}H NMR (400 MHz, $CDCl_3$) δ 7.35 – 7.32 (m, 3H), 7.31 – 7.26 (m, 4H), 7.22 – 7.19 (m, 4H), 7.09 – 7.07 (m, 1H), 6.87 (t, *J* = 7.86 Hz, 1H), 2.27 (s, 3H), 2.25 (s, 3H).

^{13}C NMR (101 MHz, $CDCl_3$) δ 145.8, 140.7, 134.5, 127.9, 127.6, 127.0, 126.2, 123.5, 121.4, 113.5, 109.4, 108.4, 48.8, 28.0, 12.3.

HRMS (ESI, m/z): Mass calcd. for $C_{23}H_{20}BrN$ [M-H], 388.0706; found 388.0712.

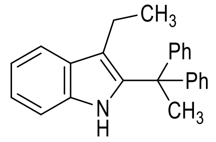
3n

2-(1,1-diphenylethyl)-3-ethyl-1*H*-indole (3n):

White solid, 62% yield, 20.2 mg. m.p.108-110 °C.

^{1}H NMR (400 MHz, $CDCl_3$) δ 7.58 – 7.55 (m, 1H), 7.32 – 7.26 (m, 6H), 7.20 – 7.14 (m, 5H), 7.13 – 7.06 (m, 2H), 2.47 (q, *J* = 7.51 Hz, 2H), 2.30 (s, 3H), 1.06 (t, *J* = 7.54 Hz, 3H).

^{13}C NMR (101 MHz, $CDCl_3$) δ 147.2, 139.1, 134.1, 129.4, 128.4, 128.3, 126.7, 121.3, 119.1, 118.7, 114.6, 110.6, 49.6, 29.7, 18.5, 14.7.

HRMS (ESI, m/z): Mass calcd. for $C_{24}H_{23}N$ [M+Na], 348.1723; found 348.1703.

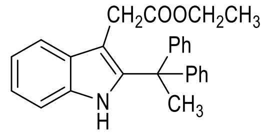
3o

ethyl 2-(2-(1,1-diphenylethyl)-1*H*-indol-3-yl)acetate (3o):

White solid, 60% yield, 23.1 mg. m.p.146-148 °C.

^{1}H NMR (400 MHz, $CDCl_3$) δ 7.55 – 7.51 (m, 1H), 7.47 (s, 1H), 7.32 – 7.27 (m, 5H), 7.25 – 7.23 (m, 1H), 7.21 – 7.16 (m, 5H), 7.14 – 7.07 (m, 2H), 4.03 (q, *J* = 7.13 Hz, 2H), 3.36 (s, 2H), 2.30 (s, 3H), 1.16 (t, *J* = 7.16 Hz, 3H).

^{13}C NMR (101 MHz, $CDCl_3$) δ 170.2, 145.0, 139.6, 132.4, 128.1, 126.8, 126.6, 125.2, 120.2, 118.1, 117.0, 109.0, 103.9, 58.9, 47.8, 29.7, 28.1, 12.6.

HRMS (ESI, m/z): Mass calcd. for $C_{26}H_{25}NO_2$ [M-H], 382.1813; found 382.1820.

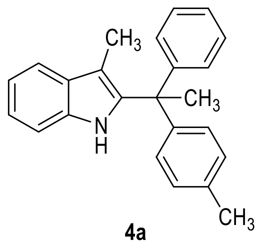

4a

3-methyl-2-(1-phenyl-1-(p-tolyl)ethyl)-1*H*-indole (4a):

White solid, 92% yield, 30.1 mg. m.p.109-111 °C.

^{1}H NMR (400 MHz, $CDCl_3$) δ 7.53 – 7.51 (m, 1H), 7.32 (s, 1H), 7.29 – 7.27 (m, 2H), 7.20 – 7.05 (m, 10H), 2.34 (s, 3H), 2.25 (s, 3H), 1.99 (s, 3H).

^{13}C NMR (101 MHz, $CDCl_3$) δ 147.2, 144.0, 139.8, 136.3, 133.9, 130.3, 129.1, 128.3, 128.2, 128.2, 126.6, 121.3, 119.1, 118.2, 110.5, 107.9, 49.2, 29.1, 21.0, 10.4.

HRMS (ESI, m/z): Mass calcd. for $C_{24}H_{23}N$ [M+Na], 348.1723; found 348.1707.

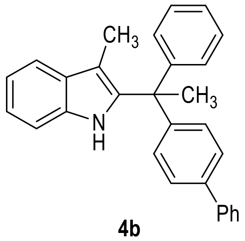

4b

2-(1-([1,1'-biphenyl]-4-yl)-1-phenylethyl)-3-methyl-1*H*-indole (4b):

White solid, 71% yield, 27.6 mg. m.p.101-103 °C.

^{1}H NMR (400 MHz, $CDCl_3$) δ 7.61 – 7.59 (m, 2H), 7.55 – 7.53 (m, 3H), 7.44 – 7.40 (m, 2H), 7.38 (s, 1H), 7.35 – 7.28 (m, 4H), 7.25 – 7.20 (m, 4H), 7.19 – 7.16 (m, 1H), 7.13 – 7.08 (m, 2H), 2.30 (s, 3H), 2.02 (s, 3H).

^{13}C NMR (101 MHz, $CDCl_3$) ^{13}C NMR (101 MHz, $CDCl_3$) δ 146.8, 145.9, 140.5, 139.5, 139.3, 133.9, 130.2, 128.8, 128.6, 128.4, 128.2, 127.3, 127.0, 126.9, 126.7, 121.3, 119.1, 118.2, 110.5, 108.0, 49.3, 29.0, 10.3.

HRMS (ESI, m/z): Mass calcd. for $C_{25}H_{25}N$ [M-H], 387.1987; found 387.1941.

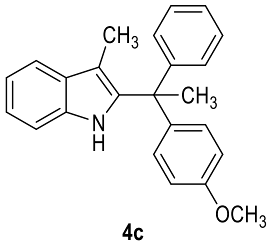

4c

2-(1-(4-methoxyphenyl)-1-phenylethyl)-3-methyl-1*H*-indole (4c):

White solid, 90% yield, 30.9 mg. m.p.120-122 °C.

^{1}H NMR (400 MHz, $CDCl_3$) δ 7.54 – 7.51 (m, 1H), 7.33 (s, 1H), 7.31 – 7.27 (m, 2H), 7.23 (s, 1H), 7.19 – 7.15 (m, 3H), 7.12 – 7.07 (m, 4H), 6.85 – 6.82 (m, 2H), 3.80 (s, 3H), 2.25 (s, 3H), 1.99 (s, 3H).

^{13}C NMR (101 MHz, $CDCl_3$) δ 156.5, 145.6, 138.3, 137.5, 132.2, 128.7, 127.7, 126.7, 126.6, 125.0, 119.7, 117.5, 116.6, 112.0, 108.9, 106.2, 53.7, 47.3, 27.5, 8.7.

HRMS (ESI, m/z): Mass calcd. for $C_{24}H_{23}NO$ [M+Na], 364.1672; found 364.1657.

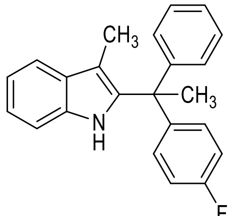

4d

2-(1-(4-fluorophenyl)-1-phenylethyl)-3-methyl-1*H*-indole (4d): White solid, 83% yield, 27.5 mg. m.p.51-53 °C.

^{1}H NMR (400 MHz, $CDCl_3$) δ 7.5 – 7.5 (m, 1H), 7.3 – 7.3 (m, 3H), 7.2 – 7.1 (m, 7H), 7.0 – 7.0 (m, 2H), 2.3 (s, 3H), 2.0 – 2.0 (m, 3H).

^{13}C NMR (101 MHz, $CDCl_3$) δ 161.5 (d, *J* = 245.94 Hz), 146.8, 142.7 (d, *J* = 3.42 Hz), 139.3, 133.9, 130.2, 129.8 (d, *J* = 7.99 Hz), 128.5, 128.2, 126.8, 121.5, 119.3, 118.3, 115.1 (d, *J* = 21.09 Hz), 110.5, 108.1, 49.0, 29.3, 10.3.

^{19}F NMR (377 MHz, $CDCl_3$) δ -105.97.

HRMS (ESI, m/z): Mass calcd. for $C_{23}H_{20}FN$ [M-H], 328.1507; found 328.1527.

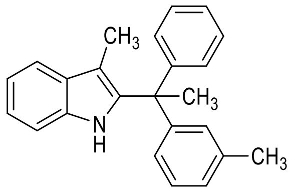

4e

3-methyl-2-(1-phenyl-1-(m-tolyl)ethyl)-1*H*-indole (4e): White solid, 86% yield, 28.1 mg. 71-73 °C.

^{1}H NMR (400 MHz, $CDCl_3$) δ 7.55 – 7.52 (m, 1H), 7.32 – 7.25 (m, 3H), 7.21 – 7.16 (m, 4H), 7.11 – 7.06 (m, 3H), 7.04 – 7.03 (m, 1H), 6.96 – 6.94 (m, 1H), 2.30 (s, 3H), 2.27 (s, 3H), 1.99 (s, 3H).

^{13}C NMR (101 MHz, $CDCl_3$) δ 147.0, 146.8, 139.7, 137.9, 133.8, 130.3, 128.8, 128.3, 128.2, 128.1, 127.4, 126.5, 125.4, 121.2, 119.0, 118.1, 110.4, 107.9, 49.4, 29.0, 21.7, 10.3.

HRMS (ESI, m/z): Mass calcd. for $C_{24}H_{23}N$ [M-H], 324.1758; found 324.1753.

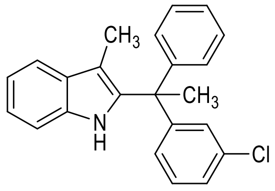

4f

2-(1-(3-chlorophenyl)-1-phenylethyl)-3-methyl-1*H*-indole (4f): White solid, 47% yield, 16.8 mg. m.p.121-123 °C.

^{1}H NMR (400 MHz, $CDCl_3$) δ 7.55 – 7.52 (m, 1H), 7.35 – 7.28 (m, 4H), 7.24 – 7.23 (m, 1H), 7.22 – 7.21 (m, 1H), 7.20 – 7.15 (m, 3H), 7.14 – 7.08 (m, 2H), 7.06 – 7.04 (m, 1H), 2.25 (s, 3H), 1.98 (s, 3H).

^{13}C NMR (101 MHz, $CDCl_3$) δ 149.1, 146.1, 138.7, 134.4, 133.9, 130.1, 129.5, 128.5, 128.3, 128.1, 126.9, 126.8, 126.6, 121.5, 119.2, 118.3, 110.5, 108.2, 49.4, 29.0,

10.3.

HRMS (ESI, m/z): Mass calcd. for $C_{23}H_{20}ClN$ [M-H], 344.1212; found 344.1217.

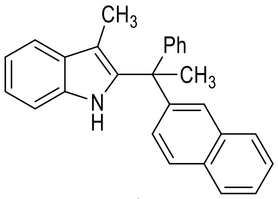

4g

3-methyl-2-(1-(naphthalen-1-yl)-1-phenylethyl)-1*H*-indole (4g):

White solid, 80% yield, 28.8 mg. m.p.54-56 °C.

^{1}H NMR (400 MHz, CDCl$_3$) δ 7.82 – 7.80 (m, 1H), 7.76 (d, *J* = 8.68 Hz, 1H), 7.72 – 7.70 (m, 1H), 7.59 (d, *J* = 1.93 Hz, 1H), 7.57 – 7.53 (m, 1H), 7.46 – 7.43 (m, 2H), 7.37 – 7.34 (m, 2H), 7.31 – 7.27 (m, 2H), 7.24 – 7.22 (m, 2H), 7.15 – 7.08 (m, 3H), 2.36 (s, 3H), 2.02 (s, 3H).

^{13}C NMR (101 MHz, CDCl$_3$) δ 146.7, 144.4, 139.4, 134.0, 133.2, 132.2, 130.4, 128.5, 128.4, 128.4, 128.1, 127.5, 127.0, 126.8, 126.6, 126.2, 126.1, 121.4, 119.2, 118.3, 110.6, 108.2, 49.7, 29.1, 10.5.

HRMS (ESI, m/z): Mass calcd. for $C_{27}H_{23}N$ [M-H], 360.1758; found 360.1761.

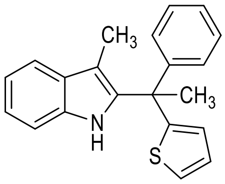

4h

3-methyl-2-(1-phenyl-1-(thiophen-2-yl)ethyl)-1*H*-indole (4h):

White solid, 62% yield, 19.6 mg. m.p.81-83 °C.

^{1}H NMR (400 MHz, CDCl$_3$) δ 7.53 – 7.51 (m, 1H), 7.32 – 7.23 (m, 6H), 7.21 – 7.19 (m, 1H), 7.14 – 7.07 (m, 2H), 6.97 (dd, *J* = 5.15, 3.55 Hz, 1H), 6.79 (dd, *J* = 3.53, 1.25 Hz, 1H), 2.30 (s, 3H), 1.97 (s, 3H).

^{13}C NMR (101 MHz, CDCl$_3$) δ 152.3, 146.6, 139.3, 133.8, 130.2, 128.3, 127.5, 126.9, 126.6, 126.0, 124.9, 121.5, 119.2, 118.3, 110.5, 107.7, 47.3, 29.8, 10.0.

HRMS (ESI, m/z): Mass calcd. for $C_{21}H_{19}NS$ [M-H], 316.1165; found 316.1174.

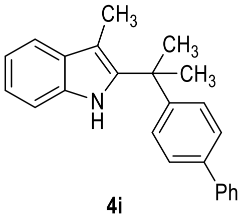

4i

2-(2-([1,1'-biphenyl]-4-yl)propan-2-yl)-3-methyl-1*H*-indole (4i):

White solid, 88% yield, 28.6 mg. 31-33 °C.

^{1}H NMR (400 MHz, CDCl$_3$) δ 7.80 (s, 1H), 7.59 – 7.57 (m, 2H), 7.53 – 7.49 (m, 3H), 7.43 – 7.39 (m, 2H), 7.37 – 7.32 (m, 3H), 7.31 – 7.26 (m, 1H), 7.15 – 7.09 (m, 2H), 2.06 (s, 3H), 1.81 (s, 6H).

^{13}C NMR (101 MHz, $CDCl_3$) δ 147.9, 141.0, 140.8, 139.0, 134.1, 130.4, 128.8, 127.3, 127.0, 127.0, 126.8, 121.2, 119.2, 118.1, 110.4, 106.9, 40.4, 29.7, 9.8.

HRMS (ESI, m/z): Mass calcd. for $C_{24}H_{23}N$ [M+Na], 348.1723; found 348.1711.

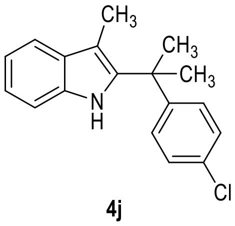

4j

2-(2-(4-chlorophenyl)propan-2-yl)-3-methyl-1*H*-indole (4j):

White solid, 61% yield, 17.3 mg. 77-79 ^{o}C.

^{1}H NMR (400 MHz, $CDCl_3$) δ 7.81 (s, 1H), 7.48 (dd, J = 6.21 Hz, 1H), 7.30 – 7.27 (m, 1H), 7.24 – 7.19 (m, 4H), 7.16 – 7.07 (m, 2H), 1.98 (s, 3H), 1.75 (s, 7H).

^{13}C NMR (101 MHz, $CDCl_3$) δ 147.4, 140.5, 134.1, 131.9, 130.3, 128.4, 127.7, 121.4, 119.2, 118.1, 110.4, 106.9, 40.2, 29.6, 9.7.

HRMS (ESI, m/z): Mass calcd. for $C_{18}H_{18}ClN$ [M-H], 282.1055; found 282.1063.

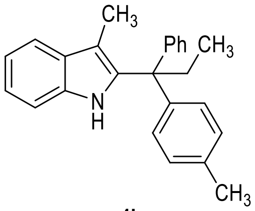

4k

2-(1,1-diphenylpropyl)-3-methyl-1*H*-indole (4k):

White solid, 55% yield, 17.9 mg. 121-123 ^{o}C.

^{1}H NMR (400 MHz, $CDCl_3$) δ 7.62 (s, 1H), 7.53 – 7.51 (m, 1H), 7.37 – 7.34 (m, 2H), 7.30 – 7.28 (m, 1H), 7.25 – 7.18 (m, 5H), 7.14 – 7.07 (m, 4H), 2.75 – 2.69 (m, 2H), 2.32 (s, 3H), 1.97 (s, 3H), 0.87 (t, J = 7.28 Hz, 3H).

^{13}C NMR (101 MHz, $CDCl_3$) δ 146.0, 142.6, 138.1, 135.9, 133.9, 130.2, 129.0, 128.4, 128.4, 128.2, 126.2, 121.1, 118.9, 118.1, 110.4, 108.4, 53.7, 31.8, 20.9, 10.7, 10.6.

HRMS (ESI, m/z): Mass calcd. for $C_{24}H_{23}N$ [M-H], 324.1758; found 324.1773.

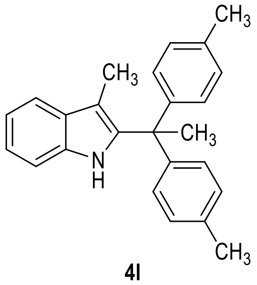

4l

2-(1,1-di-p-tolylethyl)-3-methyl-1*H*-indole (4l):

White solid, 87% yield, 29.7 mg. m.p.138-140 ^{o}C.

^{1}H NMR (400 MHz, $CDCl_3$) δ 7.55 – 7.50 (m, 1H), 7.32 (s, 1H), 7.18 – 7.14 (m, 1H), 7.11 – 7.05 (m, 10H), 2.34 (s, 6H), 2.23 (s, 3H), 2.00 (s, 3H).

^{13}C NMR (101 MHz, $CDCl_3$) δ 144.1, 140.0, 136.1, 133.8,

130.3, 129.0, 128.1, 121.2, 119.0, 118.1, 110.5, 107.7, 48.8, 29.0, 21.0, 10.3.

HRMS (ESI, m/z): Mass calcd. for $C_{25}H_{25}N$ [M+Na], 362.1879; found 362.1867.

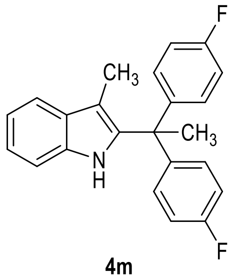

4m

2-(1,1-bis(4-fluorophenyl)ethyl)-3-methyl-1*H*-indole (4m): White solid, 82% yield, 28.6 mg. m.p.98-100 °C.

^{1}H NMR (400 MHz, CDCl$_3$) δ 7.53 – 7.51 (m, 1H), 7.30 (s, 1H), 7.20 – 7.17 (m, 1H), 7.15 – 7.08 (m, 6H), 7.03 – 6.96 (m, 4H), 2.24 (d, *J* = 1.86 Hz, 3H), 1.95 (d, *J* = 1.98 Hz, 3H).

^{13}C NMR (101 MHz, CDCl$_3$) δ 161.6 (d, *J* = 246.3 Hz), 142.5 (d, *J* = 3.4 Hz), 139.0, 134.0, 130.2, 129.8 (d, *J* = 7.8 Hz), 121.7, 119.4, 118.3, 115.2 (d, *J* = 21.1 Hz), 110.6, 108.2, 48.6, 29.5, 10.3.

^{19}F NMR (377 MHz, CDCl$_3$) δ -116.03 (d, *J* = 4.13 Hz).

HRMS (ESI, m/z): Mass calcd. for $C_{23}H_{19}F_2N$ [M-H], 346.1413; found 346.1423.

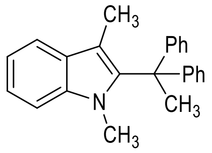

5

2-(1,1-diphenylethyl)-1,3-dimethyl-1*H*-indole (5): White solid, 95% yield, 30.9 mg. m.p.103-105 °C.

^{1}H NMR (400 MHz, CDCl$_3$) δ 7.53 (dt, *J* = 7.86, 1.06 Hz, 1H), 7.30 – 7.26 (m, 4H), 7.24 – 7.19 (m, 8H), 7.12 – 7.08 (m, 1H), 3.18 (s, 3H), 2.33 (s, 3H), 1.74 (s, 3H).

^{13}C NMR (101 MHz, CDCl$_3$) δ 147.5, 140.4, 137.5, 129.3, 128.4, 128.3, 126.4, 121.6, 118.8, 118.4, 109.6, 108.6, 50.3, 33.3, 31.8, 10.5.

HRMS (ESI, m/z): Mass calcd. for $C_{24}H_{23}N$ [M-H], 326.1903; found 326.1889.

VI. ^{1}H NMR, ^{13}C NMR and ^{19}F NMR spectra

2-(1,1-diphenylethyl)-3-methyl-1*H*-indole (3a):

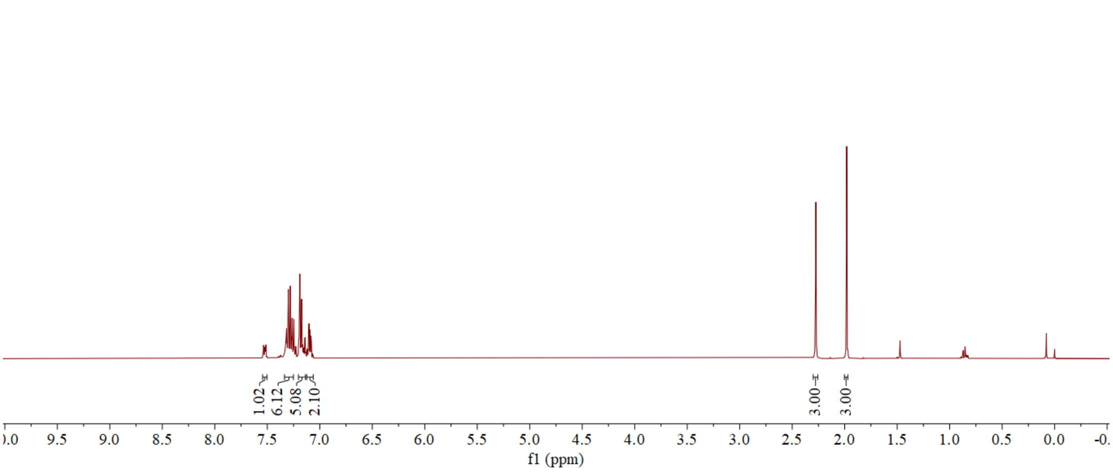

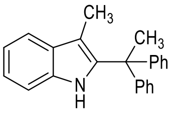

3a

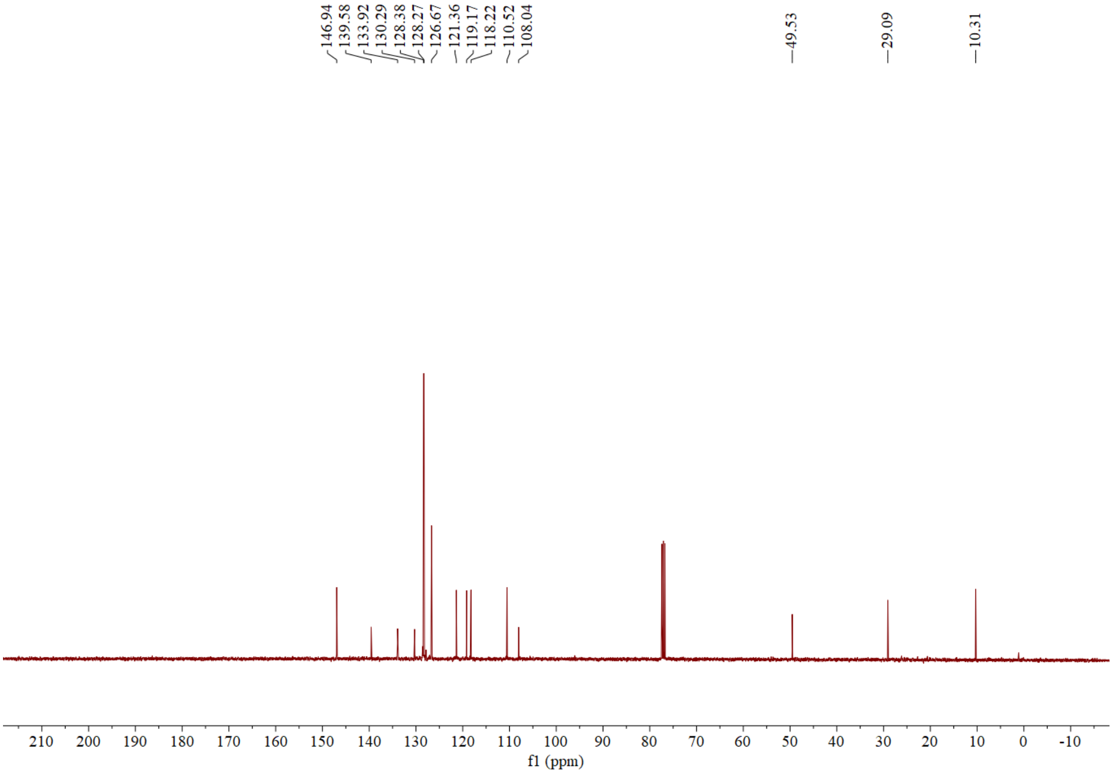

2-(1,1-diphenylethyl)-3,5-dimethyl-1*H*-indole (3b):

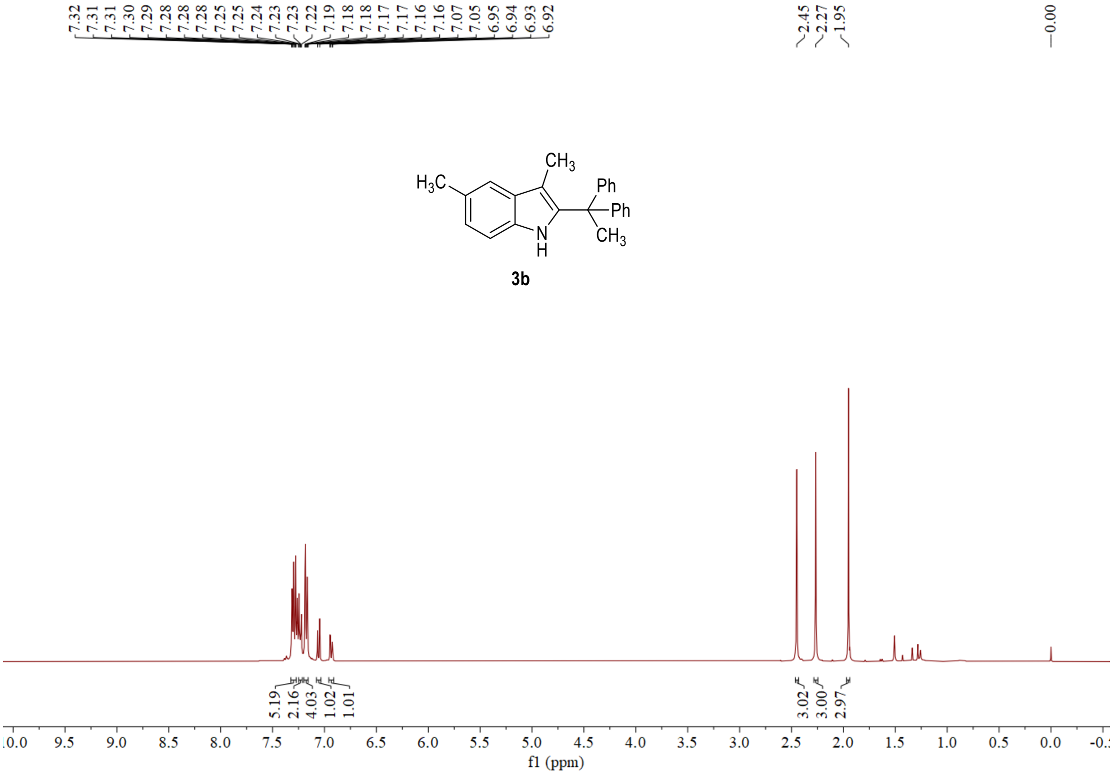

3b

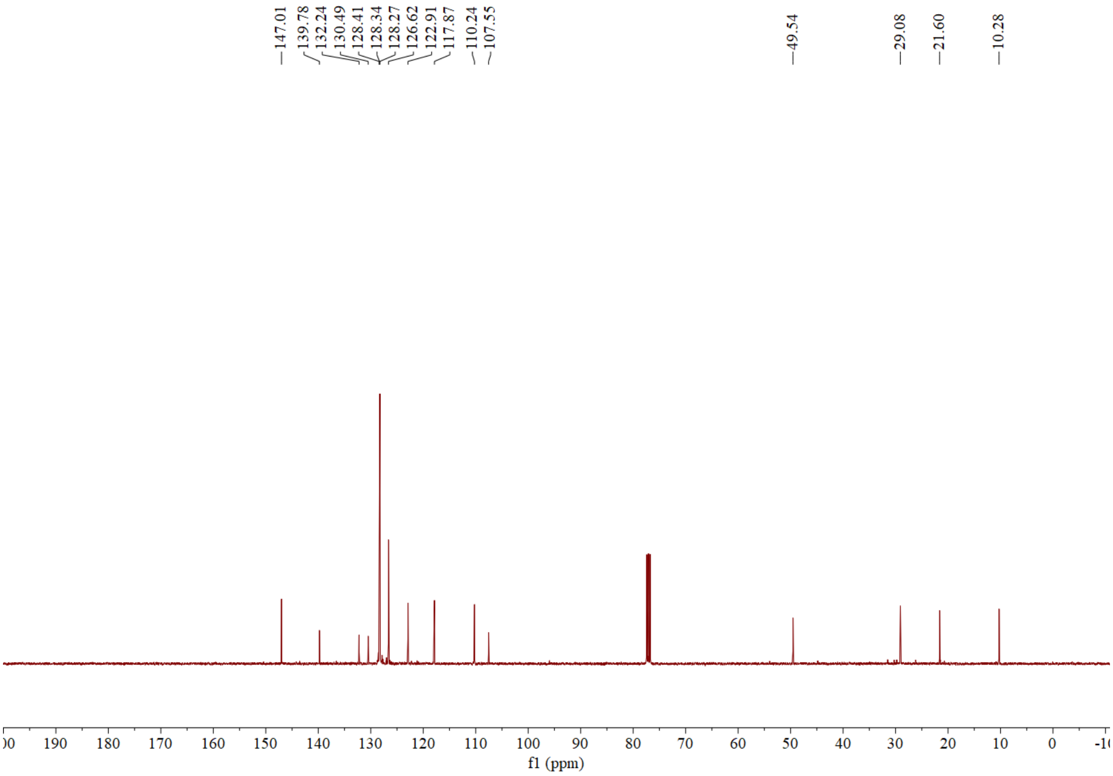

2-(1,1-diphenylethyl)-5-fluoro-3-methyl-1*H*-indole (3c):

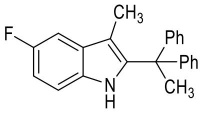

3c

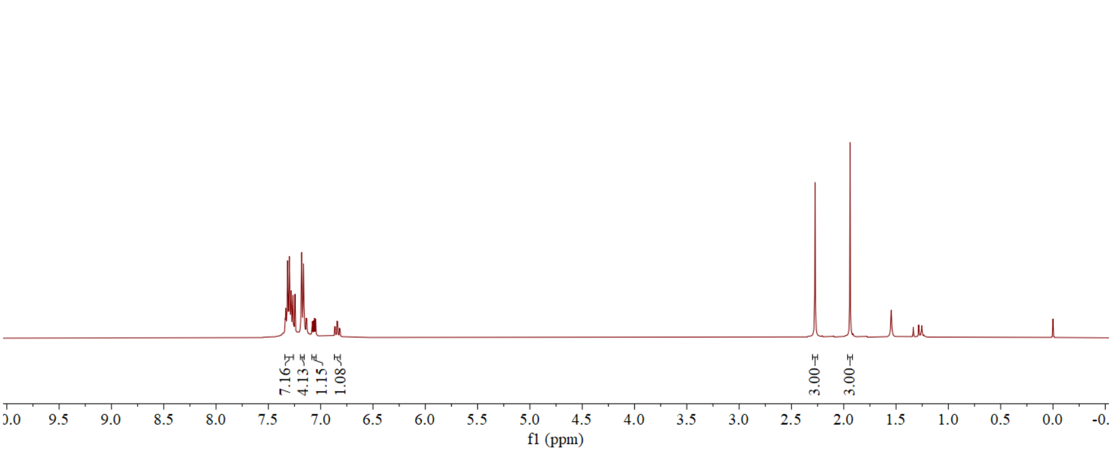

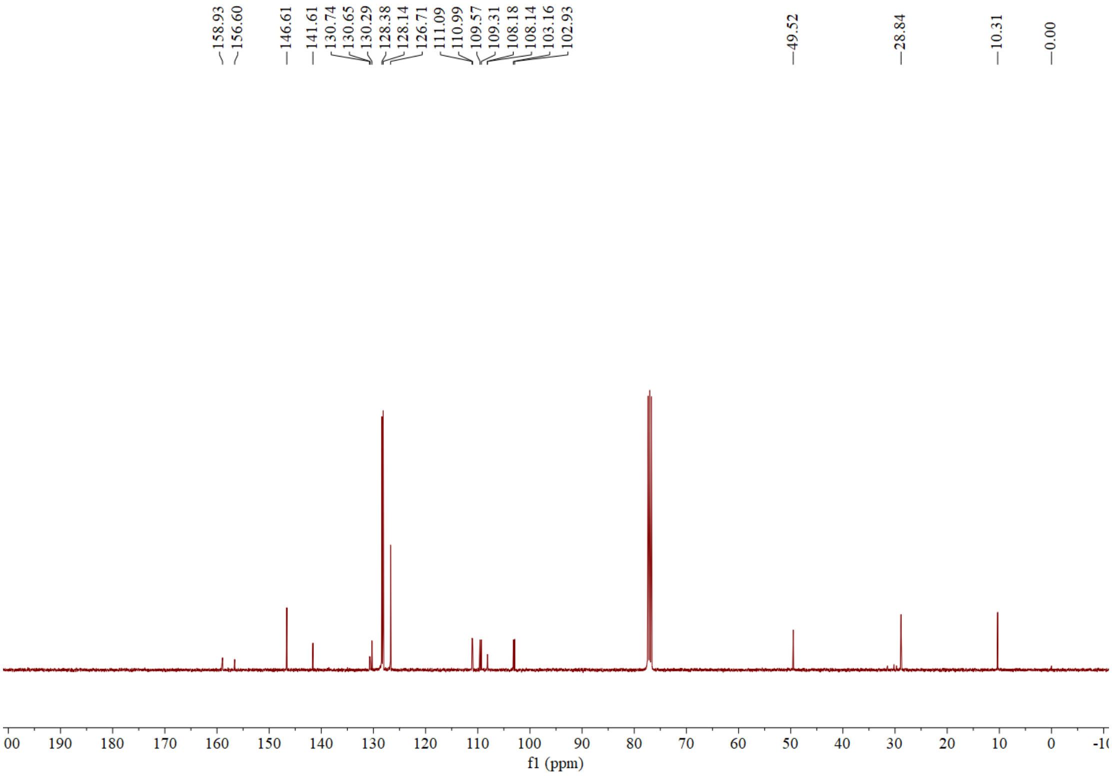

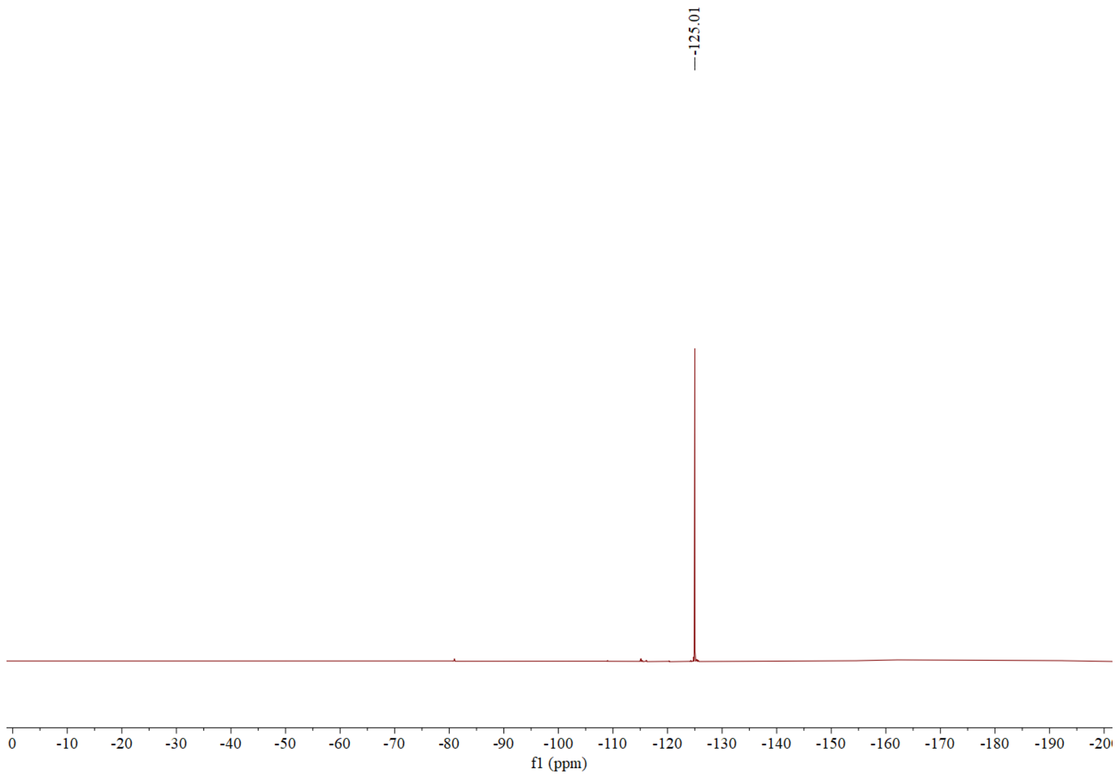

-125.01
0
-10
-20
-30
-40
-50
-60
-70
-80
-90
-100
-110
-120
-130
-140
-150
-160
-170
-180
-190
f1 (ppm)

5-chloro-2-(1,1-diphenylethyl)-3-methyl-1*H*-indole (3d):

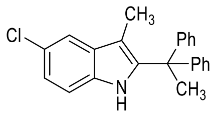

3d

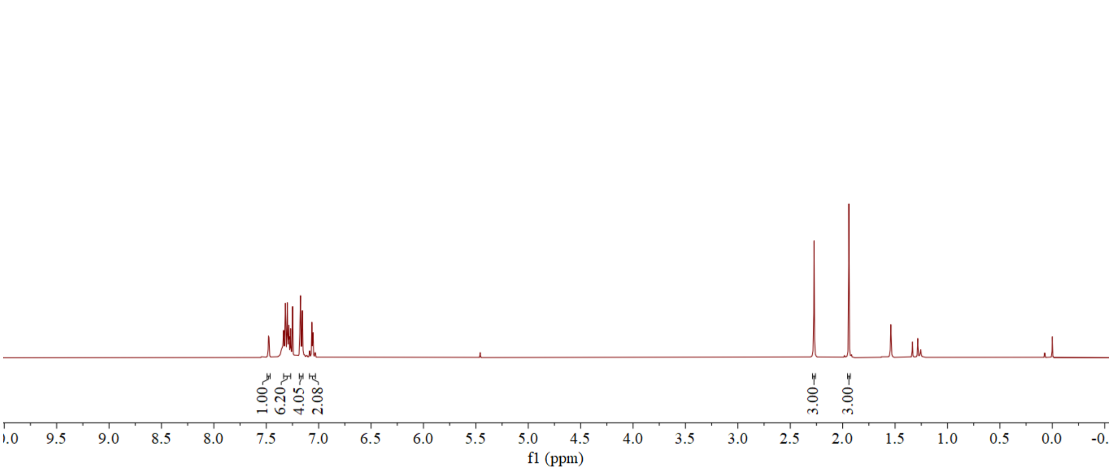

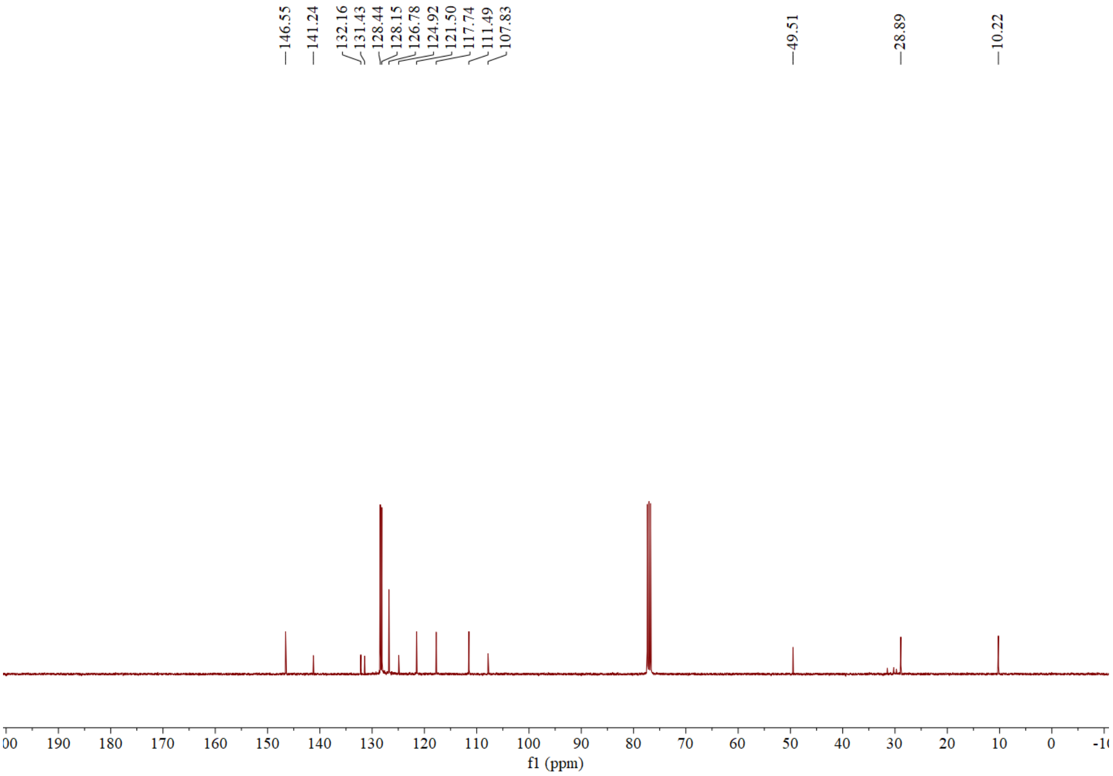

2-(1,1-diphenylethyl)-3,6-dimethyl-1*H*-indole (3e):

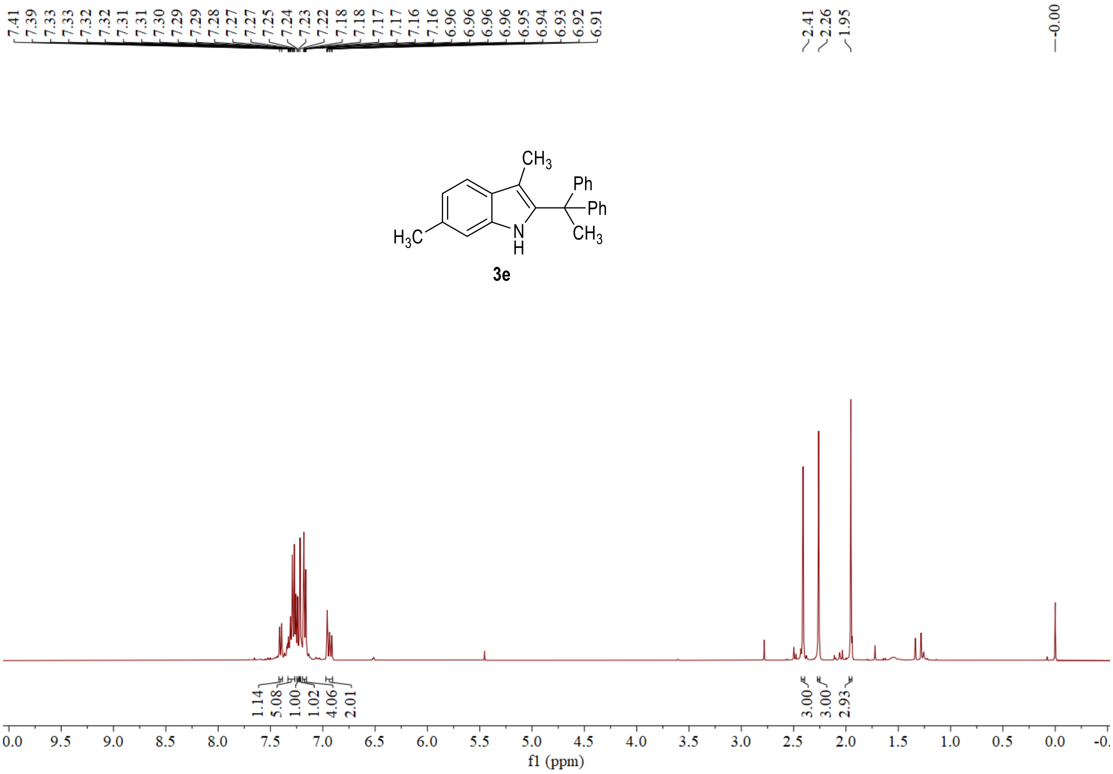

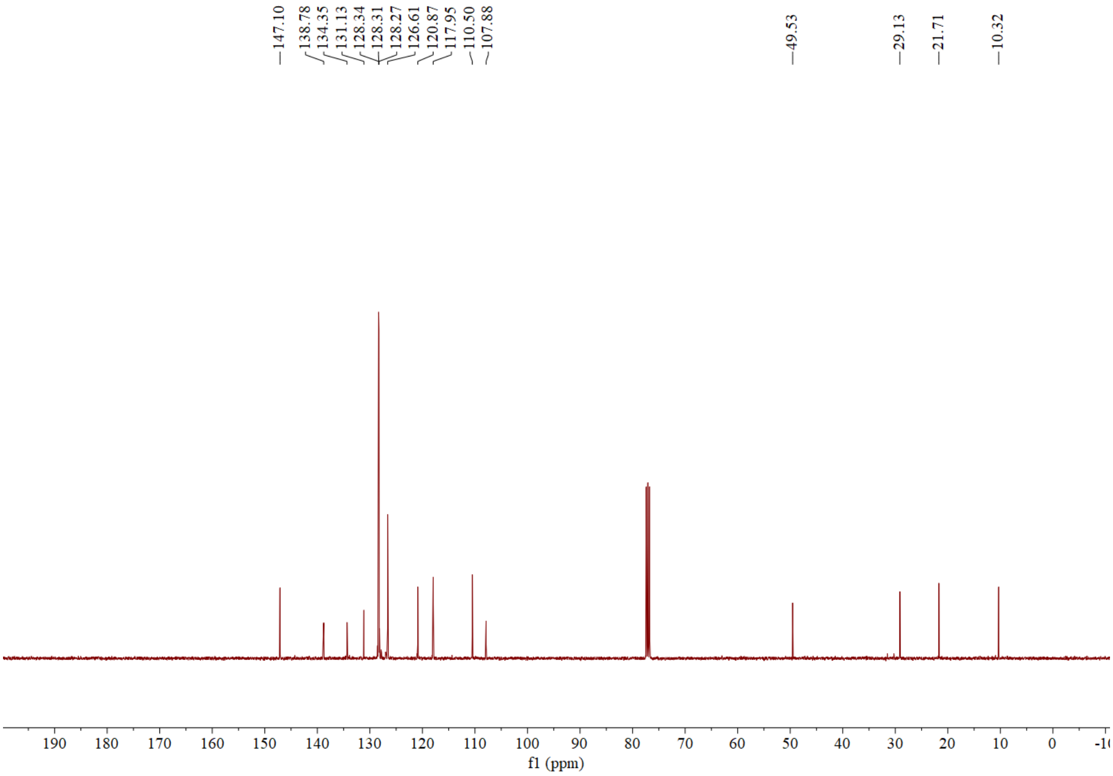

2-(1,1-diphenylethyl)-6-methoxy-3-methyl-1*H*-indole (3f):

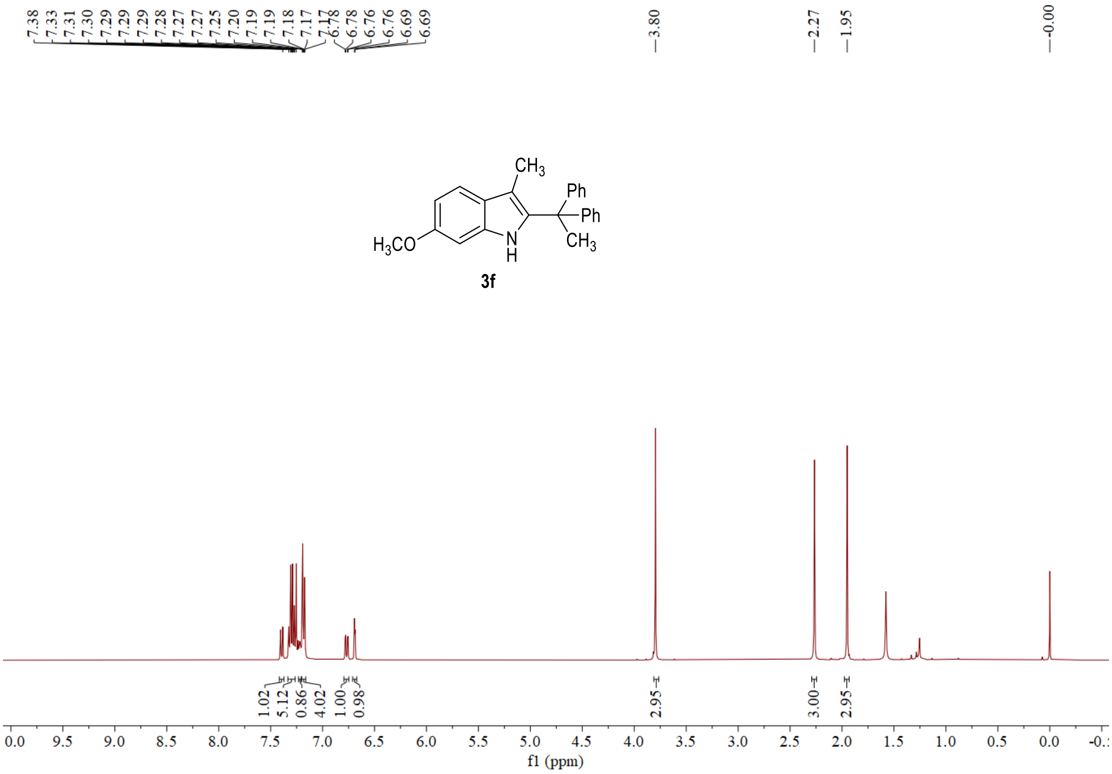

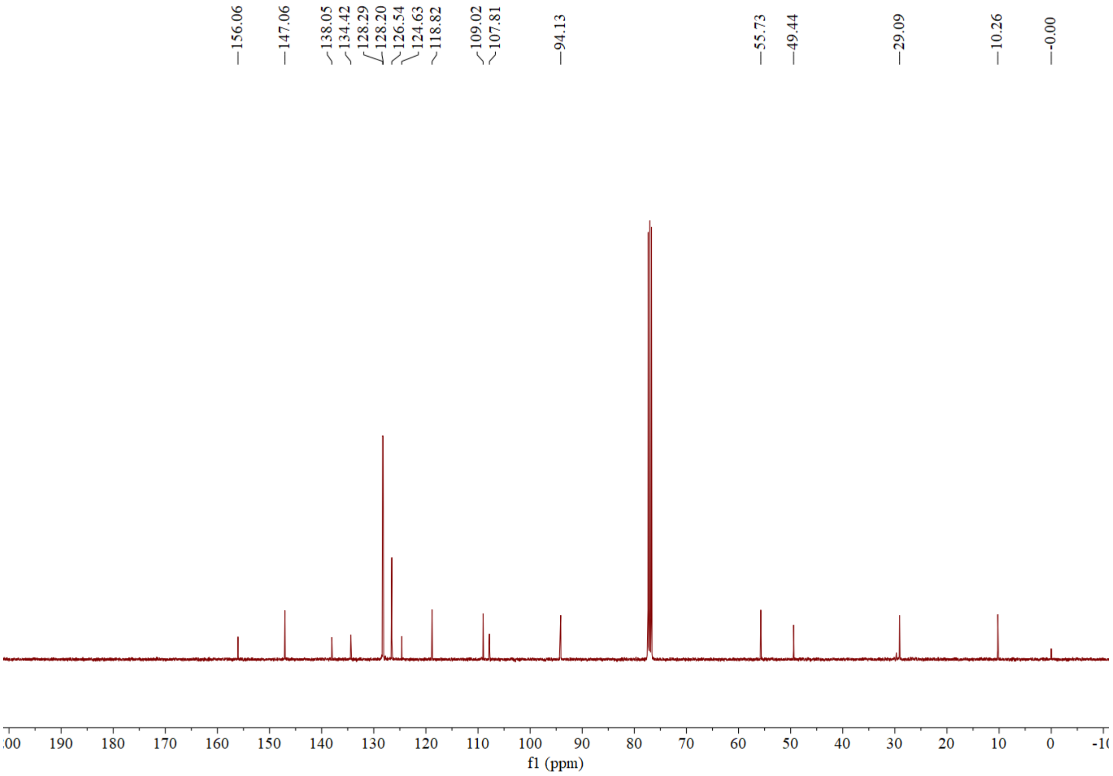

2-(1,1-diphenylethyl)-6-fluoro-3-methyl-1*H*-indole (3g):

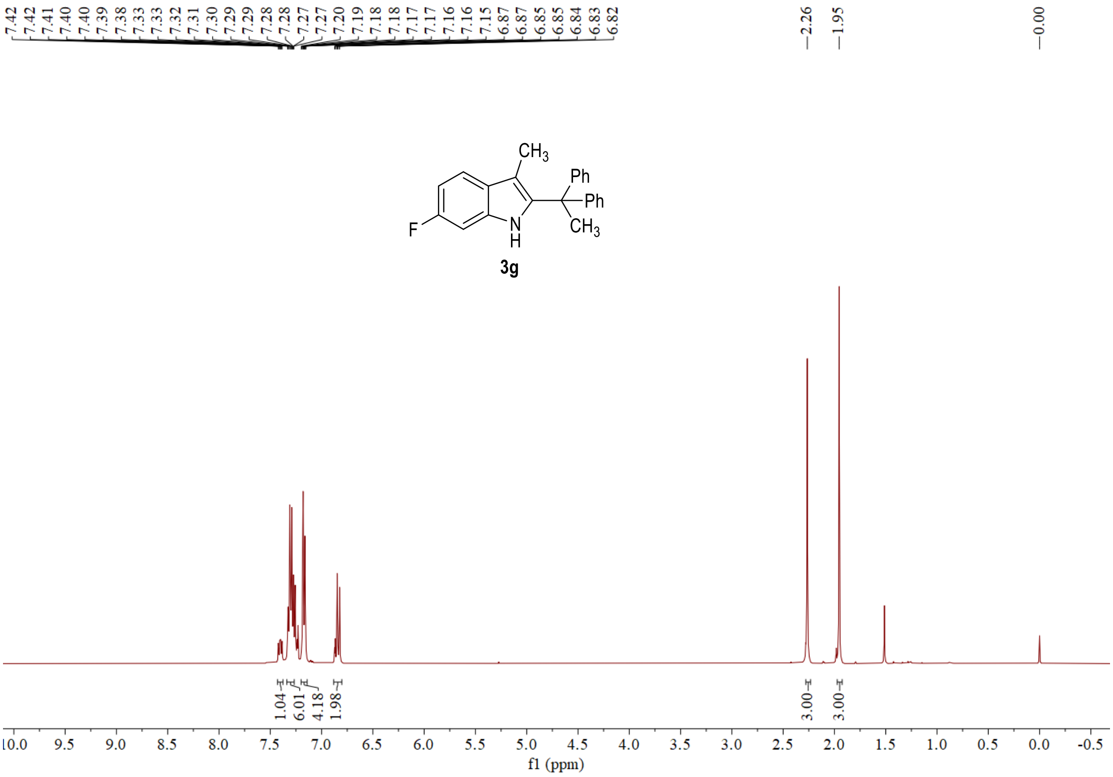

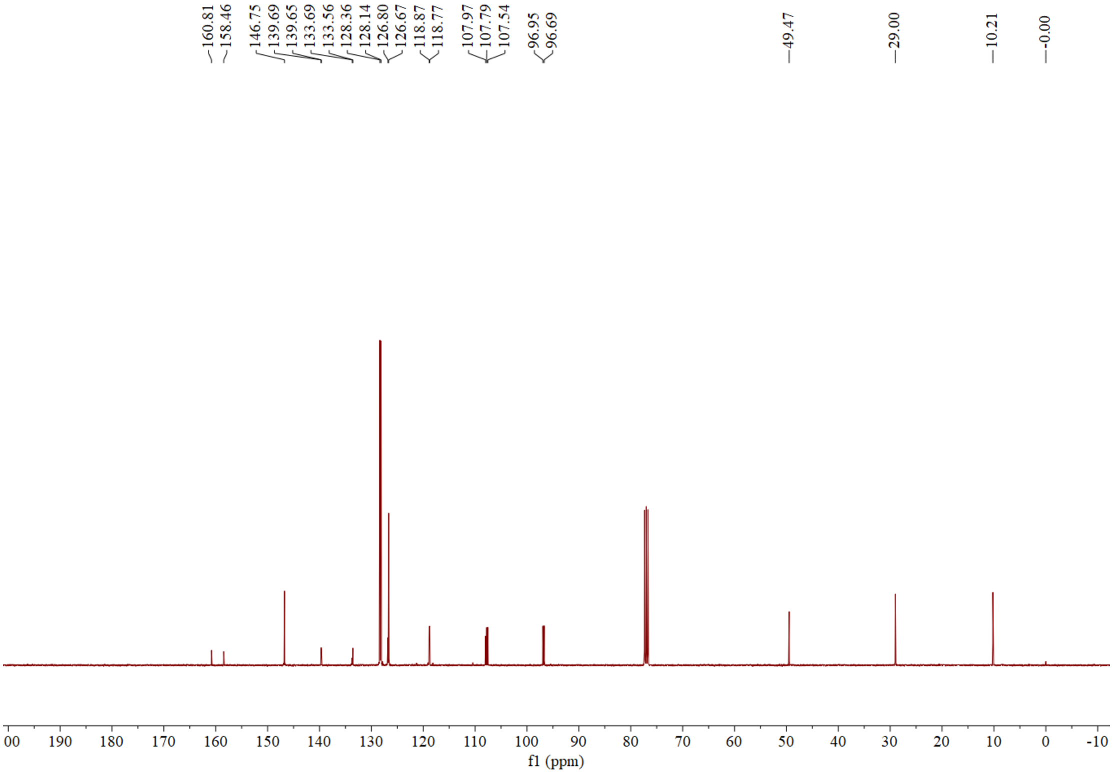

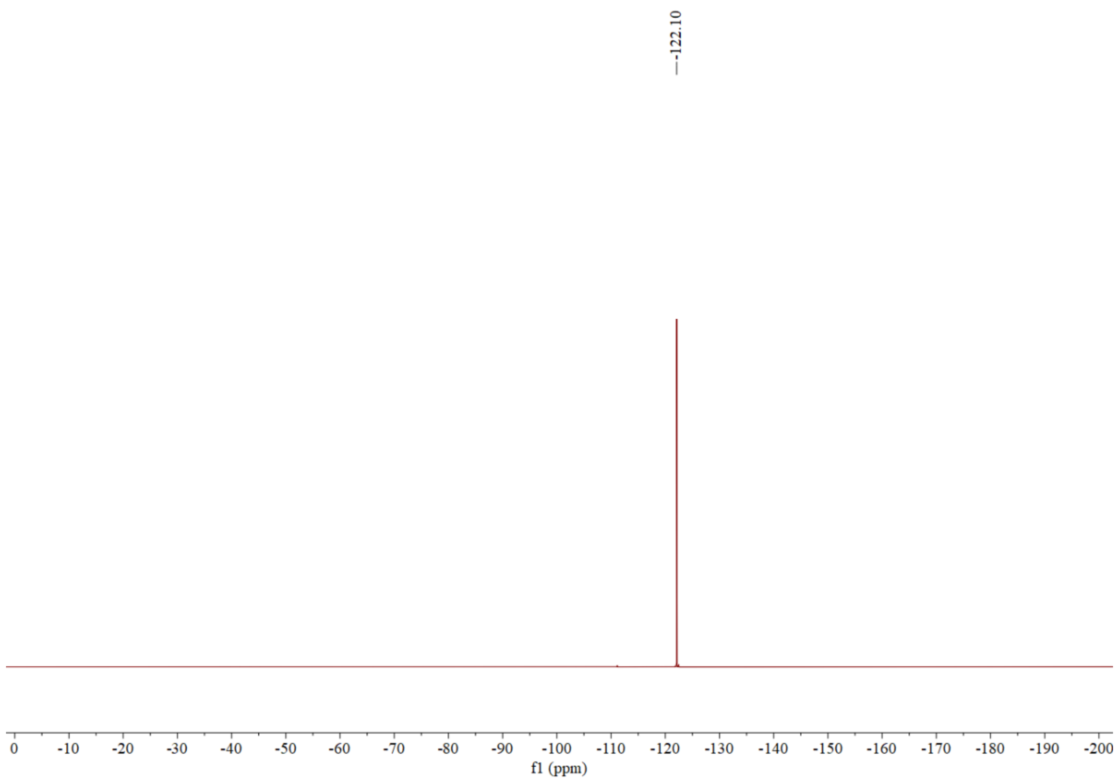

-122.10
0
-10
-20
-30
-40
-50
-60
-70
-80
-90
-100
-110
-120
-130
-140
-150
-160
-170
-180
-190
-200
f1 (ppm)

6-chloro-2-(1,1-diphenylethyl)-3-methyl-1*H*-indole (3h):

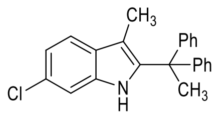

3h

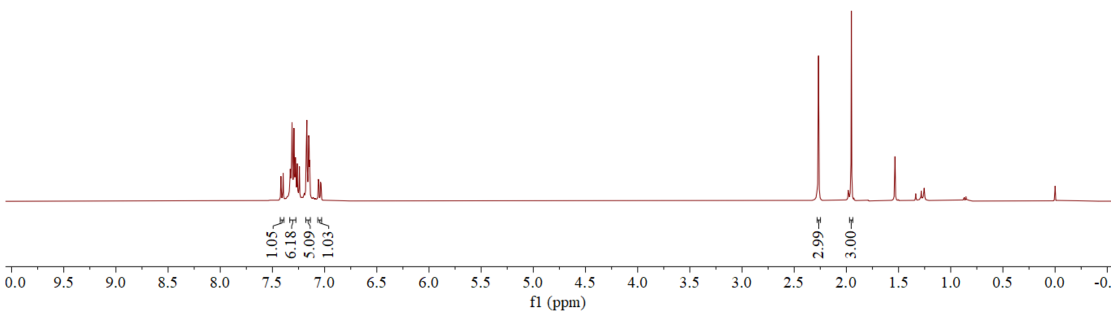

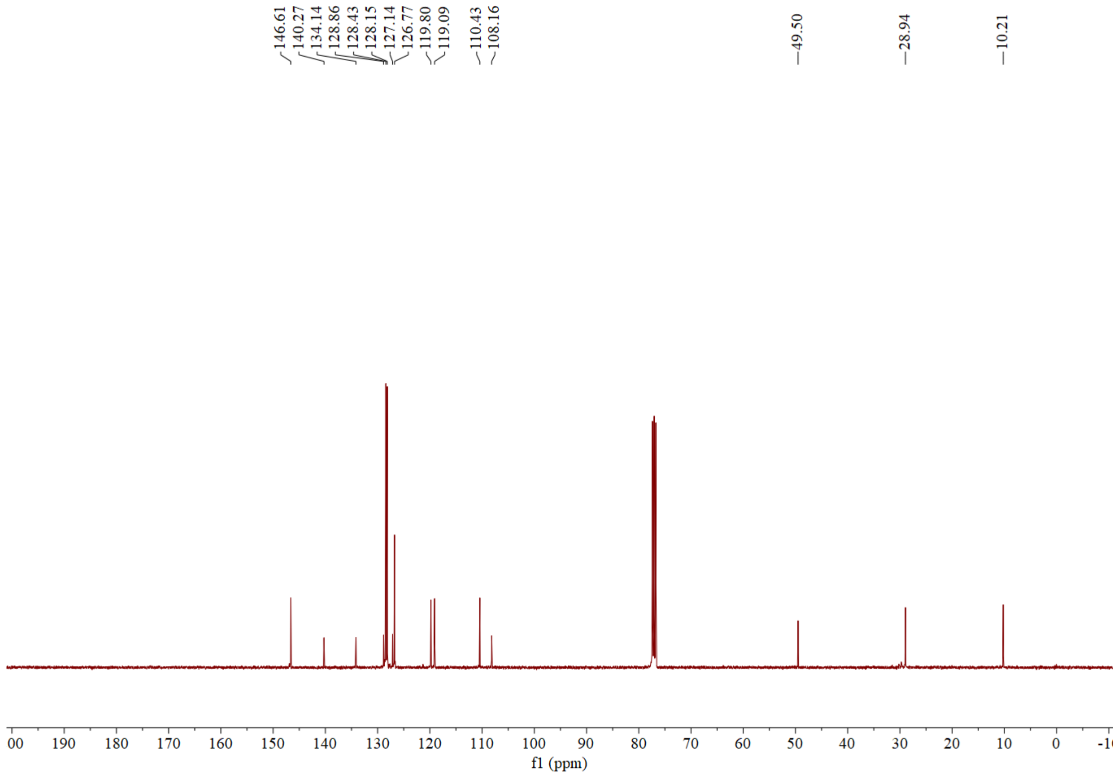

2-(1,1-diphenylethyl)-3,7-dimethyl-1*H*-indole (3i):

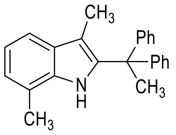

3i

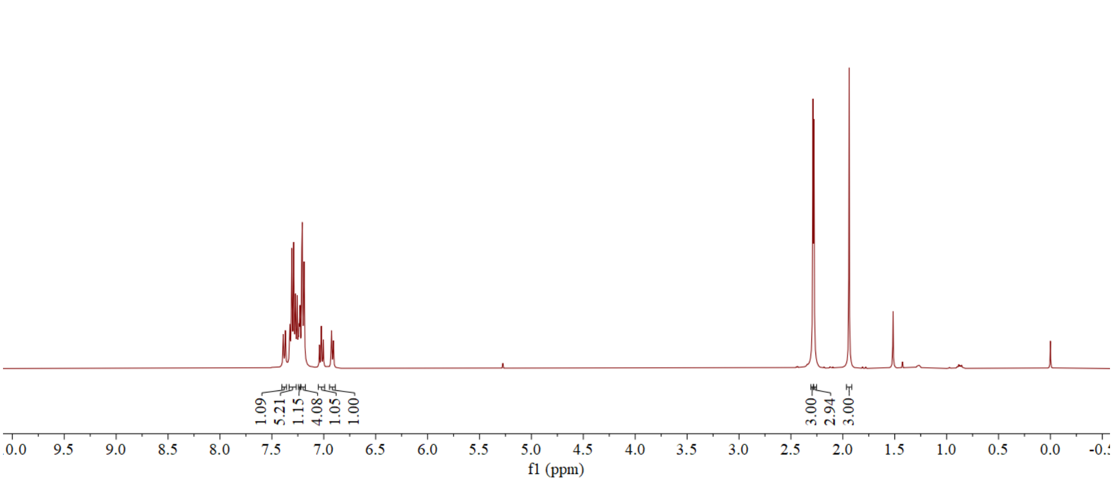

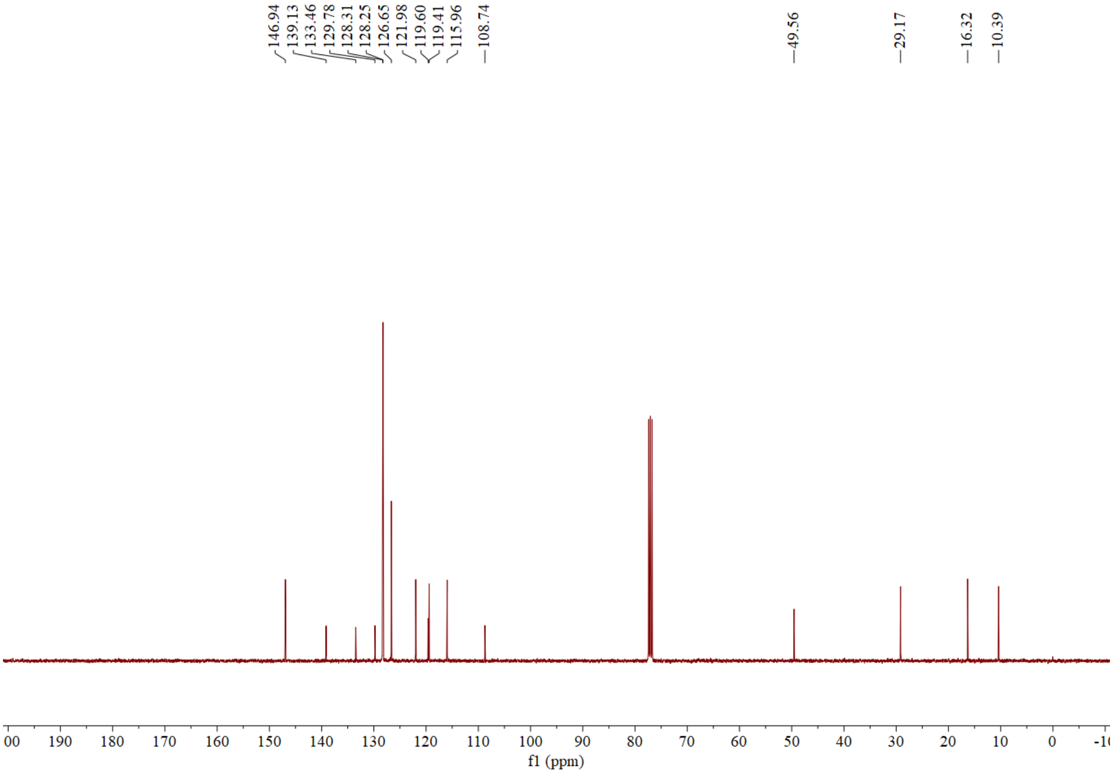

7-chloro-2-(1,1-diphenylethyl)-3-methyl-1*H*-indole (3j):

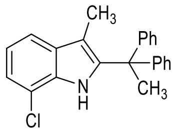

3j

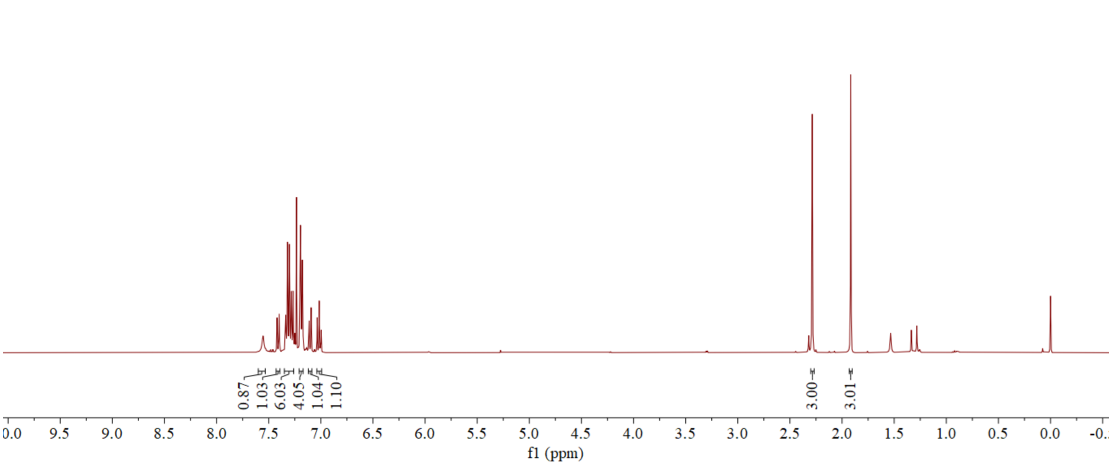

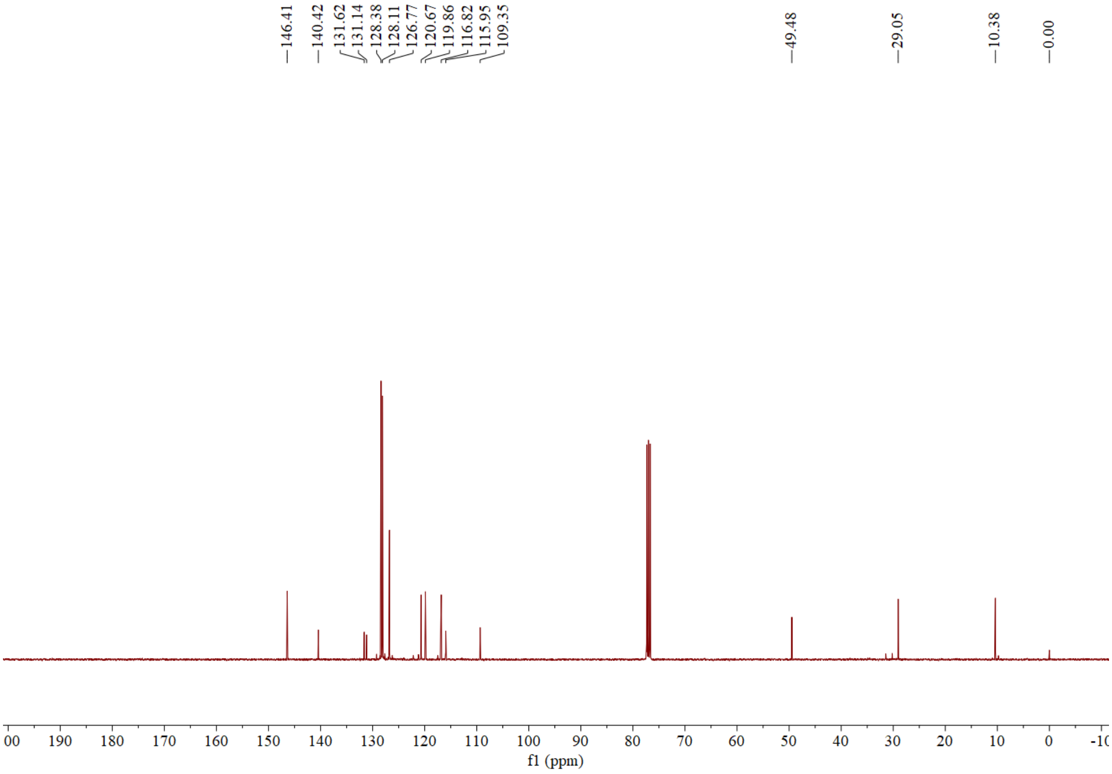

7-bromo-2-(1,1-diphenylethyl)-3-methyl-1*H*-indole (3k):

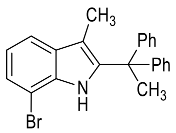

3k

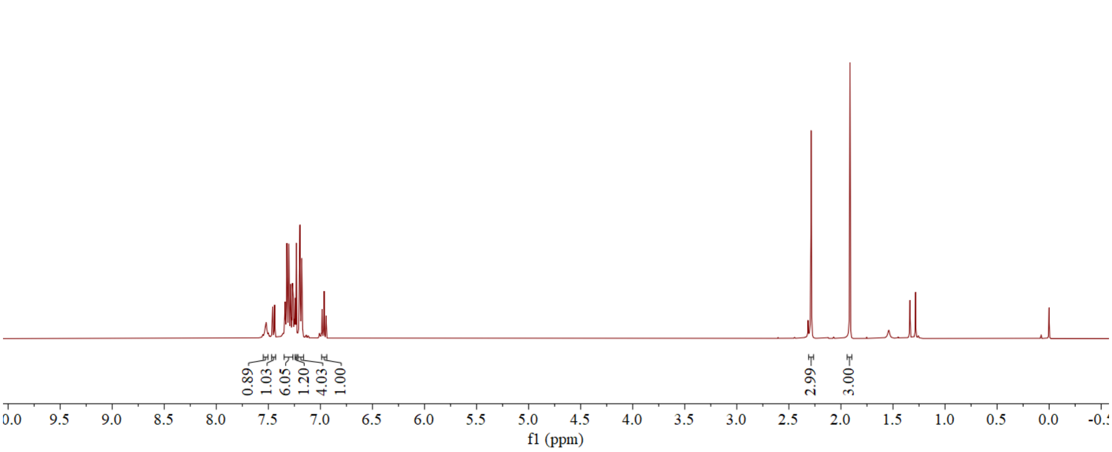

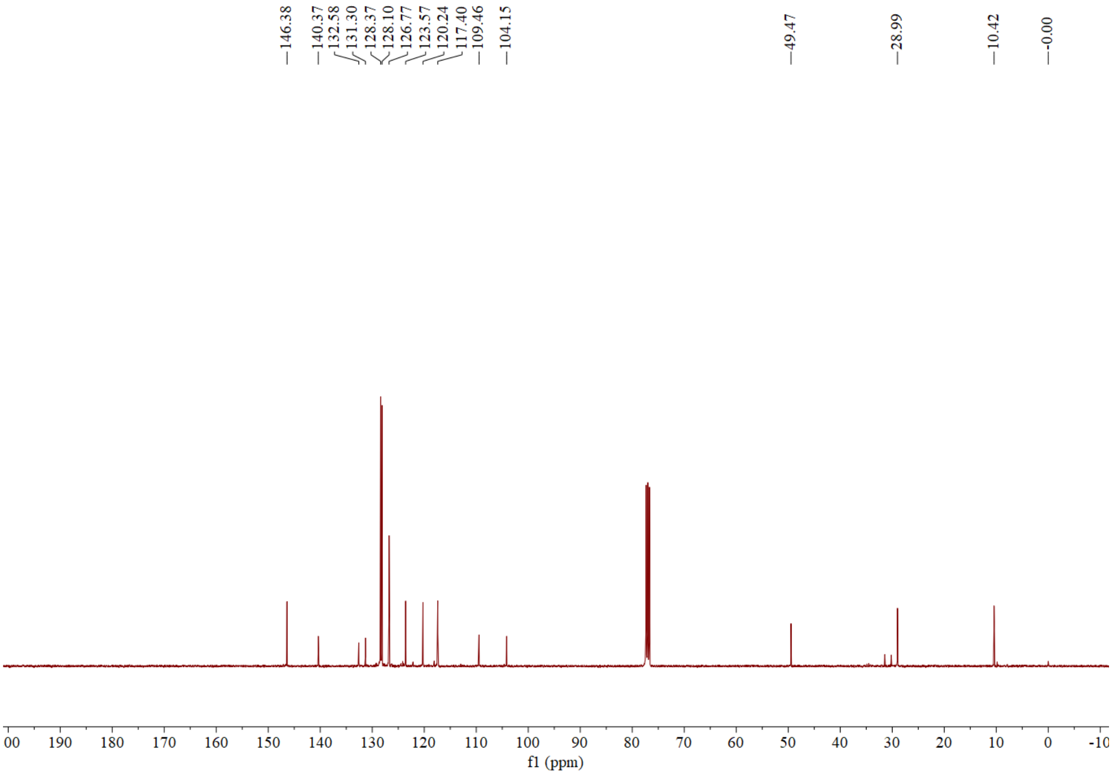

4-chloro-2-(1,1-diphenylethyl)-3-methyl-1*H*-indole (3l):

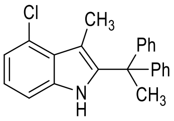

3l

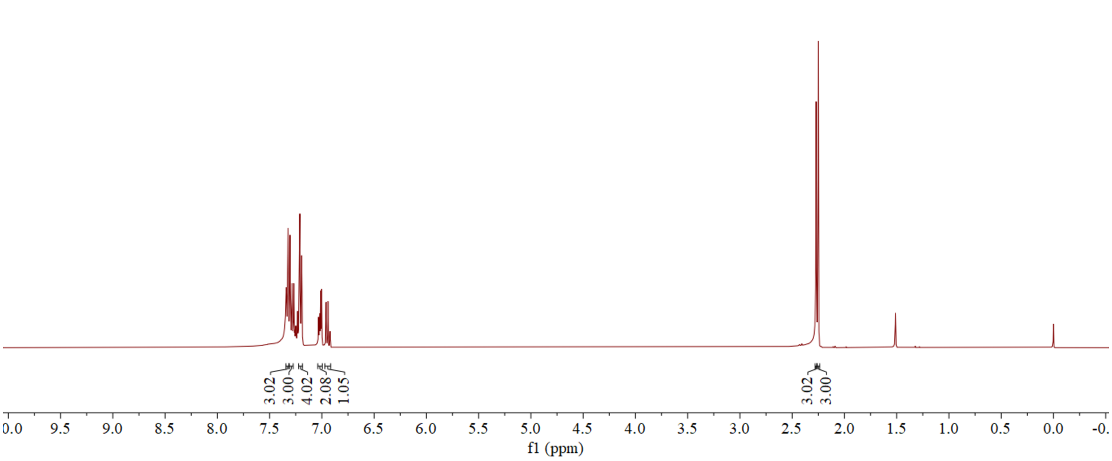

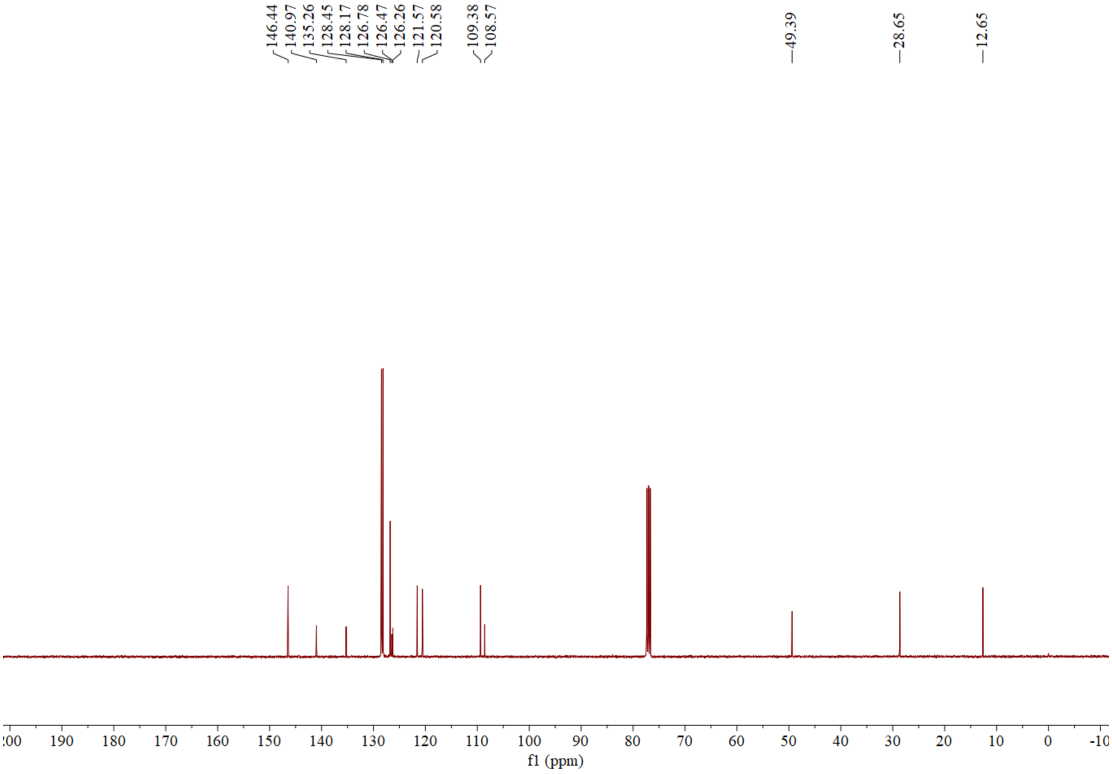

4-bromo-2-(1,1-diphenylethyl)-3-methyl-1*H*-indole (3m):

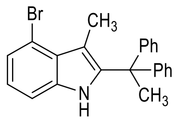

3m

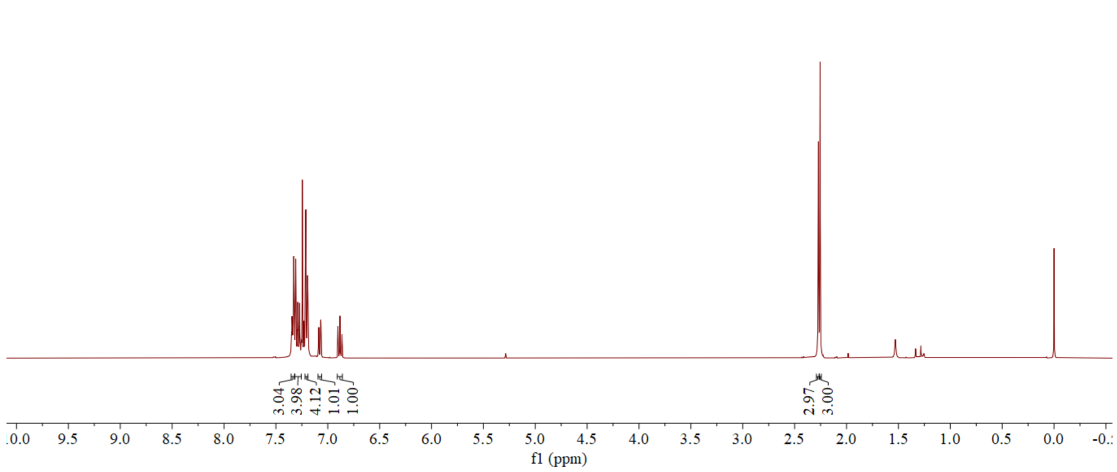

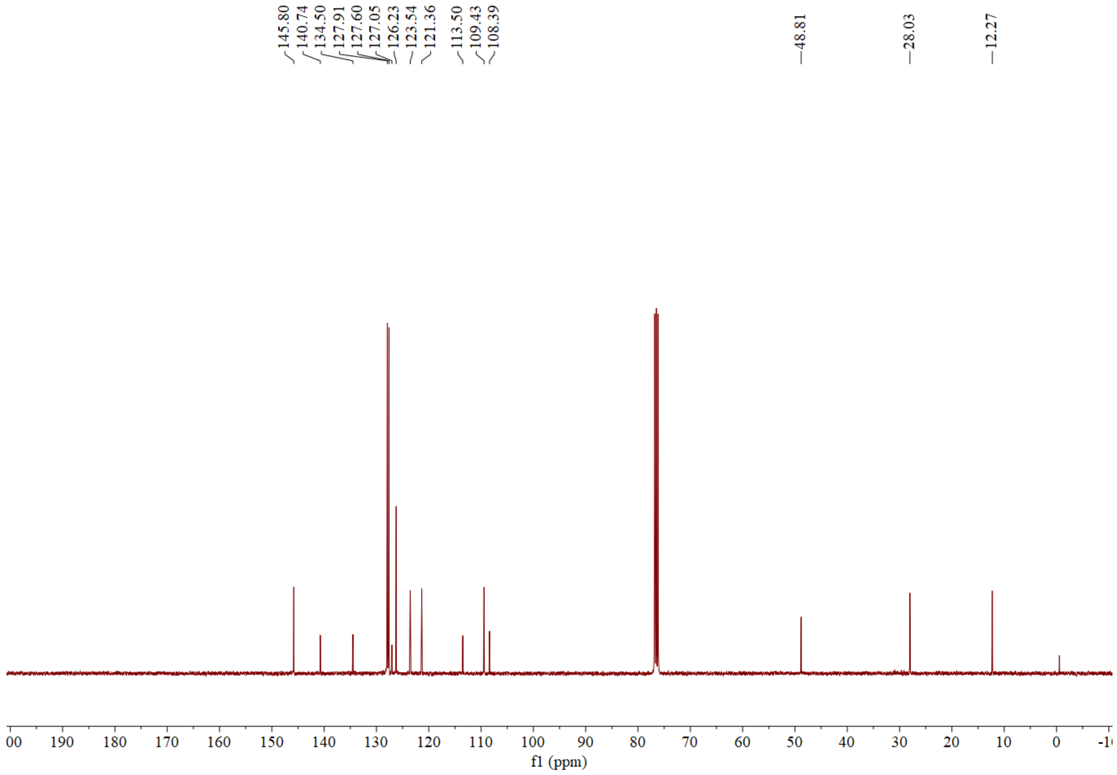

2-(1,1-diphenylethyl)-3-ethyl-1*H*-indole (3n):

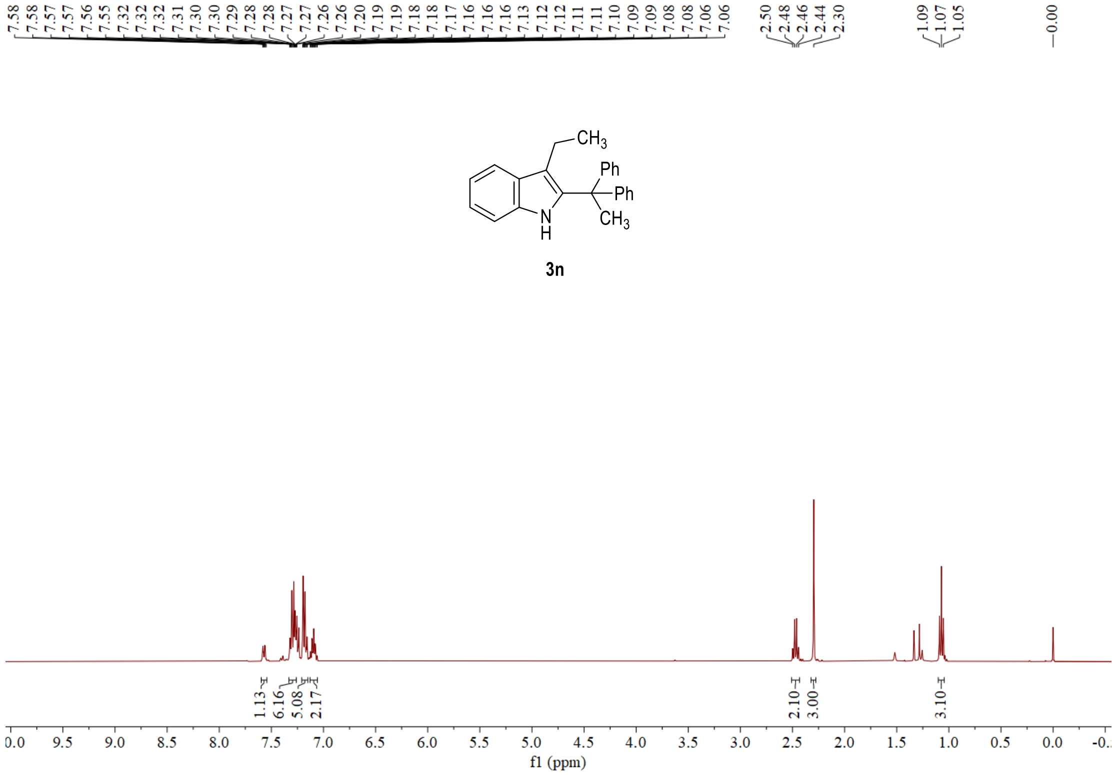

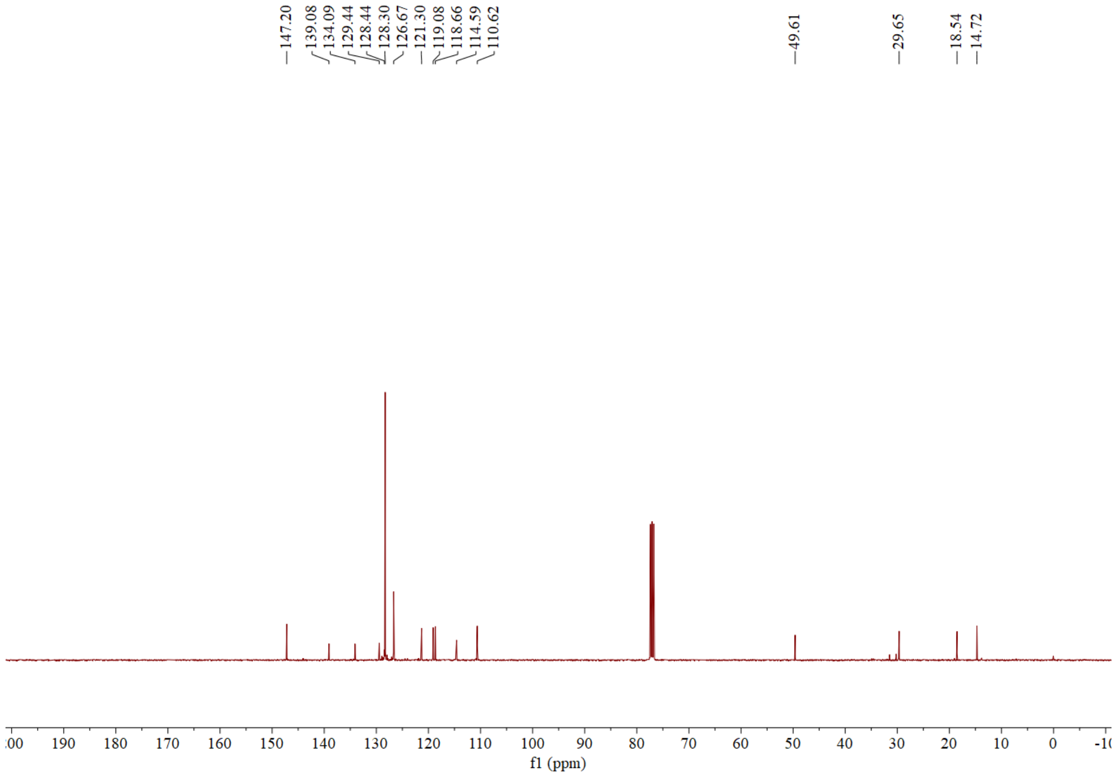

ethyl 2-(2-(1,1-diphenylethyl)-1*H*-indol-3-yl)acetate (3o):

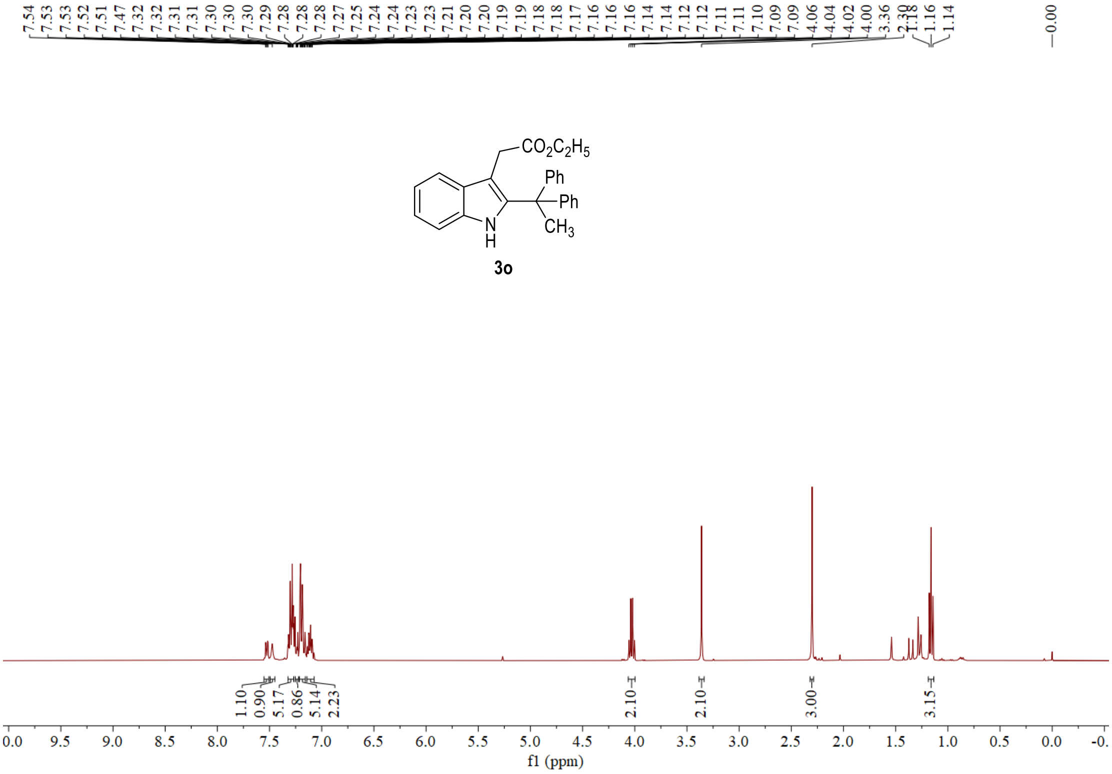

3o

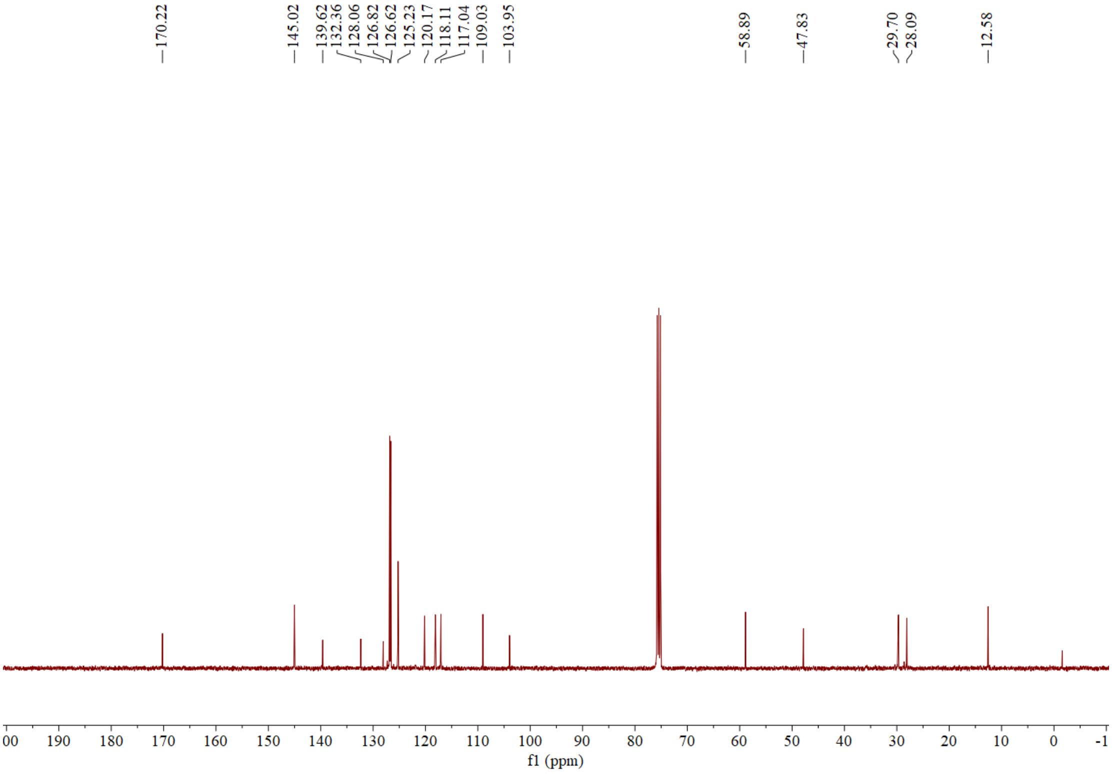

3-methyl-2-(1-phenyl-1-(p-tolyl)ethyl)-1*H*-indole (4a):

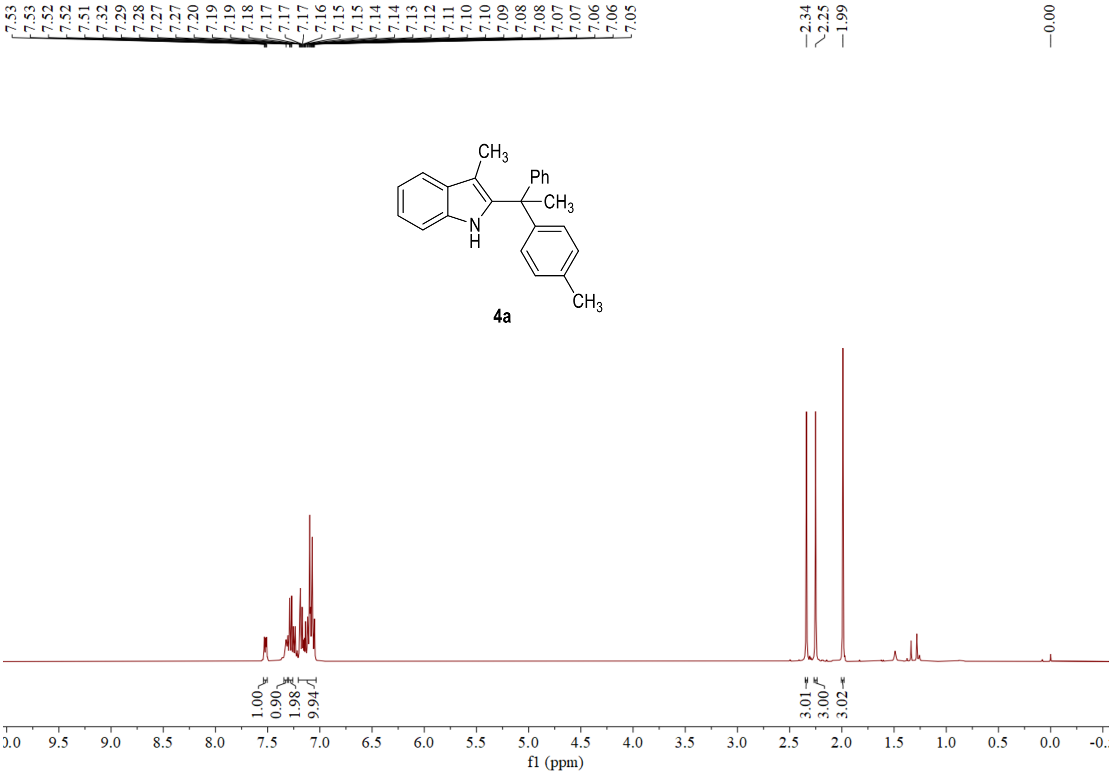

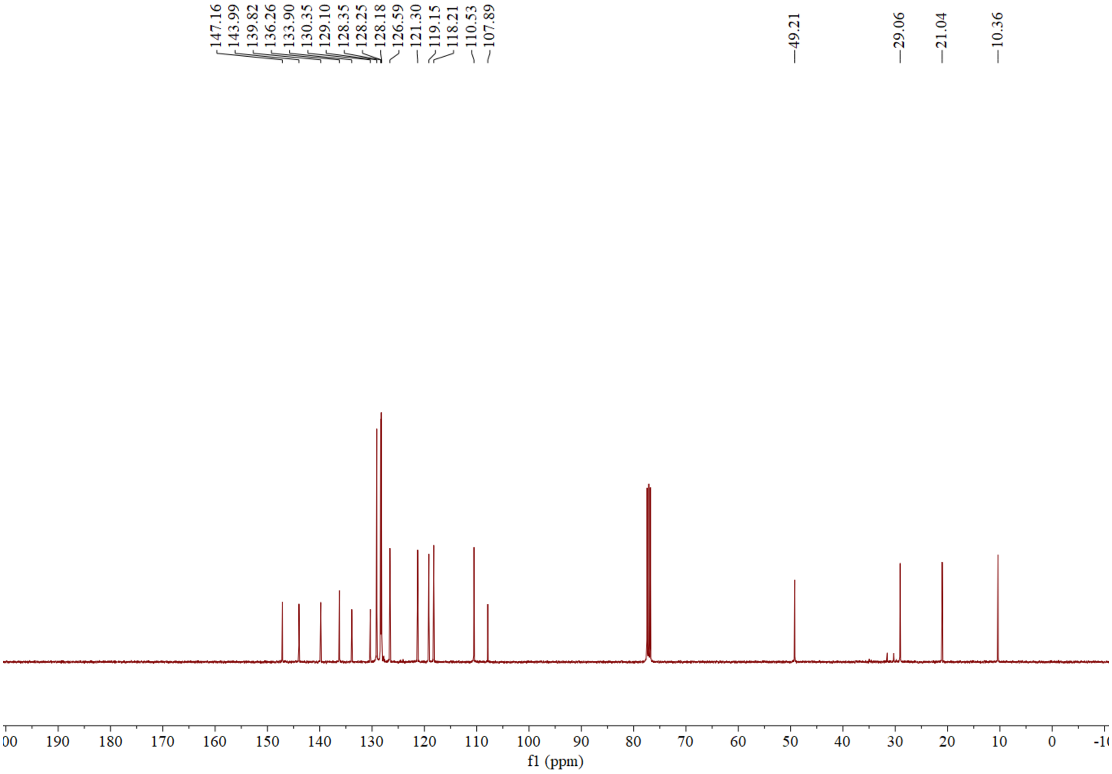

2-(1-([1,1'-biphenyl]-4-yl)-1-phenylethyl)-3-methyl-1*H*-indole (4b):

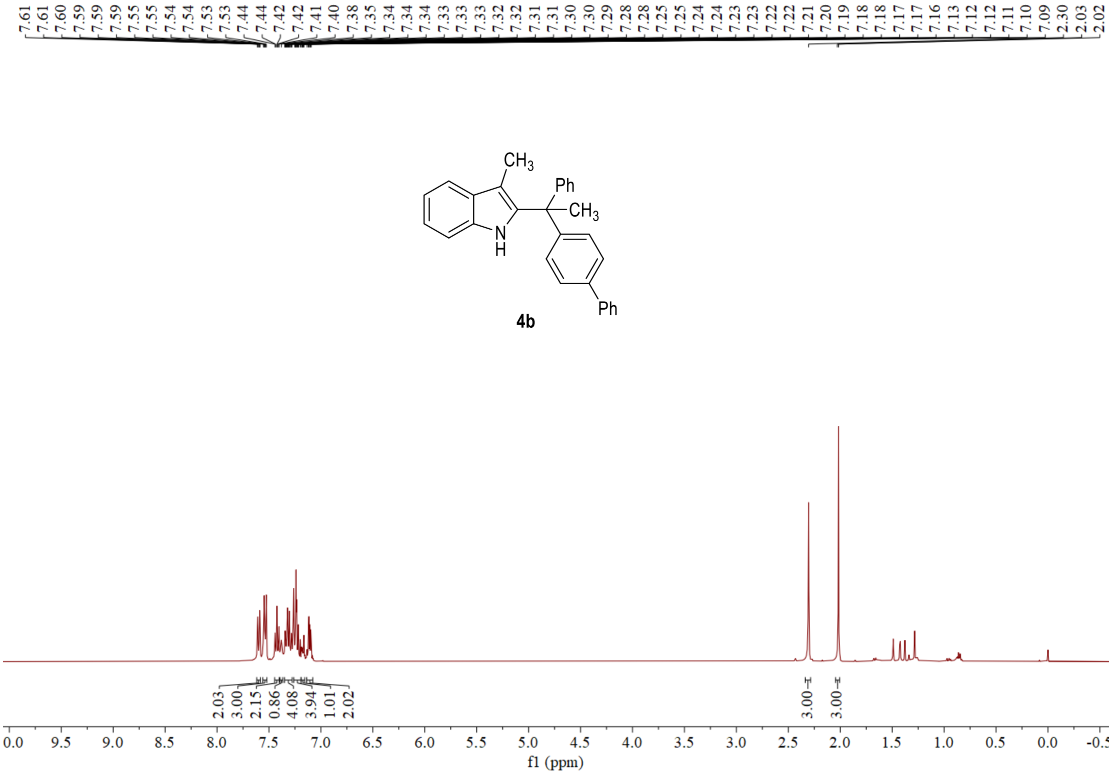

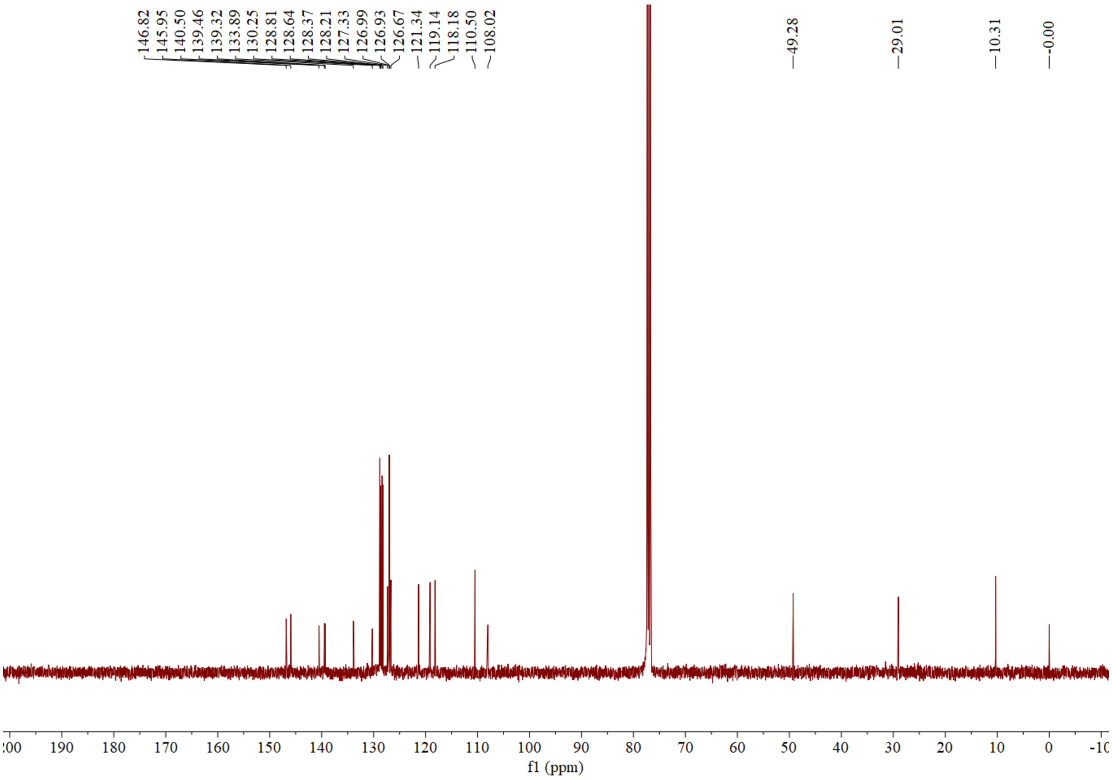

2-(1-(4-methoxyphenyl)-1-phenylethyl)-3-methyl-1*H*-indole (4c):

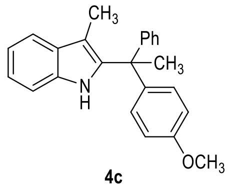

4c

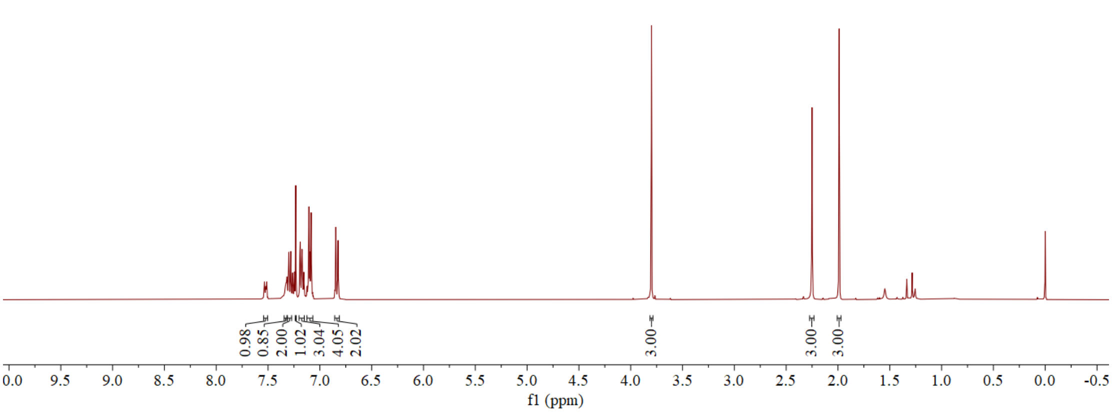

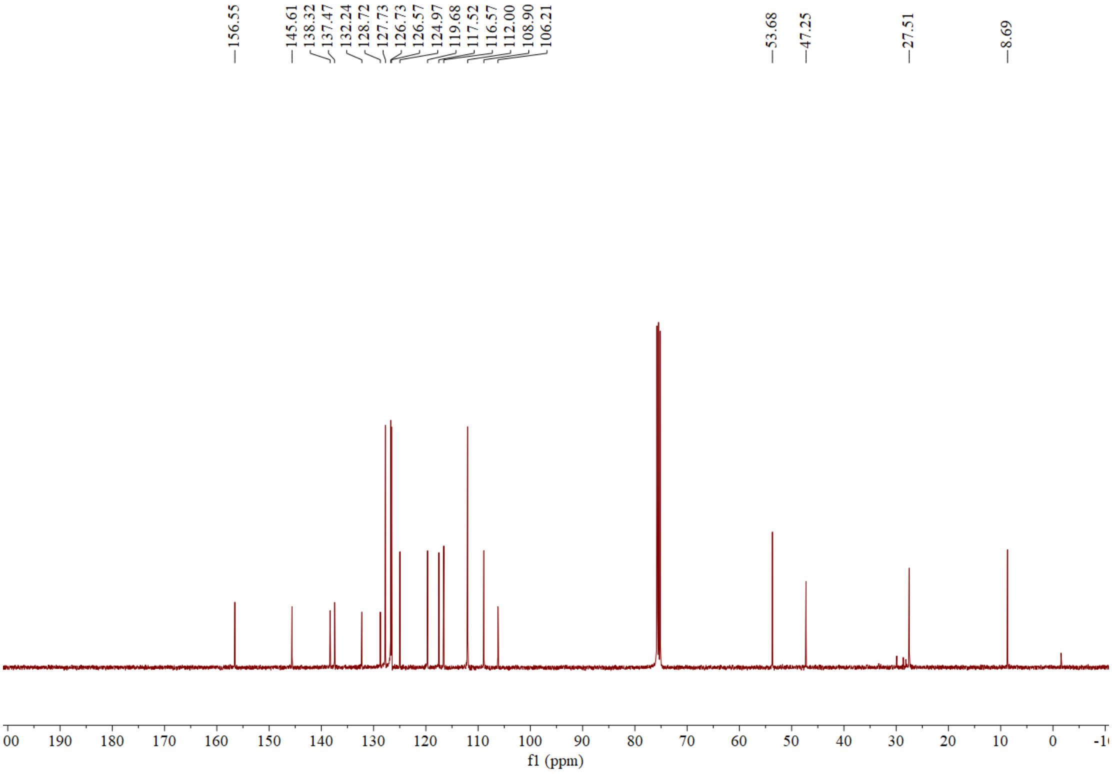

2-(1-(4-fluorophenyl)-1-phenylethyl)-3-methyl-1*H*-indole (4d):

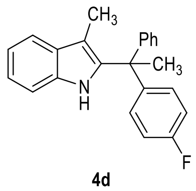

4d

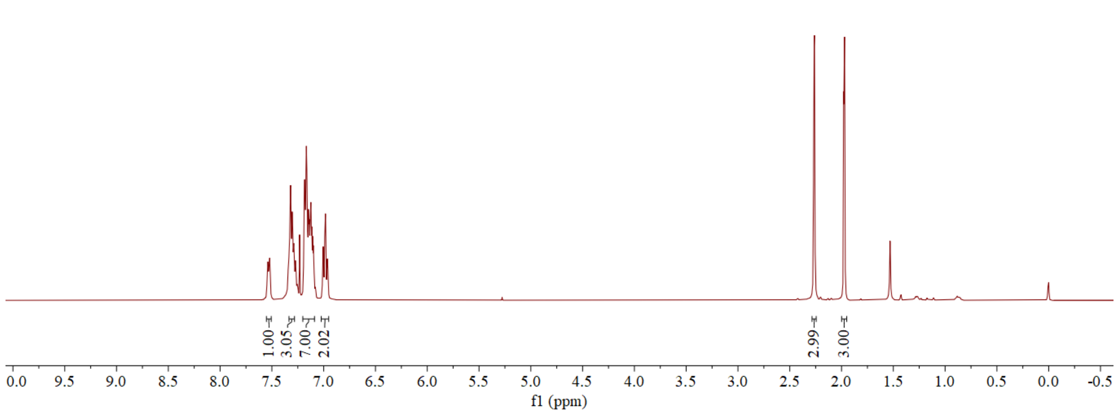

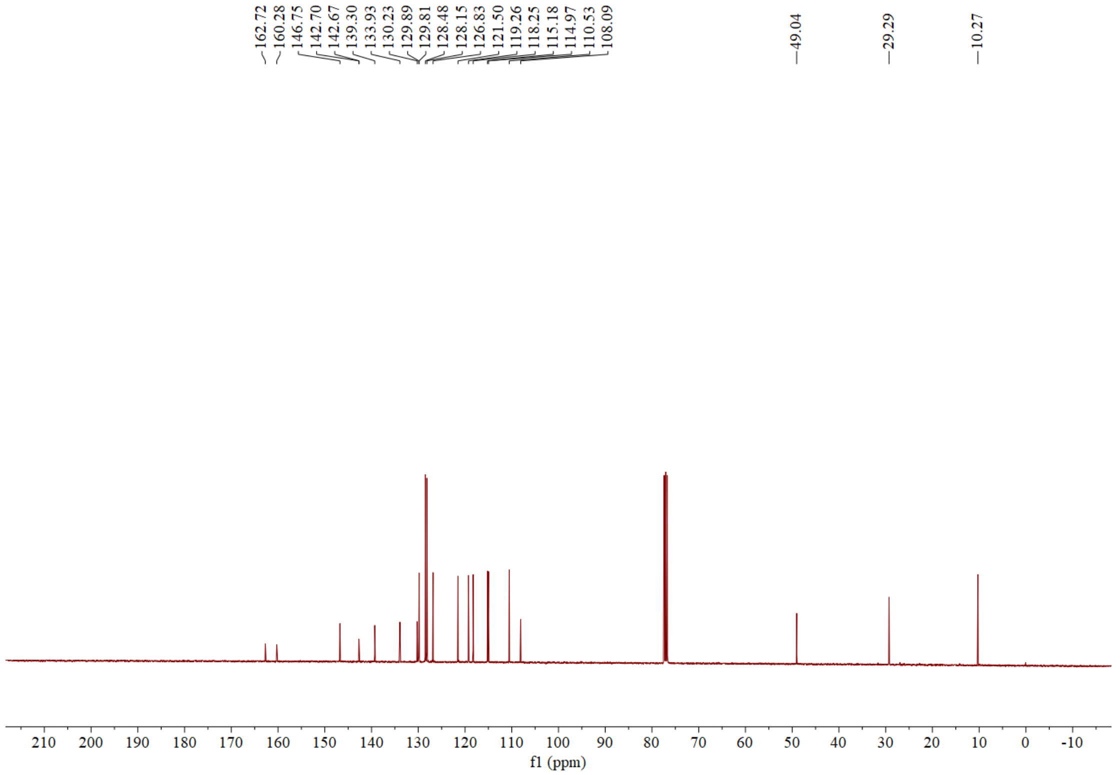

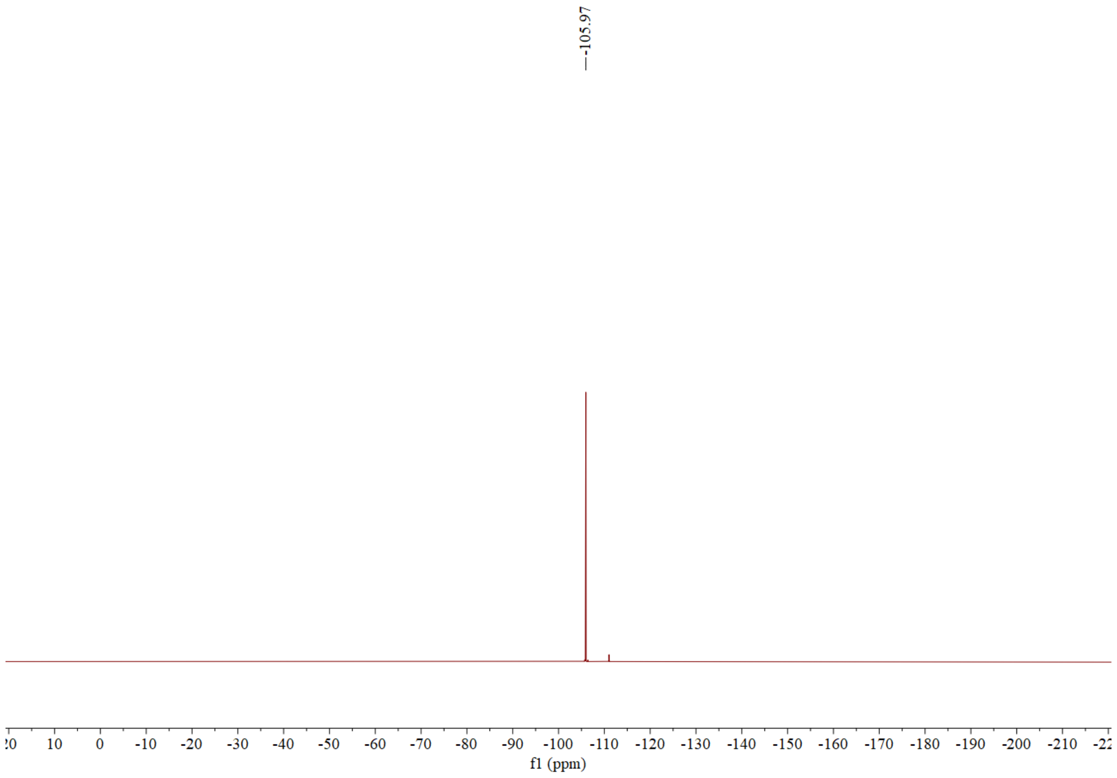
-105.97
f1 (ppm)

3-methyl-2-(1-phenyl-1-(m-tolyl)ethyl)-1*H*-indole (4e):

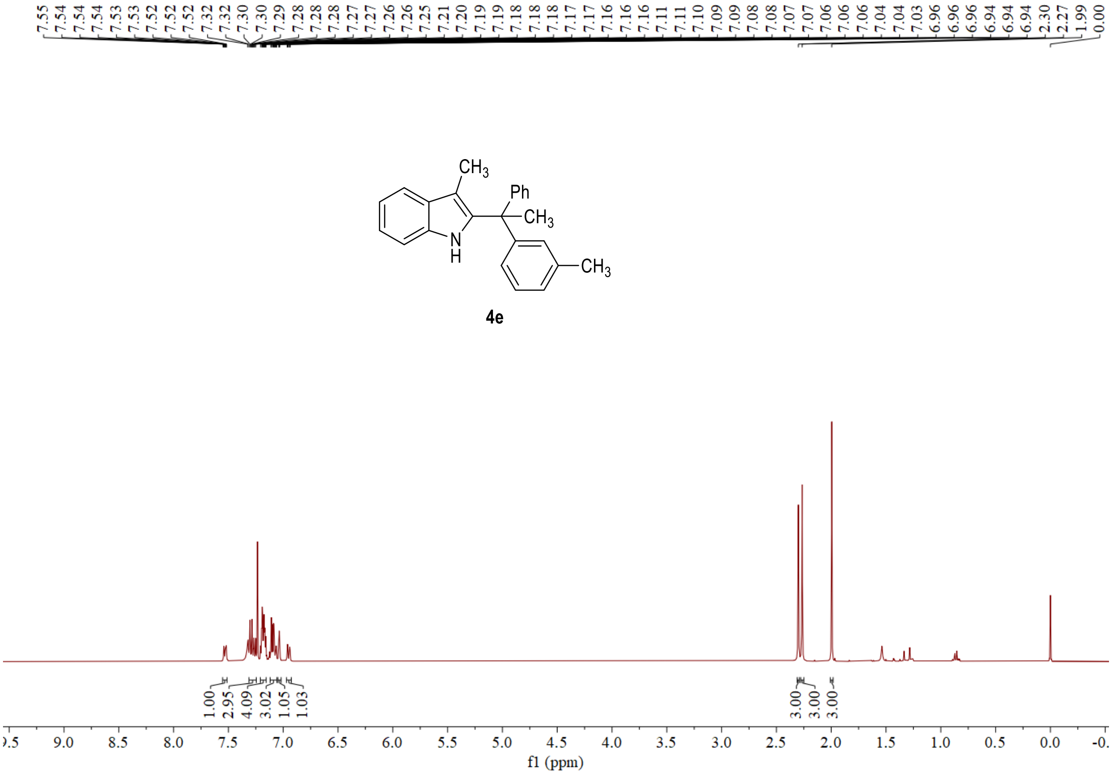

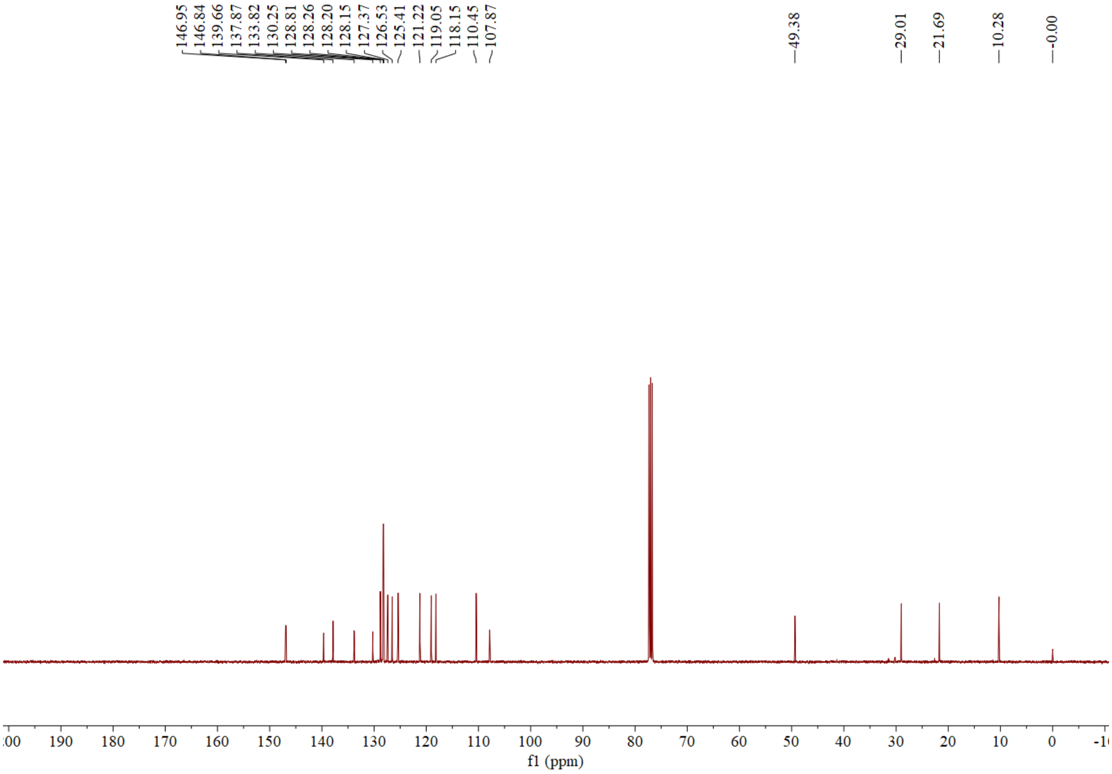

2-(1-(3-chlorophenyl)-1-phenylethyl)-3-methyl-1*H*-indole (4f):

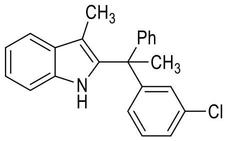

4f

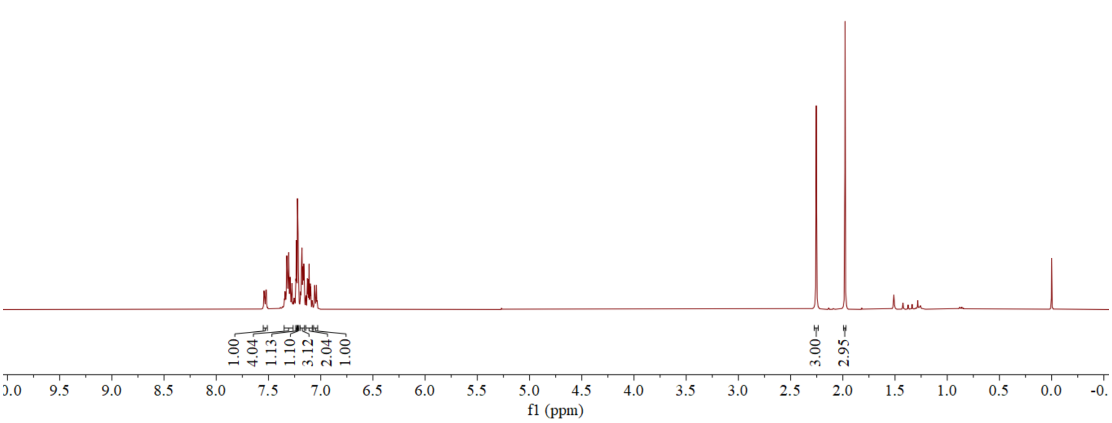

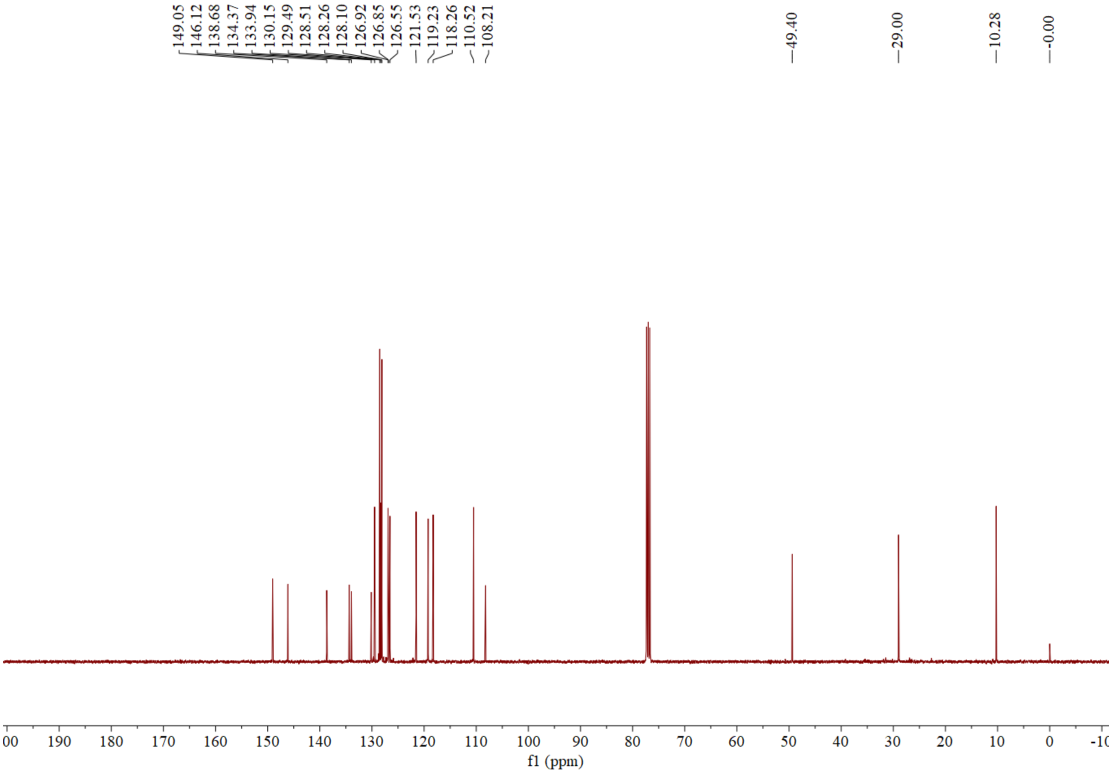

3-methyl-2-(1-(naphthalen-1-yl)-1-phenylethyl)-1*H*-indole (4g):

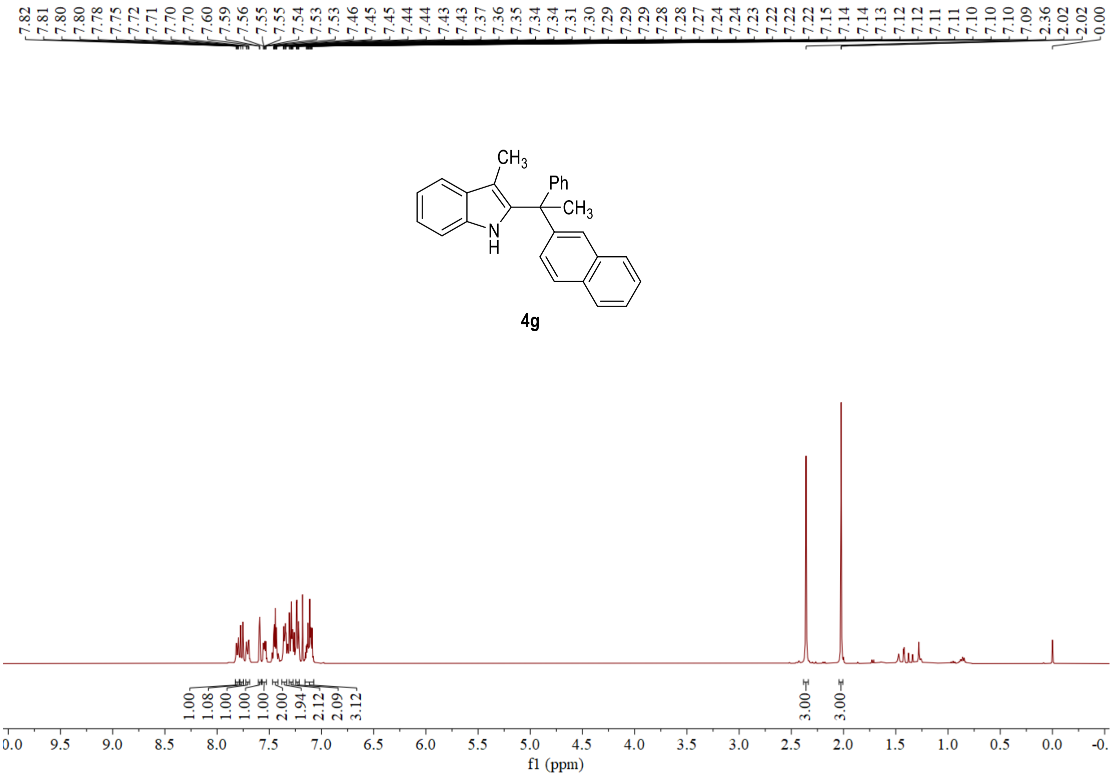

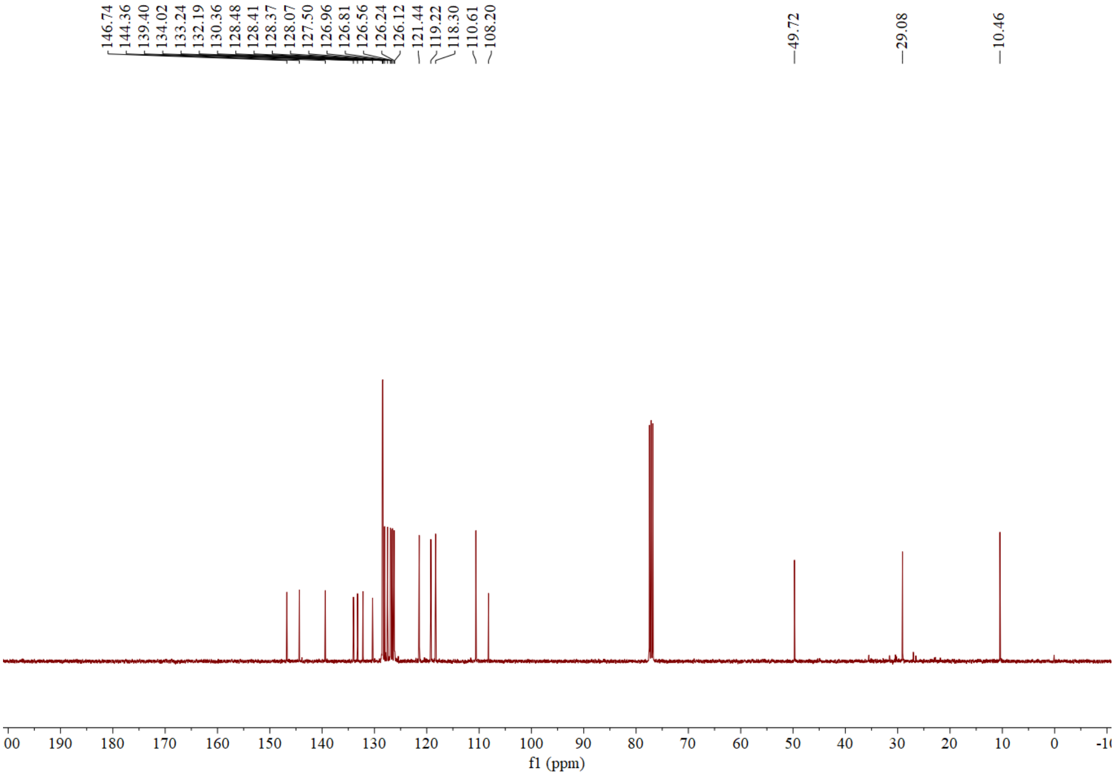

3-methyl-2-(1-phenyl-1-(thiophen-2-yl)ethyl)-1*H*-indole (4h):

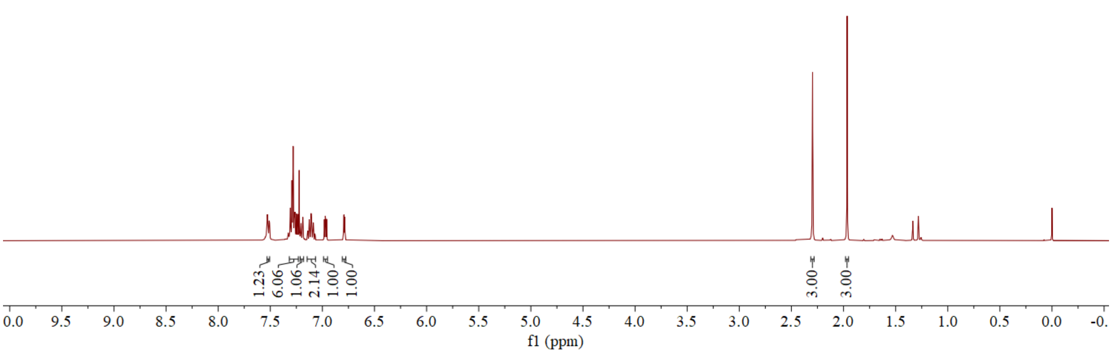

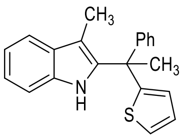

4h

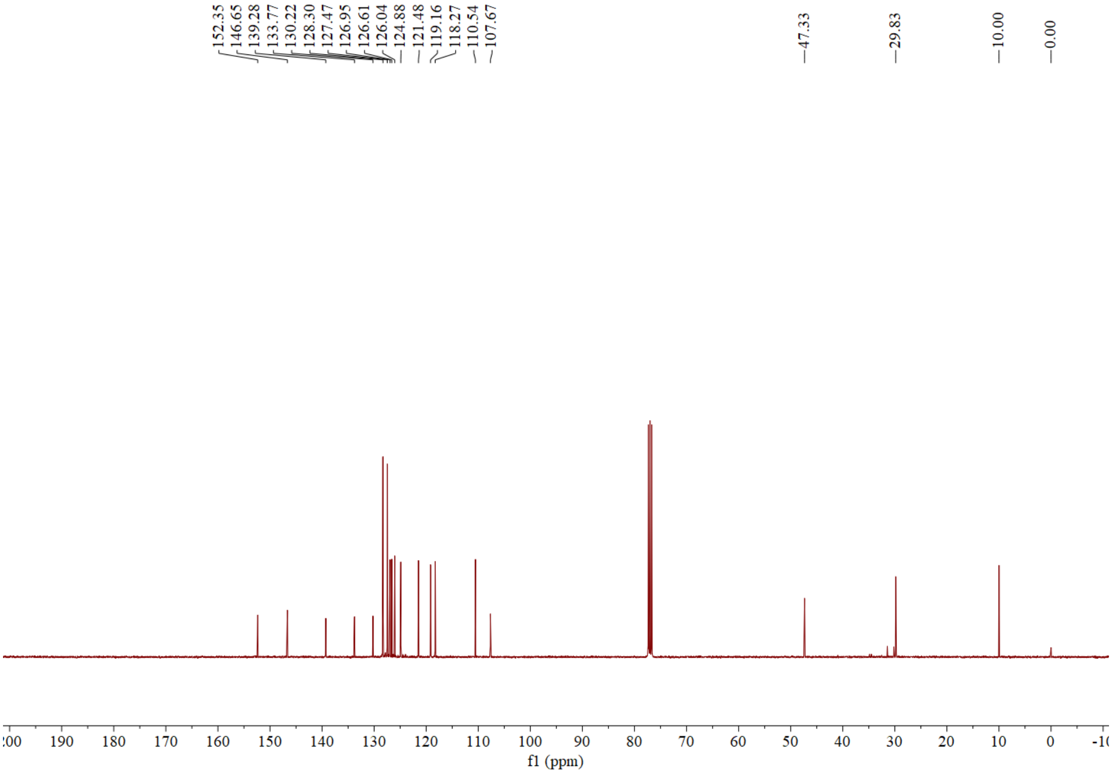

2-(2-([1,1'-biphenyl]-4-yl)propan-2-yl)-3-methyl-1*H*-indole (4i):

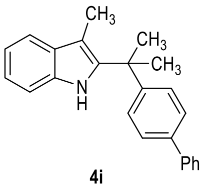

4i

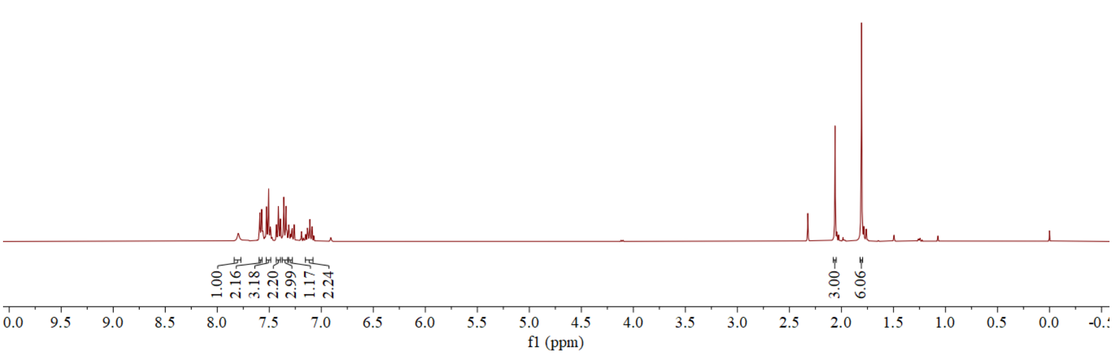

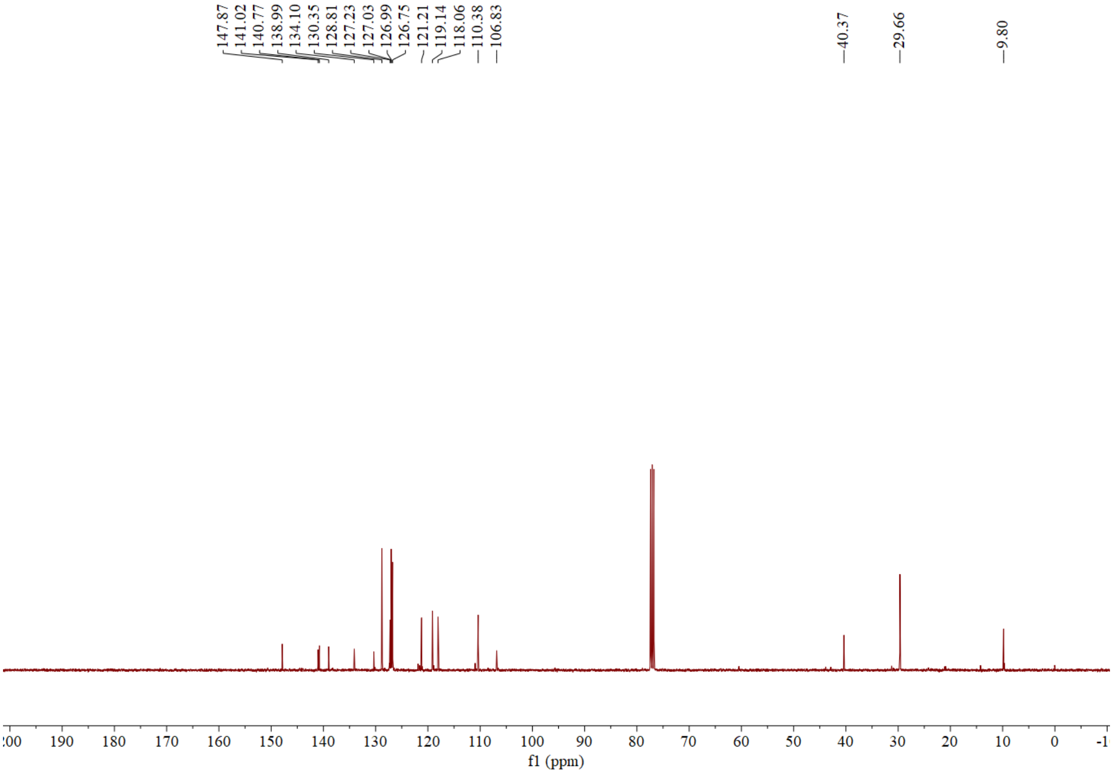

2-(2-(4-chlorophenyl)propan-2-yl)-3-methyl-1*H*-indole (4j):

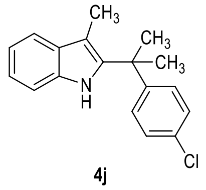

4j

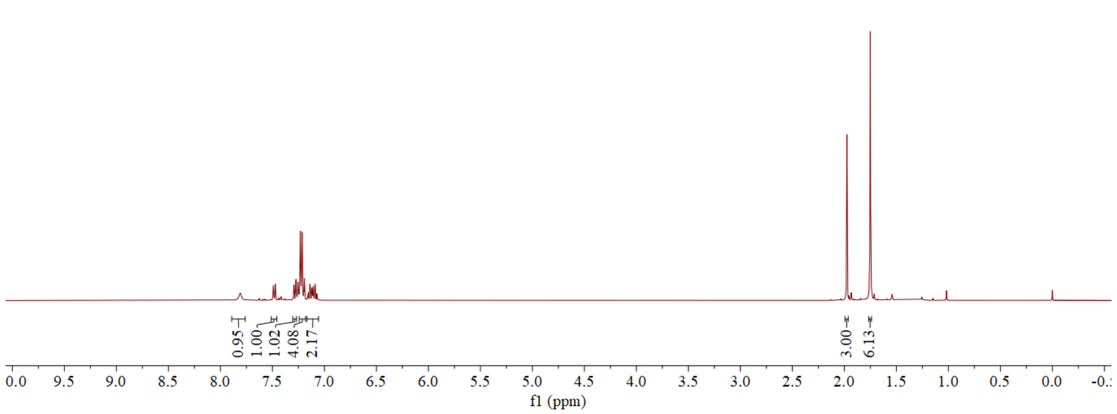

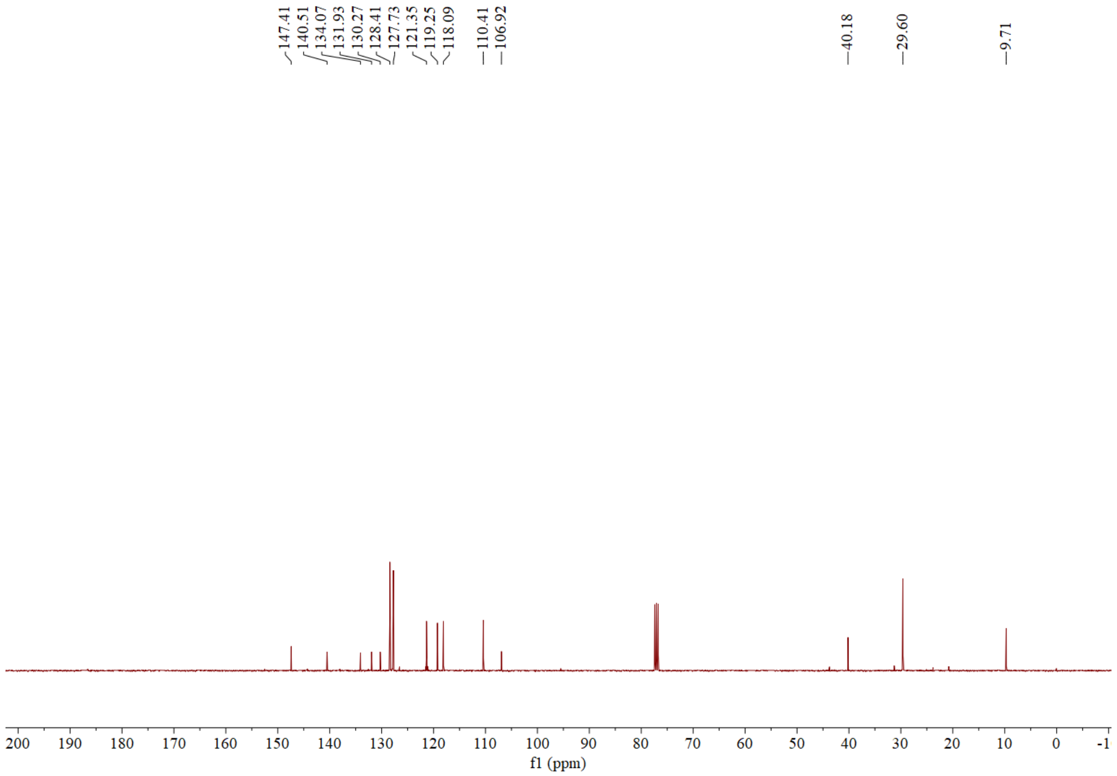

2-(1,1-diphenylpropyl)-3-methyl-1*H*-indole (4k):

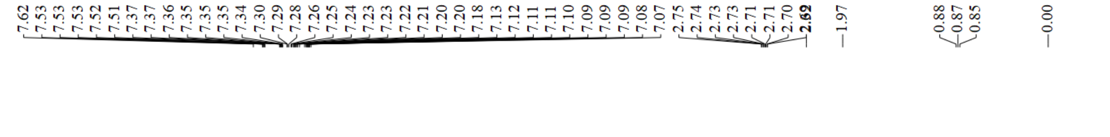

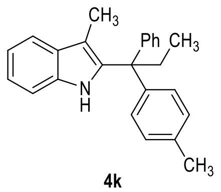

4k

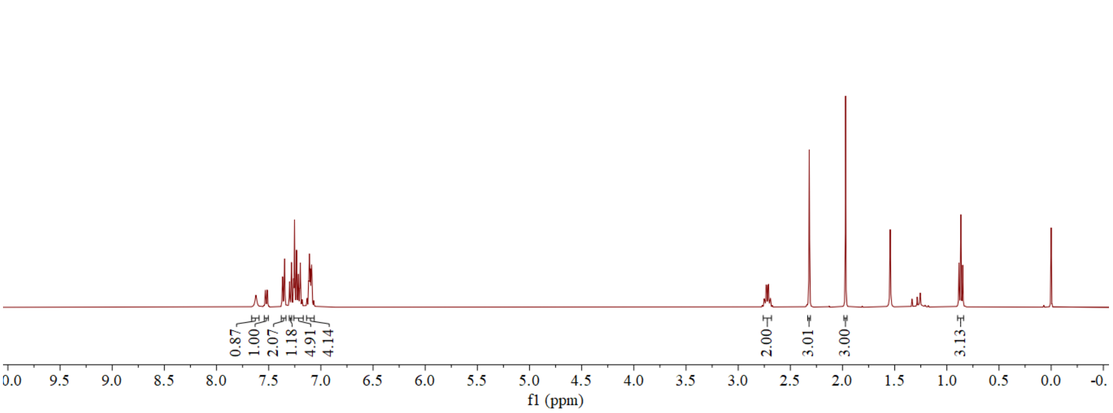

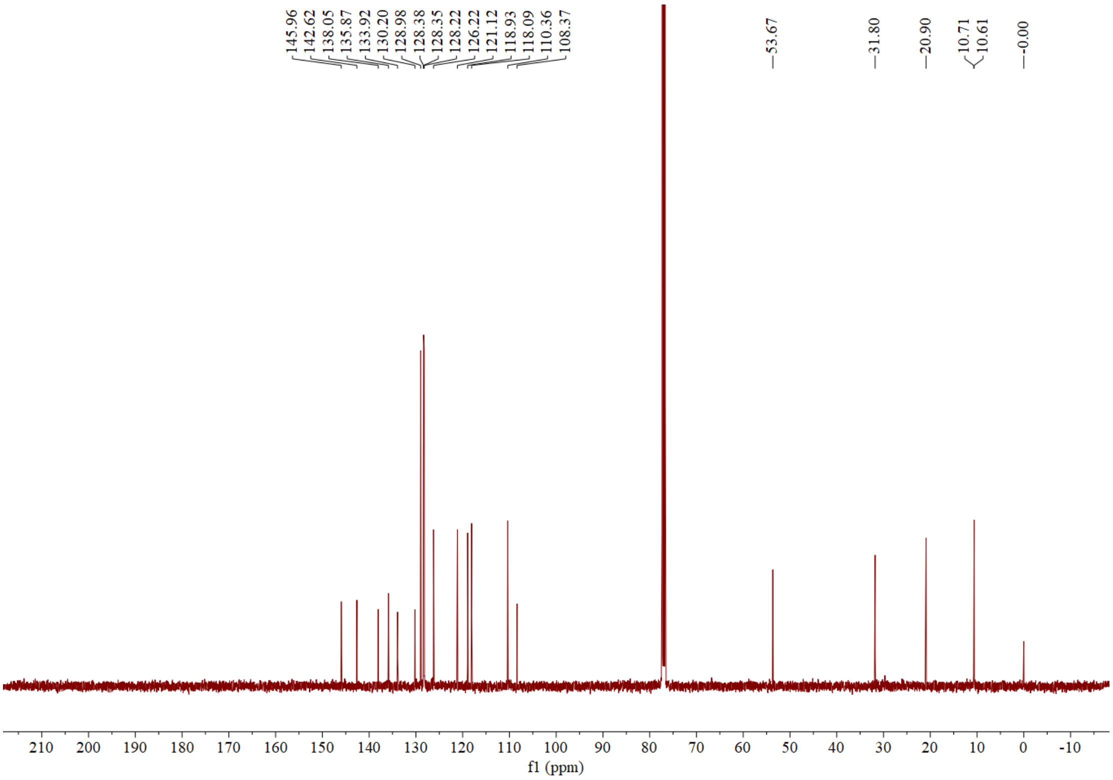

2-(1,1-di-p-tolylethyl)-3-methyl-1*H*-indole (4l):

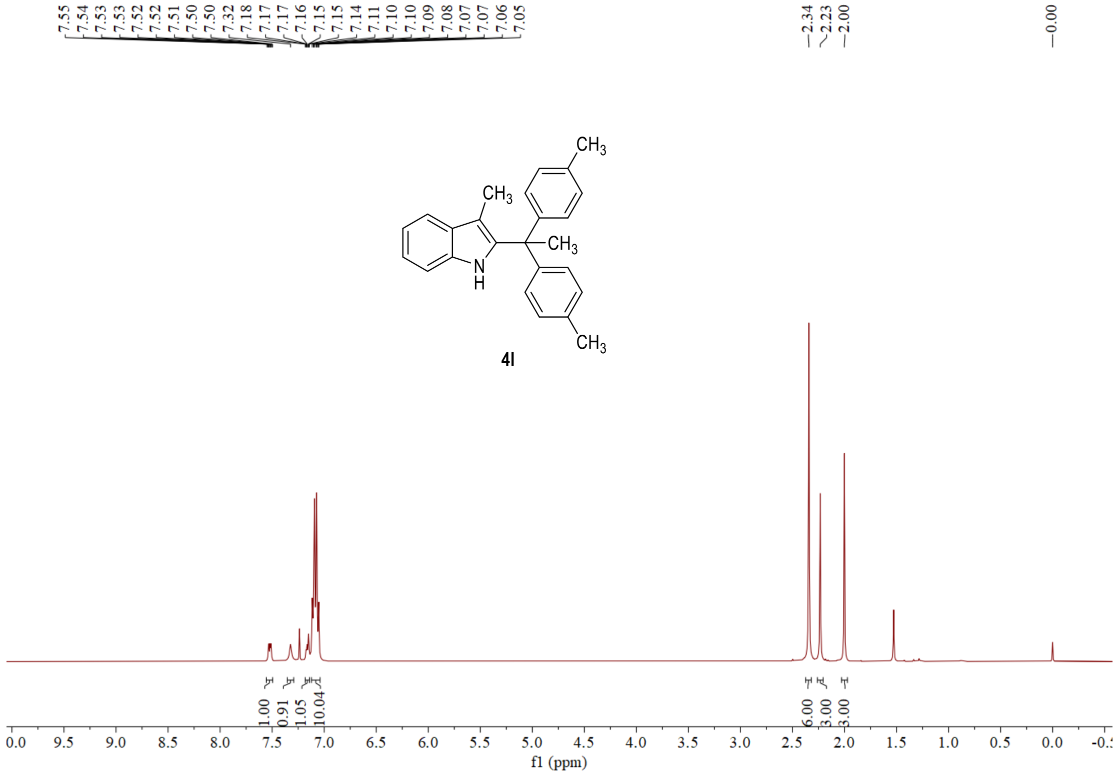

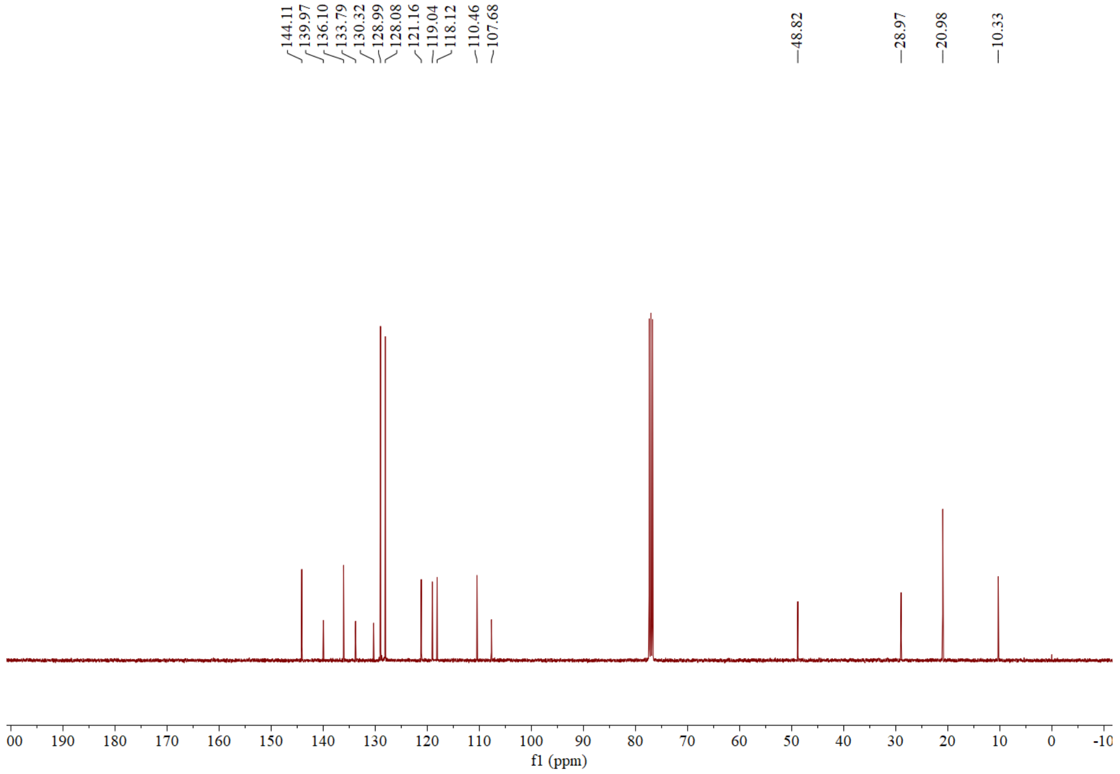

2-(1,1-bis(4-fluorophenyl)ethyl)-3-methyl-1*H*-indole (4m):

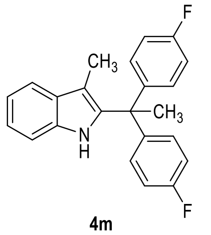

4m

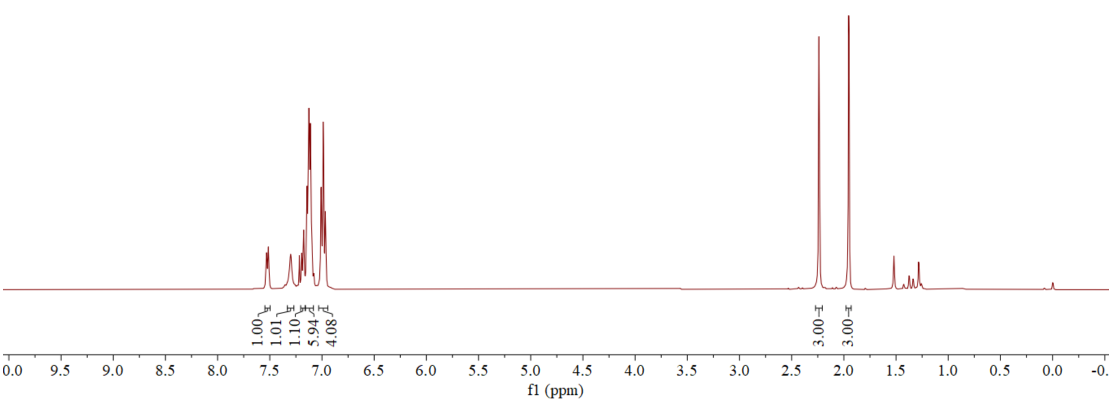

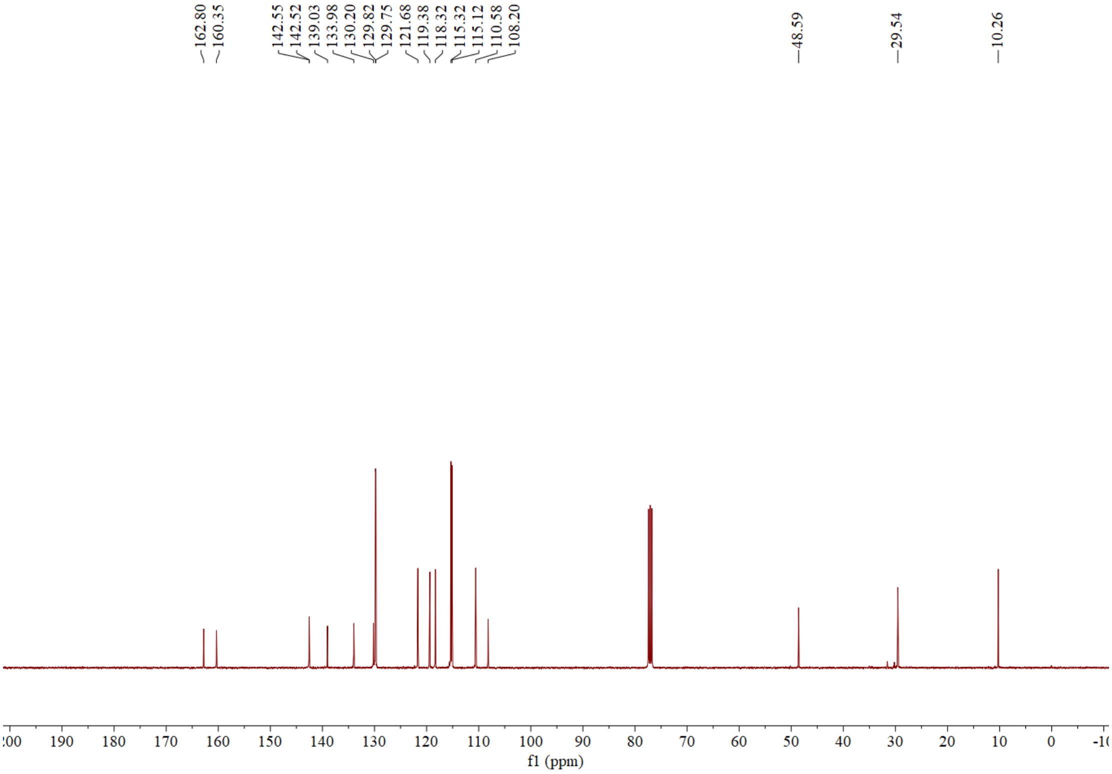

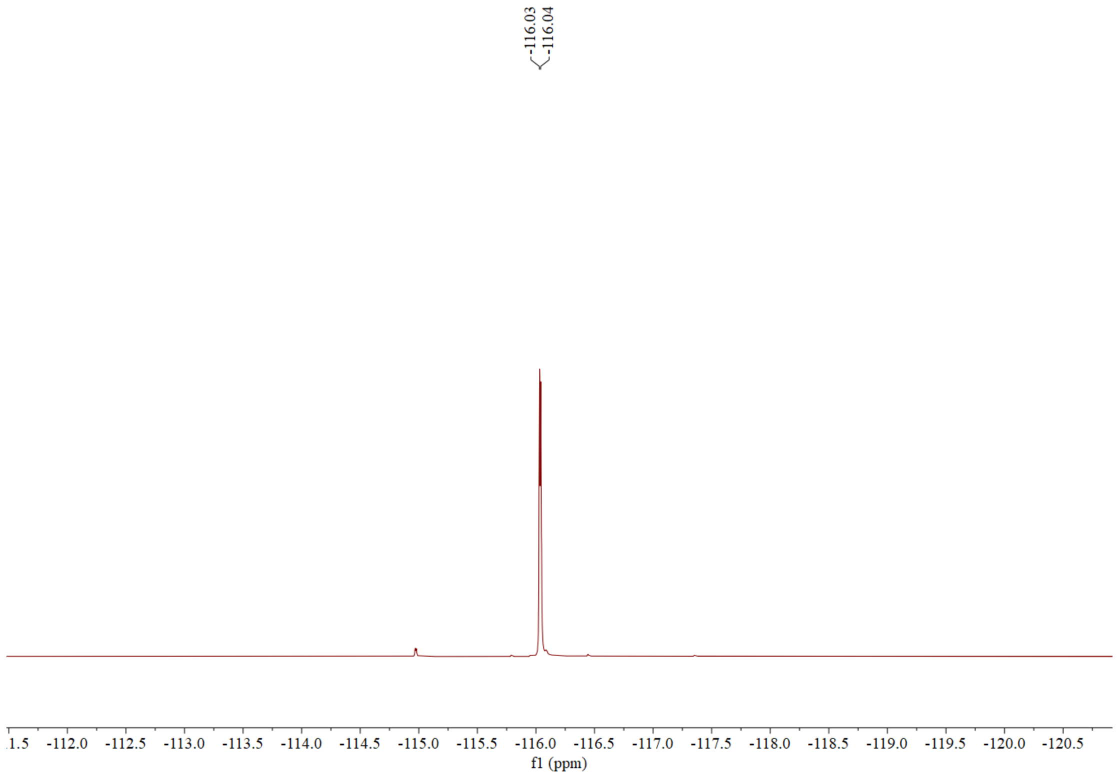
-116.03
-116.04
.1.5 -112.0 -112.5 -113.0 -113.5 -114.0 -114.5 -115.0 -115.5 -116.0 -116.5 -117.0 -117.5 -118.0 -118.5 -119.0 -119.5 -120.0 -120.5
f1 (ppm)

2-(1,1-diphenylethyl)-1,3-dimethyl-1*H*-indole (5):

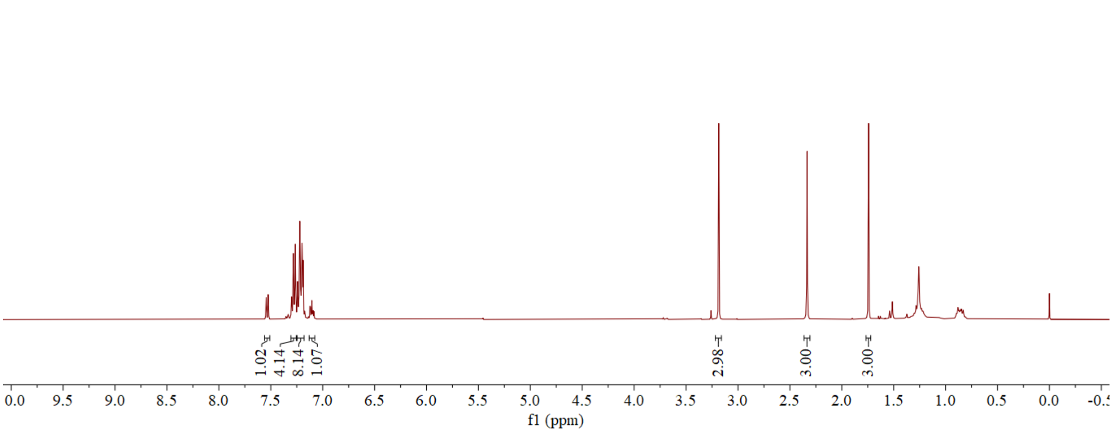

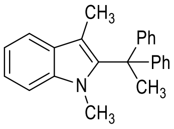

5

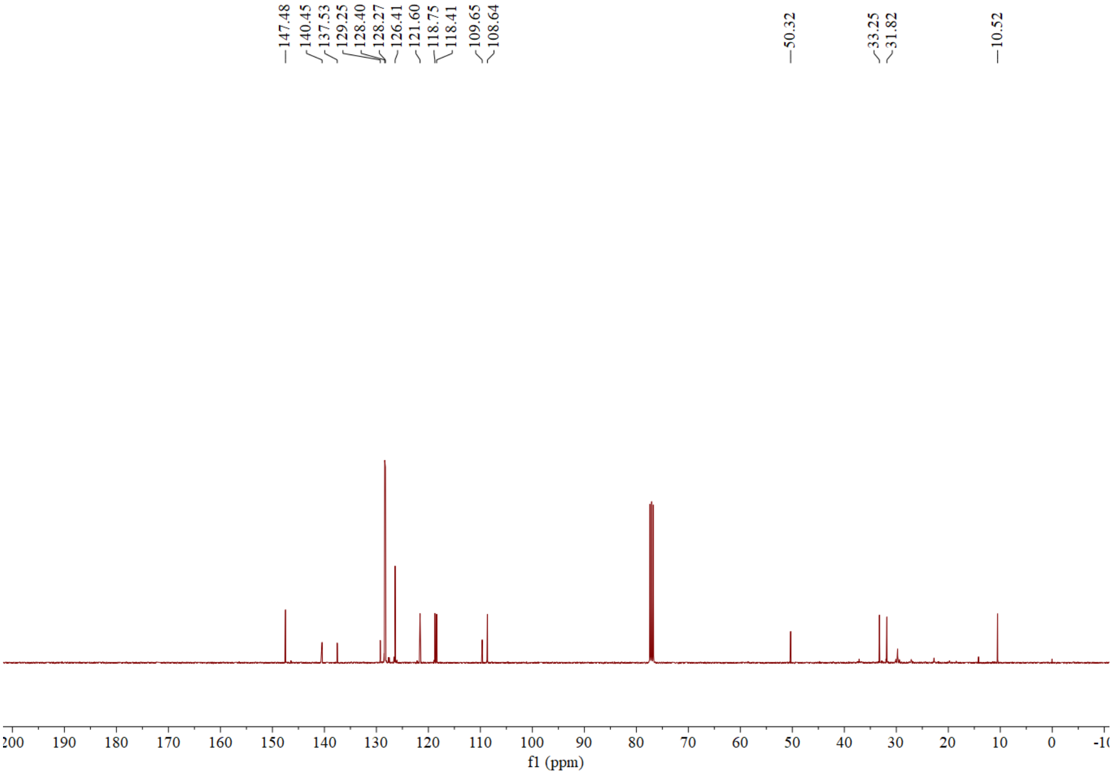

VII. References.

[1] H. Cheng, L. Lu, T. Wang, Q. Yang, X. Liu, Y. Li, Q. Deng, J. Chen, W. Xiao, *Angew. Chem. Int. Ed*. **2013**, *52*, 3250-3254.

[2] J. Bai, L. Zhao, F. Wang, F. Yan, T. Kano, K. Maruoka, Y. Li, *Org. Lett*. **2020**, *22*, 5439-5445.

[3] W. Chen, Y. Xia, L. Lin, X. Yuan, S. Guo, X. Liu, X. Feng, *Chem. Eur. J.* **2015**, *21*, 15104-15107.

[4] M. Devi, A. P. Jadhav, R. P. Singh, *New J. Chem*. **2021**, *45*, 8445-8448.

[5] T. Heller, T. Fu, R. Sarpong, *Org. Lett*. **2012**, *14*, 1970-1973.

[6] R. Beaud, R. Guillot, C. Kouklovsky, G. Vincent, *Chem. Eur. J.* **2014**, *20*, 7492-7500.

[7] C. Huang, R. Ci, J. Qiao, X. Wang, K. Feng, B. Chen, C. Tung, L. Wu, *Angew. Chem. Int. Ed.* **2021**, *60*, 11779-11783.

[8] H. Yang, Y. Yao, M. Chen, Z. Ren, Z. Guan, *J. Am. Chem. Soc*. **2021**, *143*, 7298-7305.

[9] N. Noto, T. Koike, M. Akita, *ACS Catal*. **2019**, *9*, 4382-4387.

[10] C. Chatalova-Sazepin, Q. Wang, G. Sammis, J. Zhu, *Angew. Chem. Int. Ed.* **2015**, *54*, 5443-5446.
