# Supplementary material for: 2-Alkylation of 3-Alkyindoles With Unactivated Alkenes
Source: Front Chem. 2022 Feb 24;10:860764. doi: 10.3389/fchem.2022.860764 (PMC8907451; doi:10.3389/fchem.2022.860764)

checkCIF/PLATON report

Structure factors have been supplied for datablock(s) 201210d

THIS REPORT IS FOR GUIDANCE ONLY. IF USED AS PART OF A REVIEW PROCEDURE FOR PUBLICATION, IT SHOULD NOT REPLACE THE EXPERTISE OF AN EXPERIENCED CRYSTALLOGRAPHIC REFEREE.

No syntax errors found. CIF dictionary Interpreting this report

Datablock: 201210d

```
Bond precision:   C-C = 0.0066 A          Wavelength=0.71073

Cell:          a=9.7991(9)       b=10.114(1)       c=18.1256(16)
               alpha=90.816(2)   beta=105.683(4)   gamma=104.018(1)
Temperature:   293 K

                Calculated                  Reported
Volume          1671.9(3)                   1671.9(3)
Space group     P -1                        P -1
Hall group      -P 1                        -P 1
Moiety formula  C23 H21 N                   C23 H21 N
Sum formula     C23 H21 N                   C23 H21 N
Mr              311.41                      311.43
Dx,g cm-3       1.237                       1.237
Z               4                           4
Mu (mm-1)       0.071                       0.071
F000            664.0                       664.2
F000'           664.23
h,k,lmax        11,12,21                    11,12,21
Nref            5902                        5803
Tmin,Tmax       0.983,0.989                 0.974,0.989
Tmin'           0.974

Correction method= # Reported T Limits: Tmin=0.974 Tmax=0.989
AbsCorr = MULTI-SCAN

Data completeness= 0.983          Theta(max)= 25.020

R(reflections)= 0.0897( 3402)                        wR2(reflections)=
                                                     0.2536( 5803)
S = 0.920                  Npar= 439
```

The following ALERTS were generated. Each ALERT has the format
test-name_ALERT_alert-type_alert-level.
Click on the hyperlinks for more details of the test.

Alert level C

```
PLAT031_ALERT_4_C Refined Extinction Parameter Within Range ......      3.250 Sigma
PLAT155_ALERT_4_C The Triclinic Unitcell is NOT Reduced ..........     Please Do !
PLAT331_ALERT_2_C Small Aver Phenyl C-C Dist C10      --C15      .       1.36 Ang.
PLAT331_ALERT_2_C Small Aver Phenyl C-C Dist C16      --C21      .       1.37 Ang.
PLAT331_ALERT_2_C Small Aver Phenyl C-C Dist C33      --C38      .       1.37 Ang.
PLAT331_ALERT_2_C Small Aver Phenyl C-C Dist C39      --C44      .       1.37 Ang.
PLAT334_ALERT_2_C Small Aver. Benzene C-C Dist C26      -C31             1.37 Ang.
PLAT340_ALERT_3_C Low Bond Precision on  C-C Bonds ...............    0.00662 Ang.
PLAT420_ALERT_2_C D-H Bond Without Acceptor  N1       --H1       .     Please Check
PLAT420_ALERT_2_C D-H Bond Without Acceptor  N2       --H2       .     Please Check
PLAT906_ALERT_3_C Large K Value in the Analysis of Variance ......      5.360 Check
PLAT911_ALERT_3_C Missing FCF Refl Between Thmin & STh/L=    0.595         98 Report
PLAT921_ALERT_1_C R1  in the CIF and FCF Differ by ...............     0.0023 Check
```

Alert level G

```
PLAT066_ALERT_1_G Predicted and Reported Tmin&Tmax Range Identical          ? Check
PLAT072_ALERT_2_G SHELXL First  Parameter in WGHT  Unusually Large       0.17 Report
PLAT073_ALERT_1_G H-atoms ref, but _hydrogen_treatment Reported as     constr Check
PLAT199_ALERT_1_G Reported _cell_measurement_temperature ..... (K)        293 Check
PLAT200_ALERT_1_G Reported   _diffrn_ambient_temperature ..... (K)        293 Check
PLAT769_ALERT_4_G CIF Embedded explicitly supplied scattering data     Please Note
PLAT870_ALERT_4_G ALERTS Related to Twinning Effects Suppressed ..          ! Info
PLAT882_ALERT_1_G No Datum for _diffrn_reflns_av_unetI/netI ......     Please Do !
PLAT883_ALERT_1_G No Info/Value for _atom_sites_solution_primary .     Please Do !
PLAT910_ALERT_3_G Missing # of FCF Reflection(s) Below Theta(Min).          1 Note
PLAT933_ALERT_2_G Number of OMIT Records in Embedded .res File ...         16 Note
PLAT941_ALERT_3_G Average HKL Measurement Multiplicity ..........         1.0 Low
PLAT962_ALERT_5_G Number  FCF File  Relections with Sigma(I) = 0.0          5 Note
```

0 **ALERT level A** = Most likely a serious problem - resolve or explain
0 **ALERT level B** = A potentially serious problem, consider carefully
13 **ALERT level C** = Check. Ensure it is not caused by an omission or oversight
13 **ALERT level G** = General information/check it is not something unexpected

7 ALERT type 1 CIF construction/syntax error, inconsistent or missing data
9 ALERT type 2 Indicator that the structure model may be wrong or deficient
5 ALERT type 3 Indicator that the structure quality may be low
4 ALERT type 4 Improvement, methodology, query or suggestion
1 ALERT type 5 Informative message, check

It is advisable to attempt to resolve as many as possible of the alerts in all categories. Often the minor alerts point to easily fixed oversights, errors and omissions in your CIF or refinement strategy, so attention to these fine details can be worthwhile. In order to resolve some of the more serious problems it may be necessary to carry out additional measurements or structure refinements. However, the purpose of your study may justify the reported deviations and the more serious of these should normally be commented upon in the discussion or experimental section of a paper or in the "special_details" fields of the CIF. checkCIF was carefully designed to identify outliers and unusual parameters, but every test has its limitations and alerts that are not important in a particular case may appear. Conversely, the absence of alerts does not guarantee there are no aspects of the results needing attention. It is up to the individual to critically assess their own results and, if necessary, seek expert advice.

Publication of your CIF in IUCr journals

A basic structural check has been run on your CIF. These basic checks will be run on all CIFs submitted for publication in IUCr journals (*Acta Crystallographica*, *Journal of Applied Crystallography*, *Journal of Synchrotron Radiation*); however, if you intend to submit to *Acta Crystallographica Section C* or *E* or *IUCrData*, you should make sure that full publication checks are run on the final version of your CIF prior to submission.

Publication of your CIF in other journals

Please refer to the *Notes for Authors* of the relevant journal for any special instructions relating to CIF submission.

PLATON version of 13/07/2021; check.def file version of 13/07/2021

Datablock 201210d - ellipsoid plot

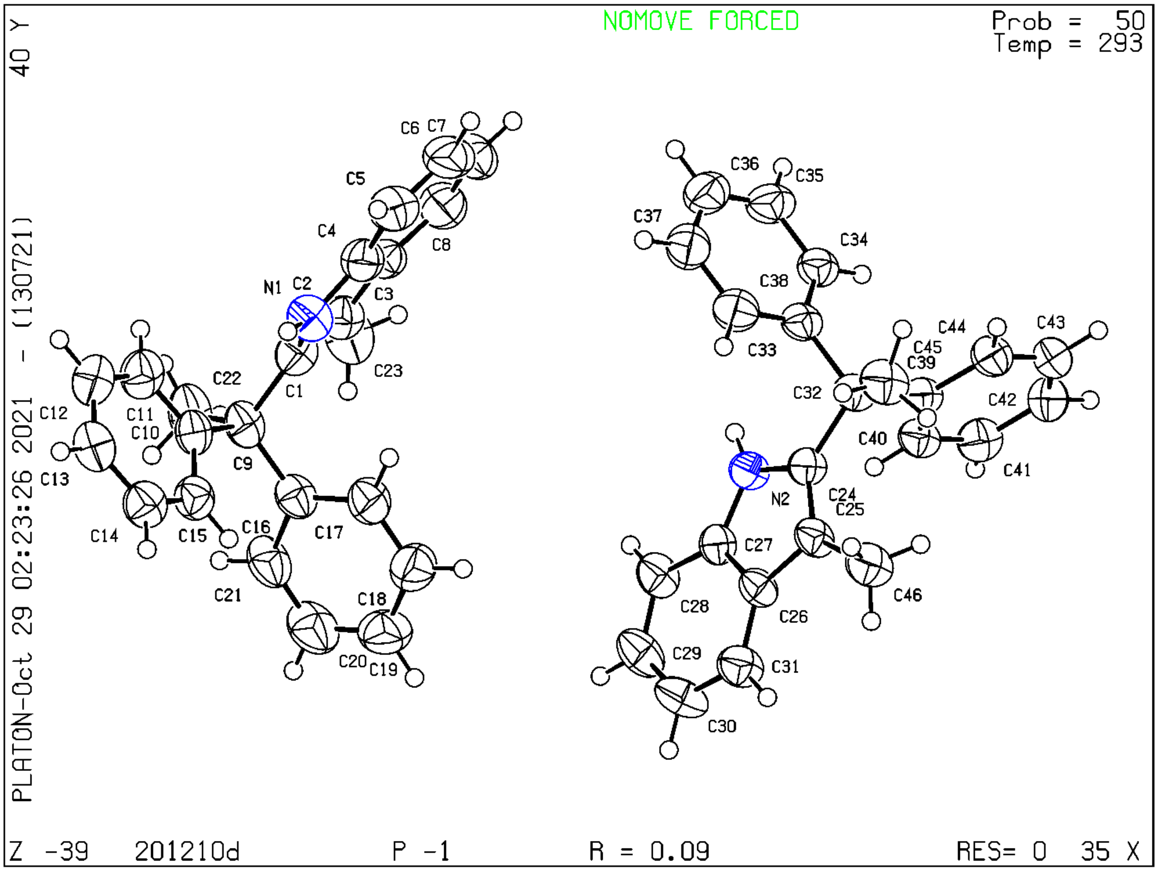

Supplement: Supplementary file 2 [file DataSheet1.PDF]
